# BIMS-GA: Adaptive Multi-Objective Optimization for Network-Aware Pancreatic Ductal Adenocarcinoma Biomarker Discovery

**DOI:** 10.3390/life16101716

**Published:** 2026-10-14

**Authors:** Teerapun Saeheaw

**Affiliations:** Department of Teacher Training in Mechanical Engineering, King Mongkut’s University of Technology North Bangkok, Bangkok 10800, Thailand; teerapun.s@fte.kmutnb.ac.th

**Keywords:** pancreatic ductal adenocarcinoma, multi-objective genetic algorithm, feature selection, protein–protein interaction network, biomarker discovery

## Abstract

Pancreatic ductal adenocarcinoma (PDAC) has a five-year relative survival of 8%. Feature selection optimizes accuracy, survival concordance, and network structure separately; whether uniting them matters has not been determined. The BIMS-GA optimizes the cross-validated AUC, Cox concordance, and STRING v11.0 protein–protein interaction coherence in one fitness function and was compared against fourteen alternative methods on GSE62452 (*n* = 130) and eight external evaluation sets. Using a nested protocol, the fifteen methods had pooled out-of-fold AUCs spanning 0.877–0.900; the BIMS-GA ranked thirteenth with 0.8855. No DeLong comparisons survived correction. Stability did not persist either: the ten-seed Jaccard index of 0.804 exceeded every comparator but fell to 0.521 in a second seed block. Network coherence separated them by two orders of magnitude, 0.480 against 0.005–0.192, in an ordering unbroken across twenty runs. Removing the network term raised the discovery AUC by 0.0117 and lowered external prognostic concordance by 0.0149 in all ten runs, acting as a regularizer. The twenty-gene panel comprises one laminin-332 and an integrin adhesion program with no isolated member, stratifying survival in four cohorts (hazard ratios of 2.92–4.13 fitted within each cohort; 1.02–2.17 transferred). Network coherence, not discrimination, is the axis that remained separate in every run examined, while short-run stability did not.

## 1. Introduction

Pancreatic ductal adenocarcinoma (PDAC) is among the most lethal malignancies worldwide. Global estimates for 2022 recorded approximately 511,000 new cases and 467,000 deaths, making it the sixth leading cause of cancer mortality across both sexes [1]. About nine in ten of those tumors are adenocarcinomas. In the United States, the five-year relative survival rate for pancreatic cancer is 13%, ranking it among the lowest of all major cancer types, and 8% for adenocarcinoma. The increase in the overall figure from 4% in the mid-1990s is partly a result of incidentally detected, well-differentiated neuroendocrine tumors [2]. The principal contributor to mortality is late diagnosis: most cases are identified at an advanced or metastatic stage when curative resection is no longer feasible [3]. Carbohydrate antigen 19-9 remains the only serum biomarker approved by the US Food and Drug Administration for monitoring pancreatic cancer, but its sensitivity and specificity preclude its use as a standalone early diagnostic tool [3,4]. Transcriptomic panels that stratify patients by diagnostic class and prognostic risk therefore remain a clinically important computational target.

Gene expression profiling of tumor and adjacent non-tumor tissue offers a data-rich foundation for biomarker discovery in PDAC. High-throughput microarray platforms have generated publicly available datasets that capture genome-wide transcriptional differences between tumor and normal pancreatic tissue across independent patient cohorts [3]. These datasets typically profile tens of thousands of genes across a few hundred samples. Such dimensionality demands principled feature selection to identify compact gene panels suitable for diagnostic and prognostic modeling.

Established feature selection methods for cancer transcriptomics fall into three categories: filter approaches include the ANOVA F-test and minimum redundancy maximum relevance (mRMR), wrapper approaches include support vector machine recursive feature elimination (SVM-RFE), and regularization-based approaches include the least absolute shrinkage and selection operator (LASSO). A systematic review of feature selection on microarray data grouped 132 studies into nine research types, categorized by objective and compared according to their methods, classifiers, datasets, performance metrics, and results [5]. These studies investigated cancer classification; survival-related and network-based criteria lay outside their scopes. Genes selected for maximal diagnostic accuracy do not necessarily form biologically coherent networks or predict patient survival, and genes selected for coherence or for survival do not necessarily discriminate [6,7]. The practical consequences of this are rarely quantified because panels are seldom evaluated against criteria other than the ones used to select them.

Metaheuristic optimization algorithms, including genetic algorithms, particle swarm optimization, and the grey wolf optimizer, have been applied to high-dimensional gene selection in cancer transcriptomics [8,9]. These population-based search methods can escape local optima and suit combinatorial feature selection over large gene pools. Many of these implementations optimize a single fitness objective, usually cross-validated classification accuracy, without prognostic or network coherence criteria. Single-objective metaheuristic selection therefore exhibits the same limitations as filter and wrapper approaches.

Multi-objective evolutionary algorithms address these limitations by optimizing several competing criteria simultaneously and identifying Pareto-optimal solutions that represent principled trade-offs between them [10]. When applied to biomarker discovery, these methods agree on the first criterion but differ on the second. The first is almost always discrimination; accuracy, area under the curve, sensitivity, and balanced accuracy are four measurements occupying one axis rather than four criteria. When a genuinely different second criterion is added, it is usually structural. INMOMBD optimizes diagnostic accuracy, disease–gene association, and intra-module link density derived from protein–protein interaction (PPI) networks [11]. DM-MOGA targets network module cohesion in co-expression graphs for lung cancer [12], and MOGAMUN maximizes differential expression and interaction density in multiplex biological networks [13]. These methods differ in which structure they prefer rather than in what they are ultimately selecting for.

None of these frameworks link survival to the other two criteria. PPI networks such as those provided by the STRING database encode known and predicted functional associations between proteins across organisms [14]. Incorporating that topology as a feature-selection criterion promotes panels whose products share functional interactions, which has been reported to improve the stability of the selected set for some methods rather than for network-based approaches as a class [15]. Weighting genes by their network topology has also been reported to improve prognostic accuracy on independent cohorts, in glioblastoma, esophageal squamous cell carcinoma, and colorectal cancer [7]. Prognostic gene signatures for PDAC have been validated by Kaplan–Meier survival analysis and Harrell’s concordance index (C-index) across independent Gene Expression Omnibus (GEO) and The Cancer Genome Atlas (TCGA) cohorts [16,17]. The studies that optimize survival, however, optimize it alone: none includes a diagnostic objective in the same search, and the one case in which a network accompanied a survival objective had a non-PDAC methodological precedent [7]. The single framework that placed survival concordance beside a discrimination objective within one search carried no network term [18]. The three criteria therefore appear in pairs at most and never together, and a search that never holds all three cannot report what prioritizing one of them costs the other two.

Three gaps in the current research emerge, as shown in Table 1 below. First, no method listed optimizes diagnostic accuracy, prognostic concordance, and network coherence together within the search; each addresses at most two. Second, comparisons between selection methods are almost always reported on discrimination alone, so the cost of prioritizing one criterion over another remains unquantified. Third, the reproducibility of a selected panel across repeated runs is seldom reported, even though a panel that changes between runs cannot be carried forward as a candidate signature.

The present study addresses these gaps through the following contributions.

1. Evaluation beyond discrimination: Eighteen panels of equal size are scored on the same cohort under the same models. They come from the Biologically Informed Multi-objective Survival Genetic Algorithm (BIMS-GA) proposed here, its three ablation variants, and fourteen alternative methods. They are compared on discrimination, selection stability, network topology, calibration, and transfer to eight external cohorts, and net benefit is reported for the panel selected in this study.

2. BIMS-GA algorithm: A genetic algorithm applied over fixed-size gene subsets that optimizes the cross-validated area under the receiver operating characteristic curve (AUC), Cox concordance, and PPI coherence within one fitness function. The relative weight of the first two objectives is adjusted from the correlation observed between them during the search.

3. Measured cost and return of the network objective: The loss in fit to the discovery cohort and the gain in external prognostic concordance are estimated as paired differences over ten random seeds.

4. Leakage-controlled protocol: Feature screening and selection are repeated inside every outer training partition, and the resulting estimate is compared with the full-dataset estimate for BIMS-GA and for three Pareto-based comparators.

The BIMS-GA computes its three objectives as follows: The diagnostic objective is the five-fold cross-validated AUC for PDAC versus non-tumor classification. The prognostic objective is the concordance index of a ridge-penalized Cox proportional hazards model fitted on the survival data of the same cohort. The network objective is PPI coherence, which is the mean STRING v11.0 interaction confidence over the selected pairs. Selection is performed on GSE62452 alone. The resulting twenty-gene panel is compared with fourteen alternative methods spanning filter, wrapper, metaheuristic, machine-learning, deep-learning, and Pareto-based families and with three ablation variants of BIMS-GA itself. It is then evaluated in four external diagnostic cohorts and four external prognostic cohorts, none of which contributed to any stage of selection, and characterized according to functional enrichment and network topology.

## 2. Methodology

### 2.1. Datasets and Pre-Processing

**Figure 1:**
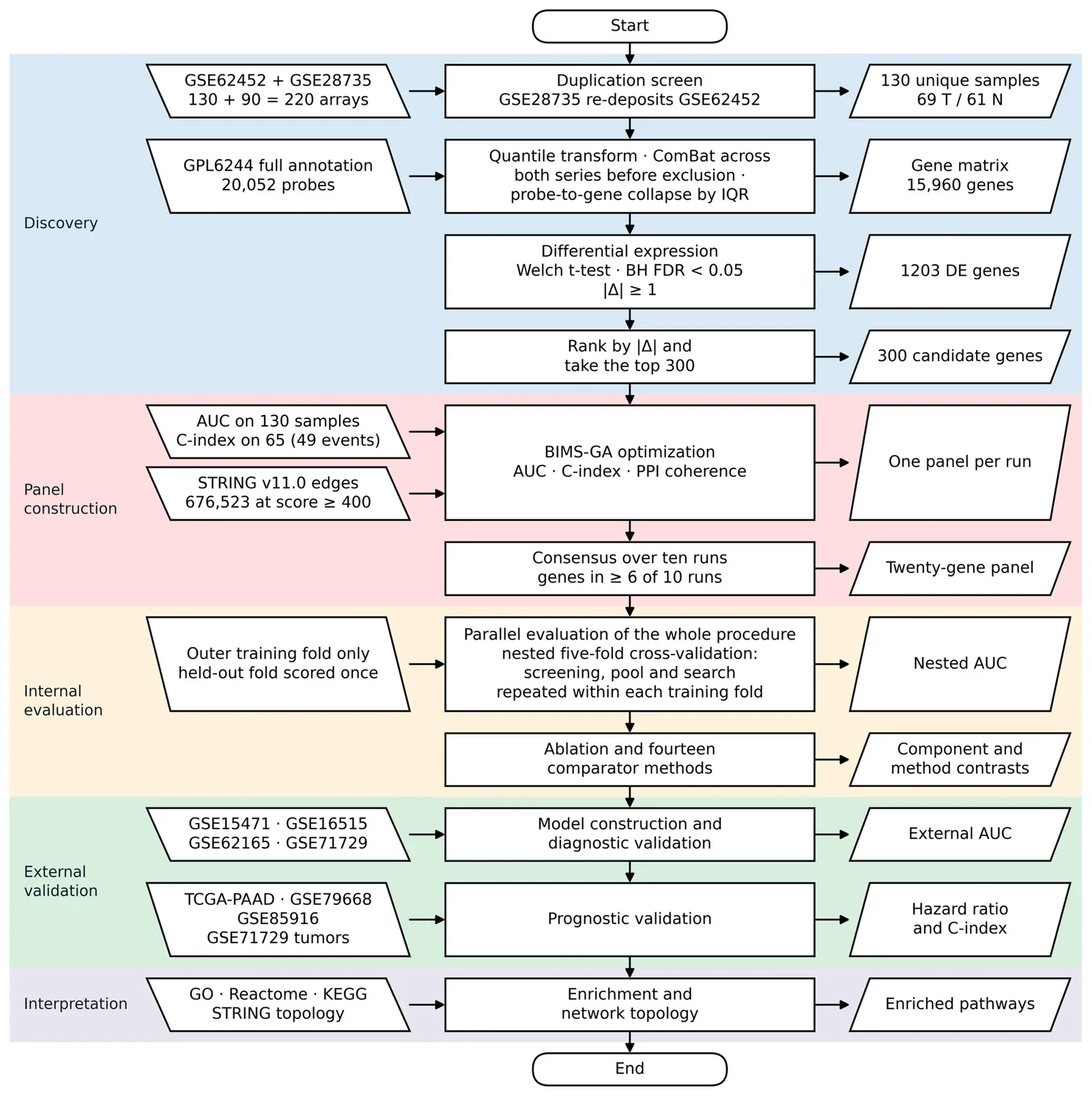
Analysis workflow. Shading marks five phases: discovery (blue), panel construction (pink), internal evaluation (yellow), external validation (green), and interpretation (purple). Parallelograms leaning right are inputs, rectangles are processes, and parallelograms leaning left are outputs.

Transcriptomic datasets were retrieved from GEO and TCGA. Figure 1 illustrates the complete analysis workflow. Feature selection used GSE62452 alone, profiled on the Affymetrix Human Gene 1.0 ST Array (GPL6244), comprising 69 tumor and 61 non-tumor samples. Survival time and event status were deposited for 65 of the tumors, of which 49 are events. These supply the prognostic objective, so both objectives are computed for the same patients.

GSE28735 was initially considered a second discovery series. A sample-level screen showed that all 90 of its arrays are redepositions of GSE62452 arrays. Every pair differs by a constant offset of 815,201 in the GEO sample (GSM) accession number; the assignment between the two series is one-to-one, and tissue labels agree in all 90 pairs. Matched-pair correlations across all 28,869 common probes range from 0.9845 to 0.9907. Correlation alone does not establish redeposition in a series of one tissue type, since correlations within each series reach 0.9880; the accession offset and the one-to-one assignment are what identify it. Restriction to the 5000 most variable probes reaches the same conclusion by a different route and with wider separation; absolute correlations decrease to a range of 0.9738 to 0.9831. The minimum margin between each matched pair and its runner-up widens from 0.0070 to 0.0222, and the number of matched pairs exceeding the within-series maximum rises from 84 to 88 of 90. If both series were retained, 90 patients would have been counted twice, so GSE28735 was excluded, and the discovery cohort was reduced from 220 to 130 samples.

Four probe counts describe this platform. GSE62452 carries 33,297 probe sets, of which 28,869 are also present in GSE28735; the containment is one-way, since every GSE28735 probe is present in GSE62452 while 4428 of GSE62452′s are not in GSE28735. Of those 28,869, some 20,052 carry a GPL6244 annotation, and these collapse to 15,960 distinct genes.

The same screen was applied to all 36 pairs in the discovery series and the eight evaluation sets, including the two RNA-sequencing prognostic cohorts (Table S1A Panel B). In 35 of the 36, no cross-series sample pair correlates above the ceiling set by the most similar pair within either series. The exception is the pair formed by the diagnostic and prognostic subsets of GSE71729, where 123 sample pairs correlate at 1.0000 because the prognostic subset consists of 123 of the 145 tumors in the diagnostic subset, and the two are never combined in one analysis.

External diagnostic validation required a cohort containing both PDAC tumor and non-tumor pancreatic tissue profiled on a platform other than the discovery array, with at least ten samples per class and probe-level annotation permitting gene matching. Four series met these requirements: GSE15471, GSE16515, GSE62165 and GSE71729. External prognostic validation additionally required deposited survival time and event status and the absence of any sample from the discovery series. Four cohorts met these conditions: TCGA-PAAD, GSE79668, GSE85916, and the tumor subset of GSE71729, which contributes one diagnostic and one prognostic evaluation set from a single series. GSE15471 contains 78 arrays from 36 patients, 6 of which are technical replicates; 1 array per patient and tissue was retained for 72 in total. Adjacent normal samples in TCGA-PAAD were excluded from all survival analyses. Series examined but not analyzed are listed in Table S1B alongside the reason for their exclusion; among these are the 90 GSE28735 arrays that entered the duplication screen, as shown in Figure 1. The composition of every cohort is reported in Table 2. No cohort in the external set contributed to differential expression screening, to candidate pool construction, or to the genetic algorithm.

Series matrix files were retrieved for each GEO series. Expression values were rank-transformed to a normal distribution by a quantile transformation applied per probe across samples. This step and the assembly of the series matrices precede the gene-level matrix on which everything reported in this study operated. They were not part of the analysis chain that produces the reported numbers, which begins from the gene-level matrix. The full transformation is reported above and can be reimplemented according to this description. Probe identifiers were mapped to gene symbols using the complete GPL6244 annotation table, which covers 20,052 probes—the same 20,052 identified above—and 19,036 gene symbols. Probes matching more than one gene were discarded. When several probes matched the same gene, the probe with the highest interquartile range (IQR) across samples was retained. This yielded a gene-level matrix of 15,960 genes by 130 samples, on which all subsequent operations were carried out.

Because the two series were processed together before the duplication was identified, the discovery matrix carries a batch adjustment computed across both using ComBat [27]. As the series share identical per-probe distributions, this adjustment, reduced to a single global scale factor, was applied uniformly to every probe. Feature standardization within each cross-validation fold absorbed an affine rescaling of this kind. The adjustment was therefore computed across a series that the duplication screen then excluded, after which the discovery result remained unchanged. Per-probe values before and after correlate at 1.0000, as do the t-statistics and the group-mean differences. The candidate pool contains the same 300 genes under both, and the panel’s out-of-fold AUC is identical to four decimal places. The candidate pool and the out-of-fold estimate obtained from the unadjusted matrix are reported beside those obtained from the adjusted matrix in Table S1C.

Differentially expressed genes between tumor and non-tumor tissue were identified in the discovery cohort using Welch’s *t*-test [28], with the false discovery rate controlled at 0.05 using the Benjamini–Hochberg procedure [29]. Genes with an adjusted *p*-value below 0.05 and an absolute difference in group means of at least one were retained, for a total of 1203 of 15,960 genes. At the probe level, the same screen retained 1106 probes on the adjusted matrix against 945 on the unadjusted one, with the two sets agreeing on a Jaccard index of 0.8544. This is the only quantity the adjustment changed: the adjustment is a single global scale factor of 1.0238, and the effect-size threshold is the step it moves (Table S1C). The 300 highest-ranked genes are the same set under both. Because the quantile transformation places every probe on a common scale, this difference is a standardized mean difference rather than a log2 fold change, and the threshold of one corresponds to one standard deviation. The retained genes were ranked according to the absolute difference, and the 300 ranked highest formed the candidate pool over which the genetic algorithm searched. The implementation produces that set in two steps, taking the 500 highest-ranked and then retaining the first 300, which selects the same 300 genes as taking 300 directly.

### 2.2. BIMS-GA: Algorithm Design

The BIMS-GA is a genetic algorithm that optimizes three objectives within one fitness function: the diagnostic AUC, prognostic concordance and network coherence. A chromosome is a set of exactly *k* gene indices drawn from the candidate pool, and every operator preserves that cardinality, so the search moves over fixed-size subsets rather than over binary strings. The panel size *k* = 20 was fixed in advance and was not tuned on any outcome. Table S4B reports the sweep over *k* of 10, 15, 20 and 25, across which discrimination and network coherence move in opposite directions; twenty sits inside that trade-off rather than at either end of it. Twenty genes also sit within the size range commonly used for targeted panels in clinical translation, such as multiplex quantitative PCR (qPCR) assays, while remaining large enough to support a connected network module. The full procedure is described by Algorithm 1.
**Algorithm 1:**  BIMS-GAInput:  *C* = {*g1*, …, *gN*}, *N* = 300 candidate gene pool Output:  S* subset of *C* with |S*| = *k* Parameters: *P* = 40, *T* = 80, *k* = 20, elite = 0.15, tournament size 3,        *p_c* = 0.80, *p_m* = 0.25, *w*_3_ = 0.20, *w*_4_ = 0.05,        *rho_thresh* = 0.30, *W* = 8, seed = 42 1 Initialize *P0* with *P* chromosomes, each selecting exactly *k* genes drawn   uniformly at random from *C* without replacement. 2 Evaluate the three objectives of every chromosome, memorizing objective   values on the sorted gene index set. 3 for *t* = 1 to *T* do     a. Compute *rho*(*t*) by Equation (2), set (*w*_1_(*t*), *w*_2_(*t*)) by Equation (3) and normalize     the four weights to sum to one as in Equation (1).     b. Score every chromosome in Pt under the current weights.     c. Carry the floor(elite × *P*) highest-scoring chromosomes into *Pt* + 1     unchanged, at least one being retained.     d. Every tenth generation, apply a hill-climbing local search to the single     best chromosome, proposing up to twelve one-gene exchanges and accepting     an exchange only if it raises the scalarized fitness.     e. Raise the mutation rate above *p_m* in proportion to the shortfall of     distinct chromosomes below three-fifths of the population, to a ceiling     of 0.45.     f. Fill the remainder of *Pt* + 1 by tournament selection, then uniform     crossover on the symmetric difference with probability *p_c*, then swap     mutation of one to three genes. Reject any child identical to a     chromosome already placed in *Pt* + 1 and repeat the draw.     g. Evaluate the new population and rescore the carried elites under the     weights in force.     h. Score every chromosome of *Pt* + 1 under the fixed reference vector     (0.40, 0.40, *w*_3_, *w*_4_), normalized as in Equation (1), and retain the best seen     so far in an archive.     end for 4 Return the archived chromosome with the highest reference score.

Step 2 memorizes objective values so that a repeated chromosome is never evaluated twice. Step 3e leaves *p_m* unchanged once three in five chromosomes are distinct and reaches its ceiling only when every chromosome is identical, so a population collapsing towards a single solution mutates more. In the runs reported here, the population never fell below that proportion and the escalation did not activate. It was specified because a duplicate control weaker than that of step 3f could let the population fall below that proportion.

Step 3f determines what a child inherits. Crossover retains the genes the parents share and distributes the genes they do not between the two children. The two children are therefore complementary, and each differs from both parents unless the random split happens to reproduce a parent, which is only likely when the parents differ in one or two genes. A parent pair that does not undergo crossover is carried forward unchanged into the mutation step. When the network term is active, and enough unselected genes carry a non-zero edge to the genes retained, the incoming genes are drawn in proportion to that edge weight with a probability of one half and uniformly otherwise. When it is not active, the draw is uniform. Both operators preserve exactly *k* selected genes.

Steps 3g and 3h separate two kinds of scoring. Rescoring the carried elites under the weights in force means that no chromosome is ranked under weights other than those of its own generation. Scoring the whole population under one fixed reference vector serves a different purpose: it makes chromosomes from different generations comparable, which the adaptive weights of Equation (3) do not.

The composite fitness function is given in Equation (1):

(1) *F*(*S*) = *w*_1_*′*(*t*) · *AUC*(*S*) + *w*_2_*′*(*t*) · *CI*(*S*) + *w*_3_*′* · *PPI*(*S*) − *w*_4_*′* · |*S*|/*N*

where each *w_j_’* = *w_j_*/(*w*_1_(*t*) + *w*_2_(*t*) + *w*_3_ + *w*_4_) so that the four weights sum to one at every generation. Here *w*_1_(*t*) and *w*_2_(*t*) are set by Equation (3), while *w*_3_ = 0.20 and *w*_4_ = 0.05 are constants. Because the normalizing sum changes with *w*_1_(*t*) and *w*_2_(*t*), the normalized values *w*_3_*′* and *w*_4_*′* vary even though *w*_3_ and *w*_4_ do not.

The final term penalizes panel size. Because both genetic operators preserve exactly *k* selected genes, |*S*| = *k* for every chromosome, so this term contributes a constant that does not affect the ranking within a generation. It is retained for consistency with the general form of the fitness function.

The AUC(S) is the mean of the five per-fold areas under the receiver operating characteristic (ROC) curve of an L2-penalized logistic regression at the scikit-learn default inverse regularization strength of 1.0. The model is fitted on the standardized expression of the selected genes on the same stratified partition in every generation. CI(S) is the concordance index [30] of a beta-weighted risk score. The score is the sum of the standardized expression values of the selected genes, each weighted by its univariate Cox coefficient, fitted on the discovery survival data with a ridge penalty of 0.1. An unweighted mean is not used because it would assign the same direction of effect to every gene and would cancel the contributions of genes whose associations with risk run in opposite directions. Two asymmetries should be noted. The first is that the diagnostic objective is cross-validated within the search, while the concordance objective is fitted and scored on the same discovery samples, so CI(S) is an apparent value and the more optimistic of the two. The second is that CI(S) and the concordance indices reported for the panel are not the same quantity. CI(S) weights the genes by univariate Cox coefficients and reaches about 0.61 during the search; the reported concordance is the linear predictor of a multivariable ridge Cox model and reaches about 0.70. The two are not comparable, and neither is offered as evidence of the other. When a value from the search and a value from the evaluation appear together, the protocol producing each is specified.

PPI(S), termed PPI coherence, is the mean STRING v11.0 interaction confidence over all pairs of selected genes, with pairs without a reported interaction contributing zero. It is called network coherence when referring to the property rather than its computation. Interaction scores were taken from the STRING v11.0 release files rather than the web interface so that the network is fixed and the analysis does not depend on the state of a remote service. Edges were retained at a combined score of 400 or above, STRING’s medium confidence level, and rescaled to the unit interval by division by 1000. The release files then provide 676,523 interactions among 14,493 human genes, of which the submatrix over the candidate pool is used. A pair below the threshold therefore contributes zero in the same way as a pair with no reported interaction.

Because the AUC and the concordance index need not improve together, *w*_1_(*t*) and *w*_2_(*t*) are adjusted at each generation by a Pearson-correlation-driven mechanism. The inter-objective correlation at generation *t* is given in Equation (2):

(2) *rho*(*t*) = Pearson(AUC(*e*),CI(*e*)) over the 2*W* most recently evaluated chromosomes *e*

with *W* = 8 so that the correlation is taken over the sixteen most recently scored chromosomes. The window is therefore expressed in evaluations rather than in generations. Because the population of forty exceeds 2*W*, the window lies inside a single generation and covers the sixteen offspring scored last, the elites being scored first. A reading over the *W* most recent generations is also admissible, and both were run to completion under otherwise identical settings; the two are reported side by side in Table S2A.

Intuitively, the mechanism treats disagreement and agreement between the two primary objectives differently: in a conflict state, where the diagnostic and prognostic scores move in opposite directions, the search shifts weight toward the prognostic objective so survival information is not crowded out; in an alignment state, where the two move together, confidence in both increases and both weights increase together. The exact rule is given in Equation (3):

(3) $$\left(w_{1}\left(t\right),\;w_{2}\left(t\right)\right)=\left\{\begin{array}{ccc} \left(0.30,\;0.50\right) & \mathrm{if}\; & \mathrm{rho}\left(t\right)\;<\;-\mathrm{rho}\_\mathrm{thresh}\\ \left(0.40\;+\;0.08\;\mathrm{rho}\left(t\right),\;0.40\;+\;0.04\;\mathrm{rho}\left(t\right)\right) & \mathrm{if}\; & \mathrm{rho}\left(t\right)\;>\;+\mathrm{rho}\_\mathrm{thresh}\\ \left(0.40,\;0.40\right) & \mathrm{otherwise} & \end{array}\right.$$

In the alignment state, the diagnostic weight rises at twice the rate of the prognostic weight, reaching 0.48 and 0.44 before normalization at *rho* = 1.

The branch that operates most of the time is the neutral one, in which the mechanism leaves both weights where they start. Across the ten reported runs of eighty generations, the neutral state holds for 534 of the 800 generation-events, while the alignment state holds for 198 and the conflict state for 68 (Table S2A). The dominant adaptive behavior is therefore the alignment branch raising both weights together, not the conflict branch redirecting the search towards concordance, and for two-thirds of the search, the weights are the fixed neutral pair. Under the alternative reading of *rho* discussed above, over the *W* most recent generations rather than the 2*W* most recent evaluations, the proportions change: 180 alignments, 278 conflict and 342 neutral. The two readings therefore differ not only in how often the mechanism exits the neutral state but also in which branch dominates.

The threshold |*rho*| = 0.3 corresponds to a medium-sized inter-objective correlation, following Cohen’s [31] effect-size convention. Zhang et al. [32] set a conceptual precedent for using the correlation between objective histories to drive adaptive reweighting when they applied Pearson correlation within an adaptive variable-grouping multi-objective evolutionary algorithm (MOEA) framework. The application to objective-weight adjustment in a biomarker genetic algorithm is a design contribution of the BIMS-GA. Weighting evidence sources by their discriminative performance has been applied to genomic–clinical fusion for survival prediction [33], whereas the BIMS-GA weights objectives by their mutual correlation during the search. The mechanism sets the two weights at every generation without requiring a predefined Pareto front, though at most generations, it sets them to the neutral pair and returns a single solution rather than a set from which one must be chosen after the search.

The values fixed above were examined over a grid rather than at a single point. The network weight *w*_3_ was varied over 0.05, 0.10, 0.15 and 0.20, with ten independent runs at each value (Table S3). How *w*_3_ was settled affects two of the results. A criterion written before the runs, maximizing the mean of the three objectives after min–max normalization, selects *w*_3_ = 0.05. The value used throughout was 0.20. It was chosen because the study reports selection stability and network topology as outcomes in their own right, and selection stability and network coherence both fall sharply at lower values (Table S3). The choice is therefore made according to the criteria reported in this study rather than the pre-stated rule, and the stability result is conditional on it. The correlation window *W* was varied over 5, 8, 10 and 15 and the threshold *rho_thresh* was varied over 0.2, 0.3 and 0.5 in a full factorial of twelve settings (Table S4A, Figure S1). Population size, mutation rate, crossover rate and panel size were each varied around their default value (Table S4B).

The search was repeated with ten random seeds: 42 + 7*k* for *k* = 0 to 9. A second block of ten, 112 + 7*k* for *k* = 0 to 9, was run for the stability analysis and is reported separately from the first. The selection frequency of a gene is the proportion of those runs in which it entered the final panel and therefore lies between 0 and 1.

Each run returns one panel of *k* genes, so ten runs return ten panels. The panel reported throughout is their consensus: the genes entering the final panel in at least six of the ten runs. This consensus does not depend on the exact threshold. Selection frequencies fall into two groups separated by an empty interval, twenty genes at 0.6 or above and nine at 0.3 or below, so every threshold between 0.35 and 0.60 returns the same twenty genes. Every quantity attributed to the BIMS-GA in Section 3 is computed from this consensus panel except for the ablation and stability analyses, which are computed run by run and then summarized.

### 2.3. Comparator Methods and Ablation Study

#### 2.3.1. Comparator Methods

Fourteen alternative feature selection methods, spanning six families, were implemented for comparison. The filter family comprised the ANOVA F-test, minimum redundancy maximum relevance [34], and LASSO [35], and the wrapper family was represented by SVM-RFE [36]. Two single-objective metaheuristics optimize the cross-validated AUC alone: binary particle swarm optimization (PSO) [37] and the binary grey wolf optimizer (GWO) [38]. Three Pareto-based multi-objective methods run with the same three objectives as the BIMS-GA: NSGA-II [10], NSGA-III [39], and MOEA/D [40]. The remaining five are two machine-learning importance methods, random forest impurity importance and permutation importance from a multilayer perceptron (MLP), and three deep-learning methods: LassoNet [41], DeepPINK [42] and an attention-based selector. Every method selects exactly twenty genes from the same 300-gene candidate pool and is scored under identical models, so the comparison is matched for panel size and for the evaluation procedure. Table S4C reports the settings for each as implemented.

Most of these methods do not return a fixed number of features on their own, so the table also reports, for each, the rule reducing a ranking or a variable-length solution to exactly twenty genes. The rule used is included in the comparison rather than considered an implementation detail, which is why it is stated per method rather than described once.

The three Pareto-based methods share one offspring operator. Two parents are drawn uniformly at random, and the child is a random twenty-gene subset of the union of their panels. With a probability of 0.15, one of its genes is replaced by a gene drawn from outside. These three methods share a step not required by the others: they return a front rather than a single solution, so one solution must be chosen once the search is completed. The non-dominated solution closest to the ideal point in a normalized objective space, commonly termed the knee point, is taken for comparison. Information from the completed search is used in this step and is therefore part of the procedure being compared rather than a post-processing convenience.

The knockoffs used in DeepPINK are permutations of the observed rows, which preserve each gene’s marginal distribution but not the covariance between one gene and another gene’s knockoff. The DeepPINK selection is used as a ranking, and no false discovery rate is claimed from it.

Several of these implementation choices could be different, as seven of them plausibly affect a comparator’s performance. For the two metaheuristic algorithms, these are the deterministic reduction to twenty genes and a budget of twenty individuals over sixty iterations rather than the forty over eighty generations used elsewhere. For the Pareto methods, they are the decision to use random rather than tournament mating, the absence of a niche count in NSGA-III, and the resampled weight vectors in MOEA/D. The remaining two are permutation knockoffs in DeepPINK and permutation importance computed on the data the multilayer perceptron was fitted to. In every case, the expected direction of the effect is to lower that comparator’s score. The differences between the BIMS-GA and these methods are therefore upper bounds on the advantage of the proposed method.

#### 2.3.2. Ablation Study Design

Three ablation variants were evaluated alongside the full model, each under the same protocol and using the same ten seeds. BIMS-GA-NCI removes the concordance term from Equation (1), so no survival information enters the fitness function. As a result, the concordance history is constant, which leaves Equation (2) undefined and holds the weights in the neutral state throughout. BIMS-GA-NPPI removes the network term from Equation (1) and, with it, the STRING-guided branch of the mutation operator, which draws on the same interaction matrix and is inactive once that matrix no longer enters the search. This variant therefore measures the contribution of the network component as a whole rather than of the fitness term alone. BIMS-GA-FW disables the mechanism in Equation (3) and holds both *w*_1_ and *w*_2_ at 0.40 before normalization, with all three objectives still present in Equation (1). Contrasts between the full model and each variant are paired by seed.

### 2.4. Validation Framework

#### 2.4.1. Diagnostic Classification

Five classifiers were trained on the selected panel under five-fold stratified cross-validation: logistic regression, support vector machine, random forest, gradient boosting and a multilayer perceptron. Logistic regression is used for all subsequent analyses because it is the classifier under which the diagnostic objective is computed during the search. Discrimination is summarized by the pooled out-of-fold prediction, which yields a single area under the curve rather than an average of five and is the more conservative of the two estimates.

Screening on the full discovery cohort before cross-validation is a recognized source of optimistic bias [43]. A five-fold nested protocol was therefore applied. Within the training partition of each outer fold, and using only the samples in that partition, differential expression screening was repeated, a fresh 300-gene candidate pool was constructed, and the genetic algorithm was rerun from a seed derived from the fold index. The resulting panel was scored on the held-out fold, which contributed to none of these steps. The same protocol was applied to every comparator, so the nested comparison is matched. The difference between the full-dataset estimate and the nested estimate quantifies the optimism attributable to selection. The quantile transformation and the probe-to-gene collapse precede the fold split and are not repeated within it, so the nested estimate bounds the optimism attributable to selection rather than to the pipeline as a whole. Neither step uses the outcome, and both are applied once to the single matrix every method is scored on, so their contribution is common to all eighteen panels and cannot affect the comparison this section reports.

#### 2.4.2. Prognostic Assessment

Within each survival cohort, expression values for the panel genes were standardized, and a Cox proportional hazards model with a ridge penalty of 0.1 was fitted [44]; this is the same penalty used for the univariate coefficients inside the search. The linear predictor of that model served as the risk score. Genes without a matching probe on a given platform were omitted from that cohort’s model without replacement. Patients were dichotomized at the cohort median of the risk score, with the patient at the median assigned to the low-risk group. Kaplan–Meier curves [45] were estimated per group and compared by the log-rank test [46], and Harrell’s C-index was computed as a continuous measure of discrimination. Hazard ratios (HRs) are reported with 95% confidence intervals from the same model.

Fitting the coefficients inside the cohort that is then scored with them gives an apparent estimate, so two further estimates are reported beside it. In the first, the coefficients are fitted once on the discovery survival data and applied to each external cohort without refitting. In the second, they are refitted inside each training part of a five-fold split within the cohort and the held-out part is scored, a process that retains the platform-specific scaling but does not reuse the outcome. The three are set side by side in Table S2D, and the apparent estimate is retained only so that the effect of the change can be seen.

Because the candidate pool is already enriched for differentially expressed genes, a panel drawn from it at random may produce the same figures. Twenty genes were therefore drawn at random 1000 times from the candidate pool and 1000 times from the whole gene matrix, and each draw was performed using the same protocol. The resulting distributions are the reference against which the panel is reported (Table S5A for the apparent protocol and Table S5B for the cross-validated one).

Adjustment for clinical covariates was possible only in the discovery cohort, the sole cohort used here with pathological stage and tumor grade deposited alongside survival. In that cohort, the risk group was entered into three nested Cox models: alone, with stage, and with stage and grade. One patient carries grade as GX and is dropped by any model using grade, so all three are fitted on the 64 complete cases. The unadjusted estimate on all 65 is reported outside the ladder rather than as its first rung.

#### 2.4.3. External Diagnostic Validation

A logistic regression model was fitted on all discovery samples using the selected panel and applied without refitting to each external cohort. Expression was standardized within each cohort before the model was applied because probe intensities are not comparable across platforms on their original scale. Panel genes were matched to each cohort by gene symbol, with one previous HUGO Gene Nomenclature Committee (HGNC) symbol resolved to its current form, that is, *PLEC1* to *PLEC*; when that resolution produced two entries for the same gene, the first was retained. Genes absent from a cohort were omitted from that cohort’s model, and the number available in each cohort is reported with the external results. Discrimination was summarized by the AUC with a bootstrap confidence interval.

#### 2.4.4. Functional Enrichment Analysis

Gene Ontology Biological Process, Reactome, and Kyoto Encyclopedia of Genes and Genomes (KEGG) enrichment analyses were performed on the selected panel using the Enrichr gene set libraries [47], with the 300-gene candidate pool as the background rather than the whole transcriptome. Terms with a Benjamini–Hochberg adjusted *p*-value below 0.05 were treated as significant. The network induced on the panel by the STRING v11.0 interaction matrix was characterized according to PPI coherence, the number of retained interactions, mean degree, clustering coefficient, modularity, the number of isolated genes, and maximum betweenness centrality. The same characterization was applied to the panel returned by every comparator. Node centrality is represented by betweenness and functional modularity by the modularity of the induced subgraph. Pathway topology in the sense of a topology-aware enrichment method is not used here. A twenty-gene panel is too small for the pathway perturbation statistics such methods estimate. Enrichment is instead reported against the candidate pool, which asks what distinguishes the panel from the genes it was selected from.

#### 2.4.5. Experimental Setup

All analyses were implemented in Python 3.10 using NumPy 1.26.4, pandas 2.3.3, scikit-learn 1.7.2, SciPy 1.15.3, lifelines 0.30.0 and NetworkX 3.4.2. The three deep-learning comparators used PyTorch 2.5.1, and LassoNet used the lassonet package, version 0.0.20. Those two versions matter more than the others for a specific reason. The epochs, learning rate and path density of the LassoNet regularization path are defaults of that package rather than the settings chosen in this study. The settings reported above therefore determine the method only in combination with the version that supplied them. Enrichment used the Enrichr gene set libraries, and interaction data were taken from the STRING v11.0 release files [14]. Experiments were run on an Intel Core i7-4770 processor (3.40 GHz, four cores, 16 GB RAM) using Windows 10 without GPU acceleration. A base random seed of 42 was fixed throughout. Analyses requiring repetition used seeds 42 + 7*k* for *k* = 0 to 9; nested procedures used 42 plus the fold index. Asymptotic complexity, measured runtime, peak memory and empirical scaling in population size and generation count are reported in Table S6A–D.

### 2.5. Evaluation Metrics

The diagnostic performance of each classifier was summarized using six measures: the area under the receiver operating characteristic curve, accuracy, sensitivity, specificity, F1-score, and the Matthews correlation coefficient [48]. The Matthews correlation coefficient was included because it provides a balanced summary when class sizes differ. Probability quality was assessed by the Brier score, by the expected calibration error (ECE) over ten equal-width bins, and by the Hosmer–Lemeshow test over five equal-count bins. Clinical usefulness was assessed by decision curve analysis, in which the net benefit at a threshold probability *pt* is *TP*/*n* − (*FP*/*n*) (*pt*/(1 − *pt*)), compared with the strategies of treating every patient and of treating none.

Prognostic discrimination was quantified by Harrell’s C-index [30], where 0.5 indicates no discrimination and 1.0 indicates perfect discrimination. Biological network coherence was quantified by PPI coherence, and the number of gene pairs carrying a non-zero score is reported alongside it. The mean alone does not distinguish a panel with several weak interactions from one with few strong interactions.

Selection stability was measured across the ten runs of each stochastic method using the mean pairwise Jaccard index and by the Kuncheva index, which corrects for the overlap expected by chance when drawing twenty genes from 300 [49]. A deterministic method returns an identical panel under a fixed seed, so its variability was instead assessed across ten bootstrap resamples of the discovery cohort, each requiring a complete rerun of the selection procedure. The two protocols are reported separately and are not pooled.

Pairwise AUC comparisons were performed using the DeLong non-parametric test [50] with a two-sided threshold of 0.05. Confidence intervals for the AUC and the C-index were obtained from 2000 bootstrap resamples. The same procedure was applied to accuracy, sensitivity, specificity, F1, and the Matthews correlation coefficient so that no measure in the classifier comparison is a bare point estimate.

PPI coherence was determined according to the panel and a fixed interaction matrix and therefore carries no interval. The same applies to the network topology measures derived from it—retained interactions, mean degree, clustering coefficient, modularity, isolated genes and maximum betweenness—and to Cohen’s *d*, which is a point effect size. The selection-stability indices and the nested cross-validation estimates are summarized by a mean and a standard deviation over their runs or folds rather than by an interval. The stability indices carry no interval because the 45 pairwise values within a method share panels and are not independent; the nested estimates carry none because five folds do not support one. Those quantities, with the enrichment *p*-values and the selection frequencies, are reported without an interval. Every estimate obtained on the discovery or external cohorts by resampling—the areas under the curve, the C-indices and the classifier operating characteristics—carries a 95% bootstrap interval. The hazard ratios carry the interval of the Cox model that produced them.

Paired contrasts between the BIMS-GA and its ablation variants were performed using the Wilcoxon signed-rank test over the ten seeds. With ten paired observations, the smallest *p* that the test can return is 2/2^10^ = 0.002, which is therefore the floor for every ablation contrast, however large the difference. Only the permutation tests reported below, which draw on 200,000 relabelings rather than on ten pairs, can return smaller values. Stability was contrasted between methods by permuting the run labels 200,000 times, with the *p*-value being the count reaching or exceeding the observed statistic plus one divided by the number of permutations plus one. A rank test over the pairwise index values was not used as the 45 pairwise indices within a method share panels and are not independent, and a test that treats them as independent observations overstates its own precision. Effect sizes for the diagnostic comparison are reported as Cohen’s *d* values computed for each panel between the out-of-fold scores of the tumor and non-tumor samples.

## 3. Results

### 3.1. Algorithm Behavior and Ablation Analysis

Every value reported below is a mean over the ten runs. Best fitness rose over the eighty generations (Figure S2), and the three objectives changed differently. The diagnostic objective changed little, and the concordance objective ended close to where it began. Network coherence accounted for almost all of the gain, rising from 0.0354 to 0.4558. Substituting the three objective means into Equation (1) under the reference weights returns the reported fitness at every generation, which fixes the three objectives and the composite to the same set of runs. The fitness plotted in Figure S2a is scored at the same reference weights and is reconstructed in the same way. Substituting instead the mean adaptive weights plotted in Figure S2c does not return it exactly, because the mean of a product is not the product of the means while runs occupy different branches of Equation (3). That substitution agrees to within 0.002 from generation 30 onward.

The inter-objective correlation left the neutral band in 266 of the 800 generation-events, a mean of 26.6 of 80 per run and between 23 and 34 in individual runs. Alignment was the more common departure, with 198 events against 68 in conflict, and the correlation ranged from −0.7016 to 0.7973. In the conflict state, the normalized weights are 0.2857 for the diagnostic objective and 0.4762 for the prognostic one. In the alignment state, the largest values observed were 0.4048 and 0.3770. Elsewhere, both stand at 0.3810.

The two readings differ in what the extra activity buys. The reading over generations estimates the correlation from half as many values and places the weights away from the neutral state far more often, for 458 of 800 generation-events against 266. Yet it reaches the same discrimination and concordance: a mean AUC of 0.9398 compared to 0.9416 and a mean C-index of 0.6176 compared to 0.6112. What separates them is reproducibility. The mean pairwise Jaccard index falls from 0.804 to 0.540. This is not a uniform loss: the standard deviation rises from 0.065 to 0.369 because two of the ten runs collapse away from the other eight, exactly as happens when the mechanism is removed altogether. Degrading the input to the mechanism and removing the mechanism entirely therefore produce the same failure mode from opposite directions. Neither observation is the result of a test, and both are reported as descriptions of the distributions rather than as demonstrations; nevertheless, they are more when considered together than either alone (Table S2A).

The six scalar values of Equation (3) were swept in the same way. Each branch’s pair was varied around its default over a grid of twenty-seven configurations, with three independent runs at each and more at the default and at one boundary cell, for ninety runs in all (Table S4A). Across the whole grid, the standard deviation between configurations is smaller than the root-mean-square standard deviation between seeds at a fixed configuration on every objective, by a factor of 1.6 for the AUC, 2.3 for concordance and 2.1 for network coherence (Table S4A, Panel C). Changing the six scalars anywhere in this grid therefore moves the result less than rerunning one configuration under a different seed.

The grid is not uniformly insensitive, and what varies follows how often each branch is in force. Network coherence varies most across the neutral grid, at a standard deviation of 0.0340 and least across the alignment grid at 0.0067, with the conflict grid between them at 0.0123. The neutral branch holds for 534 of 800 generation-events, the alignment branch for 198 and the conflict branch for 68 (Table S2A), so the branch in force two-thirds of the time is the one whose scalars matter most. That the conflict grid exceeds the alignment grid despite firing least often is a property of the grids rather than of the branches. The conflict grid moves both of its weights across their full range and the normalized network weight with them; the alignment grid varies only a small tilt around a fixed pair.

Three configurations lie at or beyond the constraints of Equation (3) and are included in the grid. At (0.4, 0.4), the conflict branch no longer raises *w*_2_ above *w*_1_, so the cell is the branch’s own boundary rather than a conflict configuration; its AUC, concordance, and coherence are not separable from the default (Table S4A, Panel E). A second cell reverses the conflict tilt outright at (0.5, 0.3), and a third removes the alignment tilt altogether, and both stay inside the ranges above.

The correlation window *W* and the threshold *rho_thresh* were swept over the same grid, and the result is of the same size. Across the twelve settings, the out-of-fold AUC varies by 0.0135 and the concordance index by 0.0076 (Table S4A, Figure S1). No parameter of the adaptive mechanism examined here—the six scalars of Equation (3), the window and the threshold—therefore moves any objective further than rerunning one setting under a different seed.

Each component was removed in turn, and the resulting panels were scored on the discovery cohort and on the eight external cohorts (Table 3 and Table S2E). Removing the concordance term changed nothing that could be detected. Removing the network term produced the largest effects in the study, and they act in opposite directions on the two sides. On the discovery cohort, the variant without the network term was better on both objectives in all ten seeds, by 0.0117 in AUC. On the external prognostic cohorts, the ordering reversed: the full model was higher by 0.0149 in concordance, again in all ten seeds. The external diagnostic difference also favored the full model but did not reach significance. Holding the weights fixed left discrimination unchanged; of its four contrasts, only the external prognostic difference reached a *p*-value below 0.05 (Table S2E).

Network coherence separated the variant that lacks the network term from the other three without ambiguity, and did not separate those three from one another. It was 0.4558 for the full model, 0.4655 without the concordance term, and 0.4048 with fixed weights; none of them were distinguishable from one another at ten seeds, compared with 0.0115 without the network term.

Runtime is close to linear in population size and in generation count at fitted slopes of 1.41 s per chromosome and 1.95 s per generation, and the search is dominated by the five-fold logistic regression of each fitness evaluation (Table S6C,D).

### 3.2. Comparison with Alternative Methods

**Figure 2:**
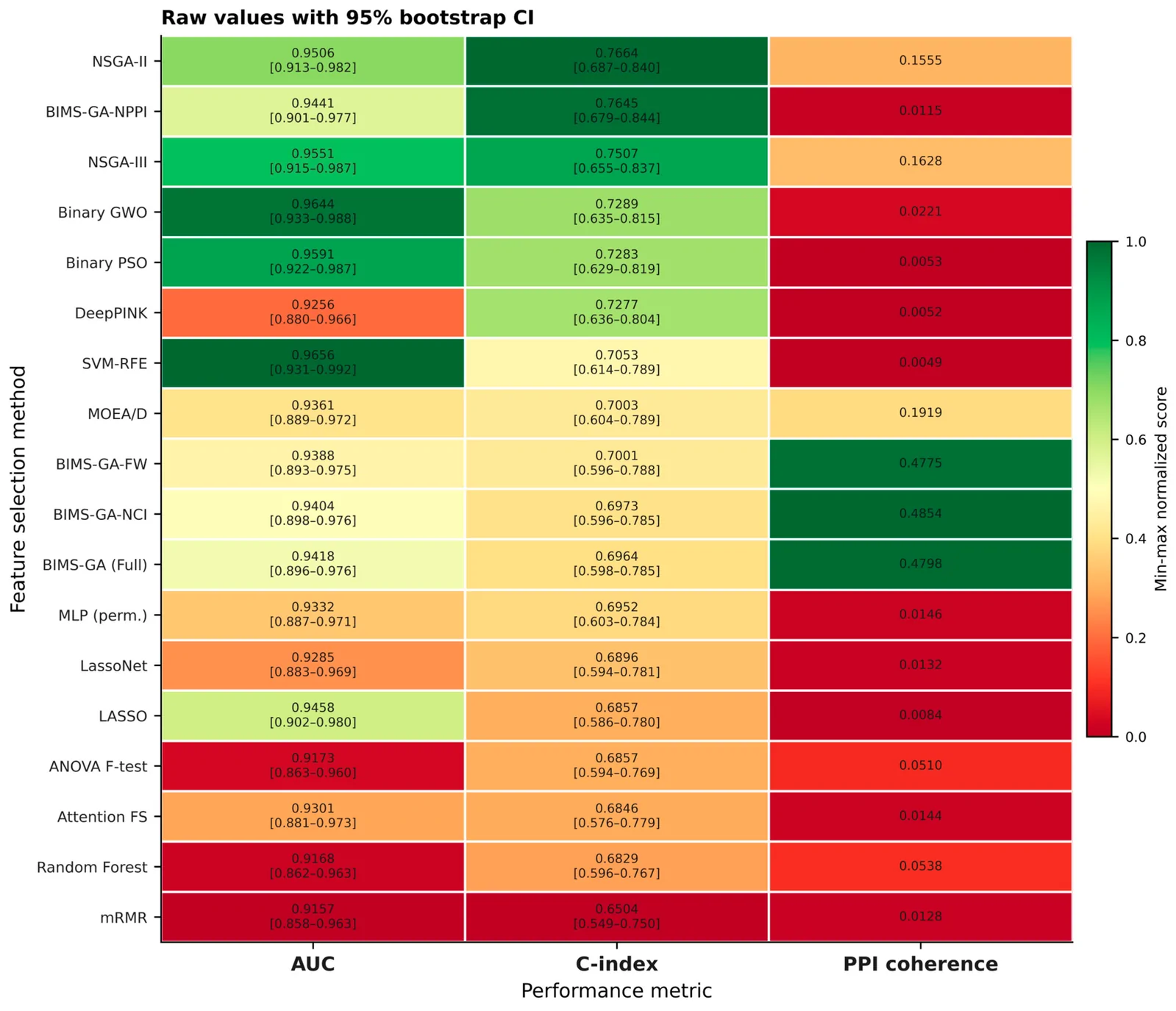
All eighteen panels: BIMS-GA, its three ablation variants, and fourteen alternative feature selection methods. Normalized heat map of discovery AUC, Cox concordance and PPI coherence, with the raw measurement printed in each cell and a 95% bootstrap interval on the two resampled quantities; PPI coherence carries none, being determined by the panel and a fixed interaction matrix. Each value is that of the single panel the method reports; for BIMS-GA that is the consensus panel, whose coherence of 0.4798 exceeds the ten-run mean of 0.4558 in Table 3.

Fourteen alternative methods were run on the same candidate pool and scored under the same models (Table 4, Figure 2). BIMS-GA performed competitively within the narrow margin of top performers on the discovery AUC: the full spread across all fifteen methods is narrower than any single method’s own bootstrap interval, so an ordering read from this column would separate methods that the data cannot distinguish. Every bootstrap interval overlaps that of the BIMS-GA, and the two DeLong comparisons that reached nominal significance before correction did not survive correction for multiple comparisons (Table S5E). Concordance shows the same pattern, with every interval again overlapping. Neither discrimination nor concordance on this cohort separates these methods.

Scoring every method over ten runs rather than one changes the ordering markedly: SVM-RFE falls from first to tenth, and the BIMS-GA remains seventh. The two orderings are not comparable because the ten runs are reseeded searches for the nine stochastic methods and bootstrap resamples for the five deterministic ones, and a panel chosen from resampled data scores lower for a structural reason. Table S6J’s means measure how much a method’s outcomes move, not how well it performs.

Network coherence separated the methods by two orders of magnitude. When each configuration was scored on the panel it reports, the three BIMS-GA configurations ranged from 0.4775 to 0.4854, with the reported panel among them at 0.4798. For the full model, this is the consensus panel; for BIMS-GA-NCI and BIMS-GA-FW, it is the mean over the panels each reports (Table S6F). No comparator came within a factor of two: the highest among the fourteen was MOEA/D at 0.1919 and the lowest SVM-RFE at 0.0049. This is the one axis out of six on which the fifteen panels separate at all. These coherence values are not those given for the same three variants in Section 3.1, which are means over ten runs (0.4048 to 0.4655, Table 3). A consensus panel keeps the genes the runs agree on and, therefore, scores above the mean of the runs it is drawn from. The two estimators order the variants the same way and should not be compared across tables.

The three configurations are not separable from one another; this does not rest on the precision of the consensus values alone. The paired test over the ten seeds does not separate them either. It gives the full model against the variant without the concordance term at *p* = 0.322, with the full model higher on 4 of 10 seeds, and against the fixed-weight variant at *p* = 0.156 with 5 of 10 (Table S2E). Two estimators, one a single panel and the other a paired comparison across seeds, agree that the difference is not distinguishable. Which of them leads depends on the measure taken. Removing the concordance objective gives the highest coherence and the largest number of interactions, fixing the weights gives the highest clustering coefficient, and the full model gives the highest maximum betweenness; all three leave no gene isolated (Table S6I). That removing the concordance objective increases coherence slightly is consistent with the ablation above, where the same variant trades external concordance for fit.

Out-of-fold ROC and precision–recall curves for all eighteen panels under logistic regression are shown in Figure S3. Areas ranged from 0.9157 to 0.9656, and average precision from 0.9104 to 0.9727, and the curves overlap across most of their length. The area a single curve requires is that of the pooled probability vector. Figure 2 reports the means of the per-panel areas. An area is fixed by the ranking of the scores rather than by their values, so the two are different operations with no fixed ordering between them. They differ only for the three ablation variants, each averaged over the panels it reports (Table S6F), and the pooled value is the higher for two of the three. The full model is one consensus panel and reads 0.9418 under both; its agreement to four decimals with BIMS-GA-NCI is a coincidence, with the two curves diverging and their average precisions being 0.9498 and 0.9485.

### 3.3. Diagnostic Performance Under the Nested Protocol

**Figure 3:**
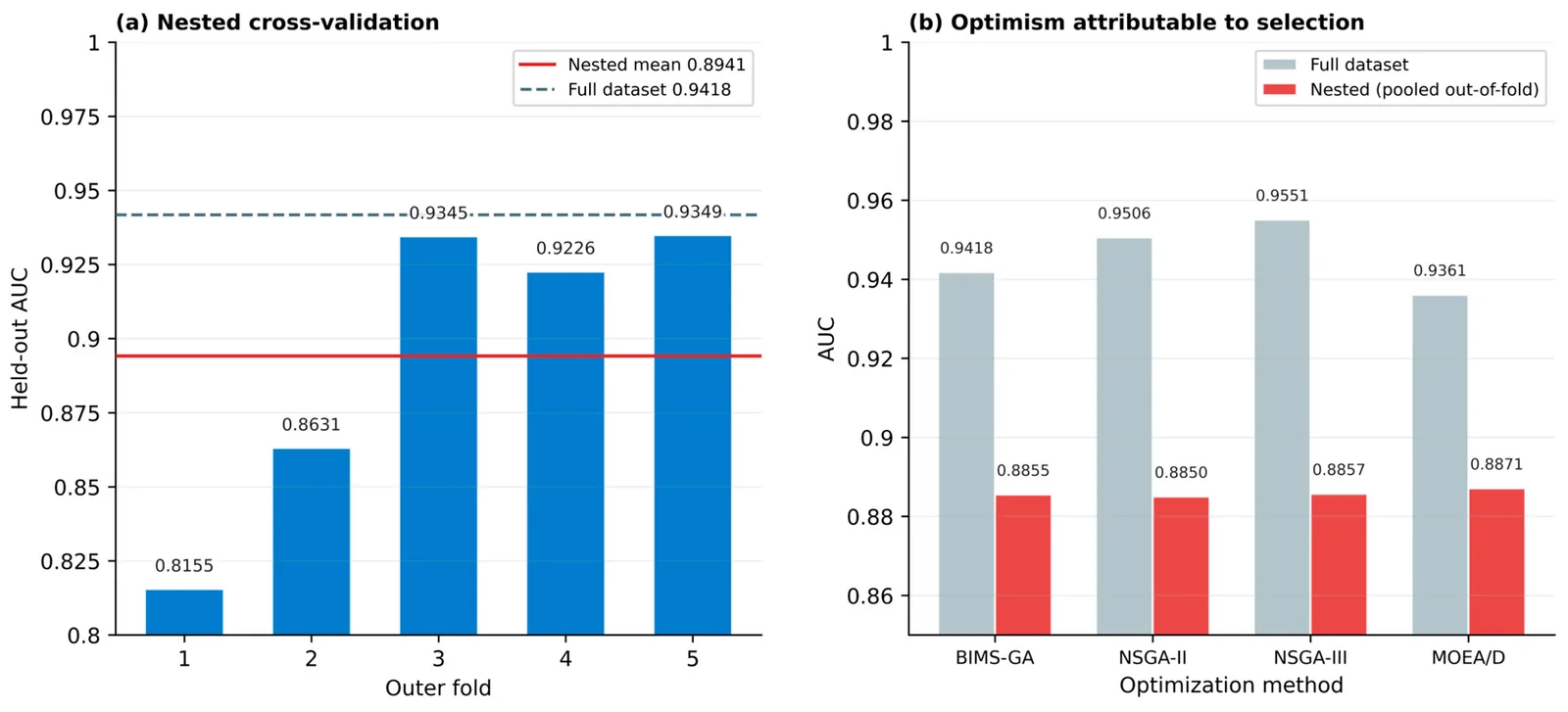
Diagnostic performance under a fully nested protocol. (**a**) Held-out AUC of each outer fold, with the mean of the five folds and the full-dataset estimate. (**b**) Full-dataset against the pooled out-of-fold estimates for BIMS-GA and the three Pareto-based methods.

Repeating the entire selection chain inside each outer training partition gave a mean held-out AUC of 0.8941 ± 0.0530 and a pooled out-of-fold AUC of 0.8855 against 0.9418 on the full dataset (Figure 3, Table S7A). The difference of 0.0563 is the optimism attributable to selection under the estimator; every comparator is scored with one search per outer fold. The same contrast cannot be drawn for prognostic concordance because the apparent value of 0.7036 and the search objective of 0.6112 are computed under different models. No nested estimate of the reported concordance is offered. The resubstitution contrast for the three ablation variants runs between 0.0945 and 0.1112 (Table S5D).

Optimism per fold runs from 0.0069 to 0.1263, a factor of eighteen, and the largest value belongs to the first fold alone; the folds disagree more than the methods do. The held-out AUC per fold ranged from 0.8155 to 0.9349, and the number of genes passing the screen within a training partition ranged from 898 to 2039 versus 1203 when the whole cohort was used (Table S7B). The panel found inside each outer fold shares between one and eighteen genes with the reported consensus, at Jaccard indices from 0.026 to 0.818 and a mean of 0.292 (Table S8A). Two folds recover most of the panel while three recover almost none of it, and the fold with the panel sharing the least with the reported one is also the fold with the lowest held-out AUC. With 130 samples, a five-fold nested estimate carries a standard deviation of the same order as the gaps it is being used to judge.

The consensus rule the reported panel uses cannot be evaluated on equal terms inside the folds, and the two figures below are third and fourth estimators rather than corrections of the two above. Applied inside the folds, its mean held-out AUC falls to 0.8620, with a standard deviation of 0.0972 and a pooled estimate of 0.8646 against 0.8941 and 0.8855 for the single search each comparator receives (Table S7C). Its apparent optimism of 0.0772 is therefore the optimism of that rule at one fifth of the sample, not of the rule as reported.

The same protocol applied to the three Pareto-based methods yielded pooled out-of-fold AUCs of 0.8850 for NSGA-II, 0.8857 for NSGA-III and 0.8871 for MOEA/D, with optimism of 0.0656, 0.0694 and 0.0490, respectively. The BIMS-GA is not the least optimistic of the four; MOEA/D is, at 0.0490 against 0.0563. Across all fifteen methods under the nested protocol, the pooled estimate ranged from 0.8765 to 0.9000, and the BIMS-GA ranked thirteenth. Under a protocol in which nothing is reused, the proposed method is among the weaker of the fifteen on discrimination.

Five classifiers were trained on the selected panel (Table 5). Logistic regression, the model under which the diagnostic objective is computed, gave the highest out-of-fold AUC, accuracy, and Matthews correlation coefficient. Three of the ten pairwise DeLong comparisons, all involving logistic regression, reached *p* below 0.05, and only the comparison with the support vector machine remained below the Bonferroni threshold for ten comparisons (Table S9A). Confusion counts at a threshold of 0.5 are given in Table S9B.

Calibration differed between classifiers more than discrimination did (Figure S4). The Hosmer–Lemeshow test did not reject calibration for logistic regression, random forest or the support vector machine, but it did reject calibration for the multilayer perceptron and gradient boosting, both at *p* < 0.001. The areas under the curve of these two differ from the others by less than 0.05. Brier scores and expected calibration errors are annotated in Figure S4. In decision curve analysis, logistic regression on the BIMS-GA panel gave a higher net benefit than either treating every patient or treating none. This held across the whole range of threshold probabilities examined, from 0.05 to 0.95, at a cohort prevalence of 0.531.

Calibration and the counts behind the sensitivity and negative predictive values for all eighteen panels are given in Table S6E. On that comparison, the reported panel is not well-calibrated relative to the others; its expected calibration error of 0.0781 places it fifteenth of the eighteen, with only mRMR, the ANOVA F-test and DeepPINK performing worse. That ranking is based on expected calibration error alone and does not transfer to the other measure, since the same panel is the tenth of eighteen on the Brier score. No Hosmer–Lemeshow result is available per panel: the tests reported above vary the classifier on one panel and not the panel under one classifier.

### 3.4. External Validation

A logistic regression fitted on the 130 discovery samples and applied without refitting reached AUCs from 0.8295 in GSE71729 to 0.9987 in GSE62165 across the four external diagnostic cohorts (Table 6). The GSE62165 figure should be interpreted considering that cohort’s composition: it contains 118 tumor samples and 13 non-tumor samples, so the estimate rests on thirteen negatives. Its bootstrap interval (0.9934–1.0000) is narrow, but with so few negatives and an estimate at the ceiling of the scale, it probably understates the uncertainty. All twenty panel genes had a matching probe in every one of the four cohorts. The four cohorts comprise 446 samples on three platforms other than the discovery array. This protocol does not reuse the outcome: the classifier is fitted once on the discovery samples and applied unchanged.

The null applied to the survival analysis also applies here. Against twenty genes drawn at random from the whole gene matrix, the panel is clearly better, at a mean external AUC of 0.9188 compared to 0.8360, with an empirical *p*-value of 0.011. However, it is not better against twenty genes drawn at random from the candidate pool; the null mean is 0.9231, and the panel sits at the 33rd percentile, at an empirical *p* of 0.671 (Table S5C). Once a gene has passed the differential expression screen, which of the 300 survivors enter the panel does not measurably change how the panel transfers; the transfer advantage therefore reflects the screen rather than the selection.

This result identifies where each part of the method contributes, and the same draws show what the candidate pool does and does not supply. The pool supplies the discriminative and prognostic signals since random draws from it match the panel on both axes. It does not provide the network structure: the mean coherence of a thousand such draws is 0.0129 compared to 0.4798 for the panel, and the largest single draw reached 0.0612, so no draw came within a factor of seven (Table S5F). Whatever produces the coherence happens during selection rather than during screening. That it reflects the network’s structure and not merely the strength of the prior is shown by the rewiring control. Rewiring STRING while preserving every node’s degree leaves discrimination intact, at an AUC of 0.9517 against 0.9416, while the between-run Jaccard index falls from 0.804 to 0.321 (Table S8C).

**Figure 4:**
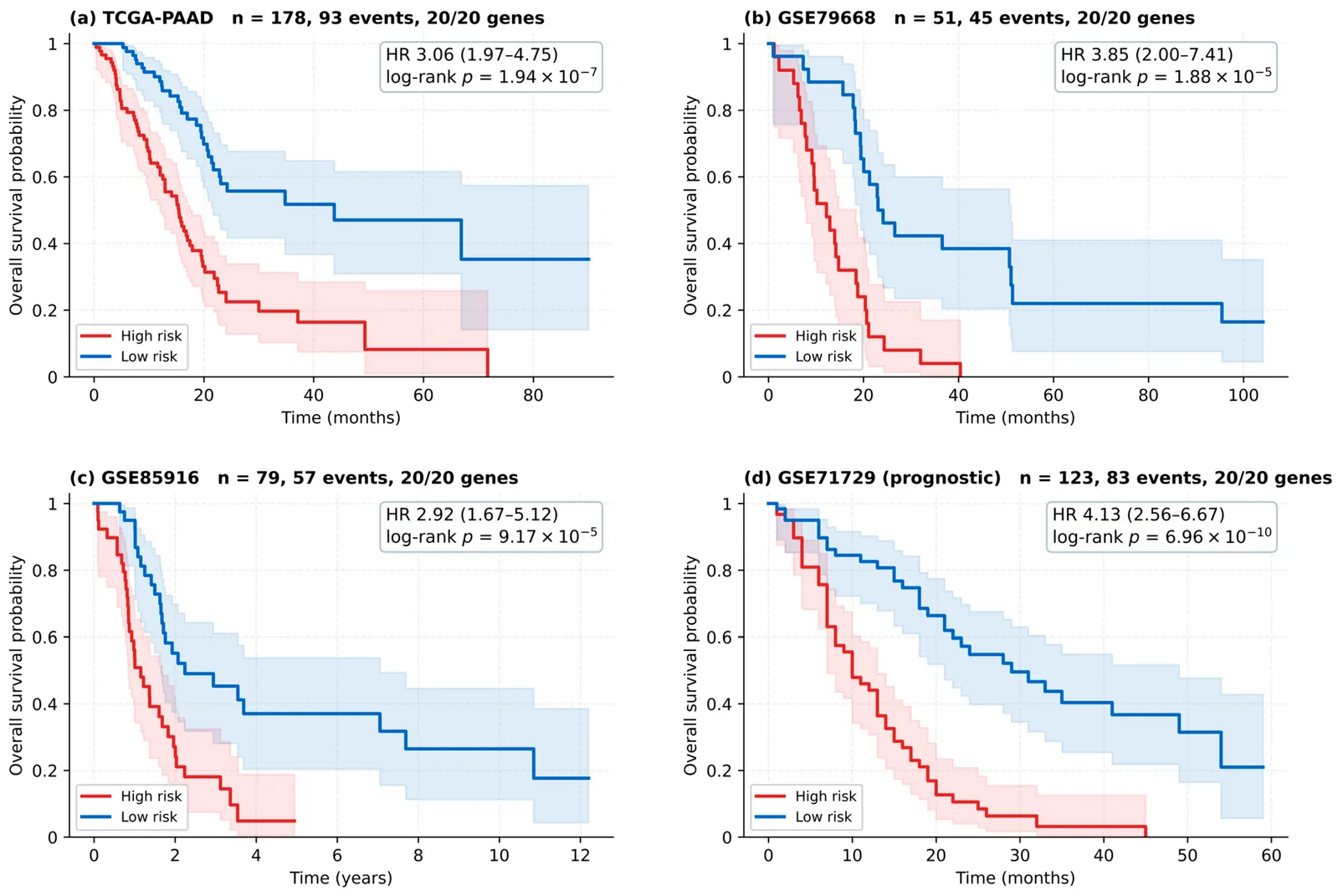
The panel stratifies overall survival in four independent cohorts, none of which contributed to selection, under the apparent protocol in which the coefficients are fitted inside the cohort that is then scored. Table S2D gives the two protocols that do not reuse the outcome. (**a**) TCGA-PAAD; (**b**) GSE79668; (**c**) GSE85916; and (**d**) GSE71729. GSE85916 is on a scale of years, while the other three are in months. The patient at the median joins the low-risk group, so the two groups differ by one when the cohort size is odd. All twenty panel genes are measurable in each of the four cohorts.

The prognostic result requires the protocol to be stated with it, because the three protocols do not agree. Under the apparent protocol, the coefficients are fitted inside the cohort and then used to score it. On that protocol, the panel separated the two risk groups in every external cohort, with hazard ratios from 2.92 to 4.13 and log-rank *p* below 10^−4^ in each (Figure 4, Table 6). These four cohorts comprise 431 patients and 278 events across four platforms, including two RNA-sequencing series.

That protocol does not distinguish panels. Under it, 994 of 1000 random twenty-gene draws from the candidate pool are significant by log-rank in all four cohorts at once, and the panel sits at the 69.8th percentile of their C-index distribution. Draws from the whole gene matrix are significant everywhere in 95.7% of the 851 that could be scored in every cohort (Table S5A).

Under the two protocols that do not reuse the outcome, the figures decrease (Table S2D). With the coefficients fitted once on the discovery data and transferred, the hazard ratio ranges from 1.02 to 2.17 and reaches significance in two of the four cohorts. With the coefficients refitted within each cohort under five-fold cross-validation, it ranges from 1.20 to 1.83 and reaches significance in one. Against the same null under the cross-validated protocol, the panel is not distinguishable from random draws from the candidate pool (empirical *p* = 0.34) and is only marginally above draws from the whole gene matrix (*p* = 0.07). This null rests on 200 and 165 draws rather than a thousand, so both figures are coarse (Table S5B).

What the panel carries in these cohorts is therefore the prognostic information already present in the differential expression screen that produced the candidate pool. Selection among those 300 genes does not add to it by an amount that this design can measure, and the hazard ratios of the apparent protocol should not be read as evidence that it does.

In the discovery cohort, the risk group remained associated with survival in all three nested models fitted to the 64 complete cases: alone, with stage, and with stage and grade (hazard ratios 2.84–3.33; 3.47 on all 65 patients). Neither stage nor grade was associated with survival after adjustment for the risk group. These are adjusted effects; neither covariate is fitted alone (Table S2B). The adjustment could not be repeated in the external cohorts as none deposits the covariates.

Carrying all eighteen panels to the same eight cohorts under the same models gave a mean external diagnostic AUC between 0.8693 and 0.9463 and a mean external prognostic C-index between 0.6886 and 0.7173 (Table S6F). The BIMS-GA reached 0.9188 and 0.7090, ninth of the eighteen panels on the combined mean and seventh of the fifteen methods once the ablation variants are set aside. Coverage of the panel genes on the external platforms ranged from 0.8187 to 1.0000 across the eighteen panels, and the three configurations that retain the network term are the only panels measurable in full in all eight cohorts (Table S6G).

### 3.5. Selection Stability

Across ten independent runs, the mean pairwise Jaccard index between the BIMS-GA panels was 0.804 ± 0.065 and 0.882 ± 0.043 for the Kuncheva index. Twenty-nine distinct genes entered a panel at least once, fourteen entered every panel, and twenty entered at least six of the ten (Table S10A).

Every other method was subjected to the same measurement, performed under seeds where the method is stochastic and under bootstrap resamples where it is deterministic (Table S6H). The highest value among the fourteen comparators was 0.566 for random forest, and the lowest was 0.048 for the multilayer perceptron under permutation importance. The BIMS-GA value is 1.4 times the highest of the fourteen, a difference of 0.238 at a Cohen’s *d* of 3.53, which one of 200,000 run-label assignments reached (*p* = 1.0 × 10^−5^, Table S10C). The permutation count was raised from 20,000 to 200,000 because at the lower count, the tail of this test was unresolved. The same contrast returned no permutation at or beyond the observed difference on one random stream and a single permutation on another, so the value reported was a bound rather than an estimate. Where the count is still zero at 200,000, the estimate takes its smallest possible value, 1/200,001 = 5.0 × 10^−6^; the table gives every value with its count. Two random twenty-gene draws from the same 300-gene pool have an expected Jaccard index of 0.035, which is the baseline against which all of these values should be read.

Two controls bound what the stability depends on. Under bootstrap resampling of the discovery cohort rather than reseeding, the mean pairwise Jaccard index of the proposed method falls from 0.804 under reseeding (Table S10A) to 0.209 and further to 0.036 when the candidate pool is rebuilt inside each resample (Table S8B). That protocol is the only one in which the five deterministic methods can be measured at all, and under it, their values range from 0.1053 to 0.2167. The Jaccard index value of the proposed method lies inside this range, ranking second. Stability does not separate the methods under either protocol. The figure reported here is therefore conditional on a fixed cohort and a fixed pool, as it measures the reproducibility of the search, not of the whole pipeline under new data.

The second control replaces the network rather than the data, and the three conditions form an ordering. Under STRING as released, the mean pairwise Jaccard index is 0.804 at a network coherence of 0.4558. Under a degree-preserving rewiring, it falls to 0.321 at 0.1606, and under a weight-shuffled network it drops to 0.218 at 0.0058 (Table S8C). Both controls move in the same direction, and the weaker prior gives the weaker result. The stability depends on the specific interactions STRING records rather than on the presence of a strong prior alone—a rewiring with an identical degree distribution would supply that and does not reproduce it.

Two further counts describe the same difference without a threshold. Fourteen of the twenty genes recur in every one of the BIMS-GA’s ten runs, compared to a range from zero to seven across the fourteen comparators. Ten runs of twenty genes drawn at random from the pool would touch 150 distinct genes in expectation, compared to 29 for the BIMS-GA, 40 for random forest, 70 to 89 for the three Pareto methods and 138 for the multilayer perceptron. Neither count is independent of the Jaccard index, since a method that visits few distinct genes must have high pairwise overlap almost by design, and the two rank the methods almost identically.

At the six-of-ten threshold that defines the reported panel, the separation is narrower, but the ordering remains the same. The BIMS-GA reaches twenty genes. Among the nine comparators run under the seed protocol, random forest reaches seventeen, DeepPINK and NSGA-III eleven, NSGA-II nine, and attention-based selection six, and the remaining four reach three or fewer; the multilayer perceptron reaches none. The five deterministic methods, counted over bootstrap resamples, reach between two and eight. Random forest is therefore the closest comparator on this count as it is on the Jaccard index, and the margin over it is the narrowest one throughout. The full distribution of selection frequencies for the BIMS-GA and the nine comparators run under the seed protocol is given in Table S10D, and the counts for the five deterministic methods are given in its note.

The 45 pairwise indices within a method are not independent, since each panel enters nine of them, so contrasts between methods are tested by permuting the run labels rather than by a rank test over the pairwise values.

Among the ablation variants, removing the concordance term left stability essentially unchanged at 0.771 (*p* = 0.286). Removing the network term reduced it to 0.213, a difference of 0.591 at *p* = 1.5 × 10^−5^, with 2 of 200,000 permutations reaching it. Fixing the weights reduced it to 0.467, a difference of 0.337, which does not reach significance over ten runs (*p* = 0.053). Of the two components, only the network term has an effect on stability that the permutation test separates from chance, and its effect is the larger of the two.

The figures above are ten-seed estimates and do not replicate on a second block of ten. Repeating the search at ten further seeds gives a mean pairwise Jaccard index of 0.521 within that block, compared to 0.804 within the first and a Kuncheva index of 0.614 against 0.882. Pooled over all twenty runs, the two indices are 0.649 and 0.736 (Table S10E). The two indices fall by 0.283 and 0.268, so the instability is a property of the selection and not of the Jaccard measure, which Kuncheva corrects for chance overlap. The consequence for the comparison here is direct: the difference between the two blocks of the same method, 0.2828, is larger than the difference of 0.2385 between that method and the most stable comparator. Ten seeds do not estimate this quantity precisely enough to order methods by it.

The contrast against the fixed-weight variant does not survive the additional runs either. At twenty per arm, the permutation test over run labels returns *p* = 0.282 compared to 0.053 at ten per arm, and within the new block alone, the ordering reverses, with the fixed-weight variant reaching 0.6990 against 0.5213 for the full model. Two runs out of twenty fall below a leave-one-out agreement of 0.30 in each arm.

### 3.6. Panel Composition and Function

The panel comprises twenty genes (Table 7). Fourteen entered the final panel in all ten runs, three in nine, one in eight, and two in six. The panel is dominated by integrin subunits and laminin chains: *ITGA2*, *ITGA3*, *ITGA11*, *ITGB1*, *ITGB4* and *ITGB5*, together with *LAMA3*, *LAMB3* and *LAMC2*.

**Figure 5:**
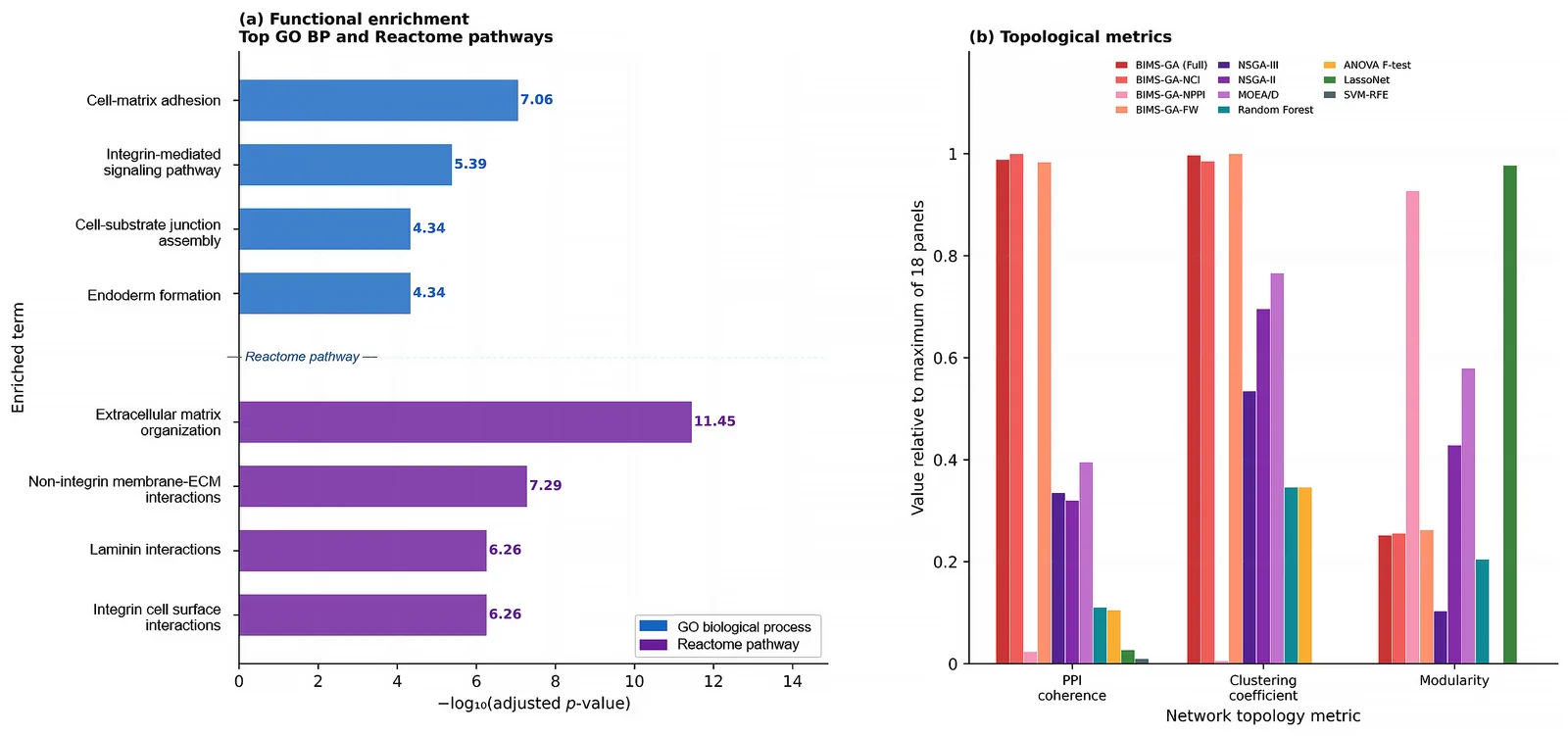
Functional and network characterization of the BIMS-GA feature space. (**a**) The four most strongly enriched Gene Ontology Biological Process terms and the four most strongly enriched Reactome pathways, tested against the 300-gene candidate pool as background. (**b**) PPI coherence, clustering coefficient and modularity for the four BIMS-GA configurations and seven comparators from five of the six families. Modularity and clustering are computed on whatever subgraph a panel induces, so a panel retaining almost no interactions can reach a high value on either; the interaction counts in Table S6I should be read alongside them.

Against the candidate pool as a background, twenty of the twenty genes appear in at least one significantly enriched term (Table S11). Sixteen appear in the eight Gene Ontology and Reactome terms shown in Figure 5, and the remaining four, *PLAU*, *THBS2*, *ACTN4* and *MET*, appear in other terms of Table S11. The strongest Reactome pathway was Extracellular Matrix Organization, which contained 14 of the panel genes at an adjusted *p*-value of 3.5 × 10^−12^. The strongest Gene Ontology term was Cell–matrix Adhesion, which contained 9 of the 13 genes carrying that annotation in the background, at an adjusted *p*-value of 8.7 × 10^−8^. In KEGG, ECM–receptor interaction contained 12 of the 12 genes carrying that annotation in the background, and Focal adhesion contained 13 of 15.

The subgraph produced on the panel by the STRING network retained 111 of the 190 possible pairs, with no isolated gene, a mean degree of 11.1, a clustering coefficient of 0.7203 and a modularity of 0.1228 (Table S6I). Figure 5 places these against ten other panels, each value scaled to the largest observed among all eighteen panels (Table S6I). Across the eighteen panels, the number of retained interactions ranged from 1 to 112. The three BIMS-GA configurations account for the top of that range and lie within one interaction of one another at 111.0, 111.7, and 112.0, so they are not separable on this measure either. The highest among the fourteen comparators was 47 for MOEA/D, and nine of the fourteen retained 5 or fewer. Isolated genes ranged from 0 for all three BIMS-GA configurations to 18 of 20 for SVM-RFE and DeepPINK.

Together, these are what is offered in place of a topology-aware enrichment analysis: the first asks which processes distinguish the panel from the pool it was drawn from, and the second asks how the panel sits within the interaction network.

## 4. Discussion

### 4.1. BIMS-GA Design and Multi-Objective Trade-Offs

The BIMS-GA is a scalarized search over fixed-size gene subsets. Three properties of the design follow from what the study set out to measure. The fitness function carries diagnostic accuracy, prognostic concordance, and network coherence at once because a panel selected on any one of them is not good on the others. The weights on the first two follow the observed correlation between them rather than being fixed in advance, so that the search does not spend effort separating objectives that happen to agree. The panel size is fixed at twenty, which makes every method in the comparison return an object of the same kind and removes panel size as a confounder. The advantages and disadvantages of each of these choices are the subject of this section.

The three ablation variants are the same search with one of those choices removed, and each returns a panel of its own. Removing the network term raises discovery discrimination slightly (Table 3) but collapses network coherence, so the panel it returns is a set of individually strong genes rather than a connected module. Removing the concordance term leaves both discrimination and coherence intact and gives up the survival axis. Holding the weights fixed leaves discrimination and network coherence as they were with the full model but lowers the agreement between runs. None of the three performs worse than the full model on discrimination, and the case for applying the full model rests on what the other three give up rather than on what they lose.

Fifteen size-matched panels selected from one candidate pool and scored under one protocol span a range in discovery AUC narrower than the width of a single bootstrap interval, and almost no pairwise differences are distinguishable from chance. Discrimination, on this problem, is close to being saturated: it can confirm that a panel works, but it cannot order the methods that produce panels. This reveals a limitation of ranking feature-selection methods by AUC alone. Selection stability cannot order them either, and not because the methods are alike. Ten runs do not estimate it precisely enough to try: the same method differs between two blocks of ten seeds by more than it differs from the most stable comparator. Network coherence can, and it does so completely. It spans two orders of magnitude across the fifteen methods, from 0.0049 to 0.4798, and no comparator reaches half the value of the proposed method. The ordering holds in every one of twenty runs across two independent seed blocks. Of the three axes on which these methods were compared, one separates them, and two do not, and separation on the one survives a second block of seeds.

This claim is made precisely because the second block also shows what it does not support. The mean coherence over ten runs does not shift, with 0.4558 in the first block against 0.4114 in the second, a difference of 0.0444 that a permutation test does not distinguish from chance at *p* = 0.150 (Table S10E). The ordering against the comparators holds in every one of the twenty runs. The weakest reaches 0.2310, compared with 0.1919 for the best single panel any comparator reports and 0.1500 for the best comparator mean over ten runs, so the separation is complete rather than average. The run-to-run spread, however, is wider than the first block suggested, with its standard deviation rising from 0.0234 to 0.0868, with the weakest run at 0.2310. In this context, coherence is therefore not described as stable; what is established is that its mean does not move, that its ordering is unbroken across twenty runs and that individual runs vary more than ten seeds indicated.

Venet et al. [58] argue that testing a panel against random gene sets of the same size should be routine, but it is not. When using this method to test published breast cancer signatures, they found that most were not more strongly associated with outcome than random sets. A signature reported without that comparison has therefore not been shown to carry information beyond the set it was drawn from. When reported against both backgrounds, the two answers differ. This is due to the screen approach rather than the search approach; what the candidate pool already guarantees, the selection cannot add to.

The difference between the two axes is one of scale rather than of kind. The seed-block shift is 0.0444 against a between-method gap of 0.3058 on coherence and 0.283 against a gap of 0.238 on stability. This represents fifteen per cent of the distance it must carry in the first case and more than the entirety of it in the second. On the consensus panel, the same asymmetry is visible without any test. The best comparator retains 47 of the 190 possible STRING interactions. Every one of the fourteen leaves between three and eighteen of its twenty genes without a single recorded interaction, whereas the panel reported here leaves none.

The ablation identifies what each component supplies, and one component behaves in a way that a single-cohort study would have misread. Removing the network term improves fit to the discovery cohort on both performance objectives in every one of ten seeds and reduces concordance in the external prognostic cohorts, again in every one of ten seeds. A term that costs accuracy where it is fitted and returns accuracy where it is not is behaving as a regularizer rather than as a performance objective. Its effect is not to make the panel better on the data the search sees; it is to constrain which of the many panels that fit that data equally well is returned. The price and the return are therefore reported separately, since either alone would misrepresent the term.

The concordance term is a different case. Removing it changes no measured quantity beyond the resolution of ten seeds on either the discovery cohort or the external ones. Two readings are available. The genes that separate tumor from non-tumor tissue in this disease may already carry the prognostic signal the term is meant to add, in which case the term is redundant here and might not be elsewhere. Alternatively, the concordance estimates available inside the search, from 65 patients and 49 events, may be too noisy to steer selection. The present design cannot distinguish these, and the term is retained rather than removed because its cost is a longer runtime rather than a worse panel.

The adaptive weighting mechanism acts on neither performance objective. Holding the weights fixed leaves discrimination unchanged, and the decrease in between-run agreement does not reach significance over ten runs (Table S10B). A structurally different weighting mechanism reaches the same qualitative conclusion elsewhere. An ensemble that couples each component’s weight to a view-specific power of its own cross-validated AUC found that replacing that weighting with uniform weights left discrimination materially unchanged. The diversity of the components rather than the allocation of weight among them drives performance [59].

The mean is the wrong summary here, and the ten runs show why: they have a graded distribution rather than a shifted one. Across the full model, the 45 pairwise indices lie between 0.667 and 0.905; across the fixed-weight variant, they lie between 0.026 and 0.905, with a standard deviation of 0.321 against 0.065. Seven runs cluster, one is degraded, and two share almost no genes with the rest. The seven that cluster reach a mean network coherence of 0.4576 against the full model’s ten-seed mean of 0.4558, a difference thirteen times smaller than the full model’s own variation across seeds.

The two that do not cluster collapse to 0.2477 (Table S8D). Those two score marginally higher than the seven on discrimination and concordance: 0.0008 higher in AUC and 0.0069 higher in the concordance index, each figure being the difference between the two group means within the same block of ten runs. Neither is tabulated, since the subgroup table carries coherence and overlap only.

This pattern shows what a failure-mode guard would look like rather than a quality improvement: a rare, unusable run made rarer rather than a typical run made better.

This pattern is presented as a description of the two distributions and not a demonstration. Two failures in ten against none in ten give a Fisher exact *p*-value of 0.474, and counting the degraded run as a third gives 0.211. Ten runs per arm cannot resolve a difference of that size; with no failures in one arm, five would be needed in the other before a two-sided test value fell below 0.05. Neither the difference in means nor the difference in failure rates is established here, and establishing either would require more runs than performed in this study.

The outcome of an independent comparison suggests the same. Estimating the inter-objective correlation over eight generations rather than sixteen evaluations degrades the input to the mechanism without removing it and produces the same result: unchanged discrimination and concordance. There is a decrease in the between-run Jaccard index driven by two of ten runs collapsing rather than by a uniform loss. Removing the mechanism and degrading its input pushes runs into the same tail. Neither comparison is a formal test, but the two independent experiments agree. A mechanism that changes which of several equally fitting panels is returned, without changing how well the returned panel fits, is worth having only if reproducibility itself is valued. Because a failure of this kind is invisible on the two axes normally used to judge a panel, a study reporting only discrimination and concordance would not detect it.

NSGA-III and NSGA-II both reach a higher discovery AUC and a higher concordance than the BIMS-GA, and neither difference is significant. On network coherence, the ordering is reversed by a factor of three, and that difference is not a matter of resolution. Under the nested protocol, all four estimates fall below their full-dataset values, and the BIMS-GA values decrease more than the MOEA/D values. Nothing in the nested comparison favors the proposed method; rather, the case for it rests on network coherence, the one axis on which the separation is large.

One comparison is more relevant to the conclusions than any other. The ANOVA F-test, a filter that costs a fraction of a second, transfers to the external cohorts better than any panel compared here and is outperformed on external prognostic concordance only by NSGA-III. On network coherence, the axis this study emphasizes, the ordering reverses by almost an order of magnitude. This contrast highlights the complementary value of network coherence: the case for a three-objective search rests on returning a connected, interpretable module, not on external transfer, and that case is made on its own terms.

The results can also be compared with a negative result reported in the literature. Staiger et al. [60] found that network-based composite classifiers did not outperform single-gene classifiers on breast cancer outcomes and that randomizing the network left their performance intact. The results of this study agree with that finding rather than contradict it; removing the network term increases the discovery AUC and collapses network coherence (Table 3), so the term costs discrimination and buys structure. However, this study’s claims relate not to accuracy but to interpretability. Their second finding does not transfer. The rewiring control described in Section 3.4 leaves discrimination intact and costs most of the between-run agreement, so what the network contributes here is the reproducibility of the selection, which the earlier work did not measure. The same distinction separates this study’s result from the literature on stability [15,61,62].

One difference between the two designs is structural rather than measured. A Pareto search returns a front from which a single solution must be chosen after the search, and the rule used to choose it draws on the completed search. A scalarized search with an adaptive weight returns one solution and no such choice arises. Whether that is preferable depends on whether a front is wanted; where it is not, it is a cost without a return. Maintaining a front costs time: the BIMS-GA completed in 201 s compared to a range of 273 to 288 s for the three Pareto-based methods, measured in the same invocation under identical instrumentation (Table S6B). No ratio is quoted because the cost of the timing instrumentation scales with the number of allocation events rather than with peak memory, and the searches compared differ in how often they allocate. The ordering is robust to that, but its magnitude is not. Peak memory is reported for the process rather than per method. The deep-learning frameworks are imported once and not released, so resident memory is a step function of the order in which the methods run rather than a property of any one of them (Table S6B).

### 4.2. Biological Significance of the Selected Panel

The panel is not a list of independently interesting genes: it is one coherent program. Six integrin subunits, three laminin chains and their assembly and remodeling partners describe the cell–matrix adhesion machinery, which is what the enrichment against the candidate pool recovers. The genes were drawn from the most strongly differentially expressed in the discovery series, so recovering known PDAC biology is expected rather than notable. What is not automatic is that the twenty genes form a connected module rather than twenty separately informative markers, which is the property the network objective was included to obtain.

The laminin-332 axis is the clearest instance and is included in the panel in its entirety. All three chains of the heterotrimer, *LAMA3*, *LAMB3*, and *LAMC2*, are selected in all ten runs, together with the alpha3beta1 integrin that binds it, present as *ITGA3* and *ITGB1*. *ITGB4*, *PLEC*, and *COL17A1* are also selected; they form the hemidesmosome that anchors the same adhesion from inside the cell. Overexpression of all three chains is associated with poor prognosis in univariate analysis, though none retained significance in a multivariate model of 96 patients [52]; cytoplasmic gamma2 expression was linked to hepatic metastasis two decades earlier [63]. The combination offers more than its individual parts, a signature of the three chains together predicting the outcome more strongly than each alone [64], and laminin-332 signaling through alpha3beta1 has been shown to drive invasion in this disease [54,65].

When considered together rather than one gene at a time, these members communicate not only that PDAC is present but also how it progresses. Detachment of the tumor cell from its basement membrane, degradation of the surrounding matrix, and invasion through it are one sequence, and the panel contains components of each step. Laminin-332 and its integrin receptors supply the adhesion itself. *COL17A1*, *PLEC*, and *ITGB4* form the hemidesmosomal anchor that has to be dismantled. *PLAU* and *MMP14* supply the pericellular proteolysis that opens the path, and *FN1*, *VCAN* and *THBS2* the matrix deposited in its place. The contribution of the panel to PDAC progression is therefore a contribution to one process rather than twenty; this is also the most economical reading of its prognostic behavior. The panel separates high- from low-risk groups in all four external cohorts under the apparent protocol. It is unlikely that twenty genes chosen for diagnostic separation are each independently strong survival markers. A tumor that has remodeled its matrix is further along the sequence above, and that is what the risk score appears to measure.

The dense desmoplastic stroma of PDAC itself is an extracellular matrix phenotype, so a panel dominated by matrix receptors is consistent with the tissue being profiled. The corollary is that bulk expression cannot separate a change within the neoplastic compartment from a change in the proportion of stroma, and the panel should be read as a tissue-level rather than a cell-level signature.

Several members carry independent evidence for this disease. High *LAMC2* was associated with poor overall survival on multivariate analysis in 114 patients and with resistance to gemcitabine [51], and *LAMC2* enters the panel in all ten runs. *PLEC* was identified as a PDAC biomarker by phage display and proteomic screening, a route with no methodological overlap with selection from expression data [55], and it too enters all ten runs. *THBS2* measured with *CA19-9* detects early PDAC in blood [56], although the same combination underperforms in prediagnostic samples drawn before clinical presentation [57]; *THBS2* enters in six of the ten runs. The second result is deliberately reported beside the first because a marker that performs at diagnosis but not before it is different from one that does both. The evidence behind the panel is not uniform. No PDAC-specific result is cited here for *ITGA11*, *ITGB5*, *ACTN4*, *CTTN*, *SDC1*, or *MET*, and *ITGB4*, *COL17A1*, *PLAU*, and *MMP14* are described above by their function rather than by a PDAC-specific result. These ten are present on the strength of the search and of the enrichment in Table S11 and are reported as members of a module rather than as independently supported markers. *MET* is the one of the ten that the paragraphs above do not otherwise address. It enters the panel in nine of the ten runs and is recovered by the same focal adhesion and PI3K-Akt signaling terms as the integrin subunits beside it, which represents the whole of what this study establishes.

On therapeutic strategies, the panel is more useful for the process it identifies than for any gene it nominates. Three of its members, *ITGA2*, *LAMB3* and *LAMC2*, have been proposed as potential therapeutic targets in this disease from an independent analysis [53]. Two published results attach the panel to drug response rather than to drug targeting: laminin-332 expression predicts chemotherapy response as well as clinical outcome [64]. *LAMC2* modulates gemcitabine resistance through the epithelial-to-mesenchymal transition and ATP-binding cassette transporters [51]. Both are stratification hypotheses. Set against them, this study nominates no target of its own: nothing in the design tests intervention; the panel was selected for diagnostic separation, and an association between expression and outcome is not evidence that changing the expression changes the outcome. The therapeutic implication these data support is that the panel points at cell–matrix adhesion and matrix remodeling as processes with a plausible rationale for intervention. The individual genes are candidates for stratification before they are candidates for targeting.

Two properties determine whether a diagnostic panel is usable, and neither is the area under the curve. The first is whether the probabilities it produces are trustworthy: under logistic regression they are, and under two of the five classifiers examined they are not, at areas under the curve that differ by less than 0.05. A classifier chosen based on discrimination alone can therefore return miscalibrated probabilities without any indication in the reported metric—a dissociation also reported across breast, prostate, pancreatic and thyroid classifiers [66]. The second is whether acting on the panel is more effective than the default strategies. According to the decision curve across the whole range of threshold probabilities examined, it is.

The positive and negative likelihood ratios of the panel are 7.7 and 0.13. Unlike the predictive values, these do not depend on prevalence. At the tumor proportion of the discovery series, the positive predictive value is 0.90. At a prevalence of 10%, the same likelihood ratios give 0.46, and at 5%, they give 0.29, while the negative predictive value rises from 0.87 to 0.99. This is the profile of a test suited to excluding disease rather than confirming it. Likelihood ratios are the form in which the result should be carried into any screening context because the discovery series is enriched for tumors by design, and no prevalence encountered in practice resembles it.

One limitation follows from the biology rather than from the protocol. A panel dominated by extracellular matrix and adhesion genes is a description of the basal-like program. Moffitt et al. [67] defined that subtype as carrying the worse outcome, and it appears as the squamous subtype in the classification of Bailey et al. [68]. Other schemes categorize the disease differently [69,70]. This study did not stratify by subtype, and the discovery series was not assembled to balance them. A panel of matrix and adhesion genes may therefore report subtype composition as much as the presence of disease, and these data cannot separate the two. The diagnostic result is correct as far as the measurements go and incomplete as a biological claim; the experiment that would separate them is a subtype-stratified analysis, which was not performed in this study.

### 4.3. Limitations and Future Directions

Selection rests on a single series of 130 samples on one platform, and both objectives are computed on it, so the reported panel reflects that series. The size of that series is a limitation in its own right. The held-out AUC varies from 0.8155 to 0.9349 across the five outer folds, and per-fold optimism spans a factor of eighteen. The bootstrap interval on a single discovery estimate is wider than the whole spread of the fifteen methods it is being used to order.

Diagnostic generalization was assessed in four external cohorts on three other platforms; one of them has a tumor-to-normal ratio close to nine to one, which limits the interpretation of its area under the curve. Prognostic assessment used four external cohorts, but adjustment for clinical covariates was possible in none of them because none deposited stage, grade, or age data alongside survival. The adjusted analysis therefore rests on the discovery cohort, where the risk group remained associated with survival after adjustment, but the survival data also contributed to selection. Establishing that the panel adds information beyond stage and grade requires an external cohort with covariates deposited and a pre-specified threshold, since the median split used here is not a clinically applicable cut-point.

Dependence on a single array platform is a limitation of the method applied here and not only of the cohort. The screen, the candidate pool and the selection are all computed on Affymetrix Human Gene 1.0 ST data, so what that platform measures, and how its probes are annotated, enters every result downstream of it. The mapping described above is the concrete form of that dependence. A pseudogene can reach a twenty-gene panel because a probe reports a signal the annotation attributes to the wrong locus; no downstream measure would reveal it because each one is computed from the same mapping. Transfer to external cohorts is limited by the same property, since a gene absent from another platform’s annotation cannot contribute regardless of its expression. A panel selected on one platform represents a hypothesis about genes rather than probes, and confirming it requires measurement using a technology that does not share this one’s annotation.

Five implementation choices affect enough of the result to be reported separately. Four act inside the search, applied cumulatively together with the one operator introduced alongside them. The crossover is uniform on the symmetric difference, so a child differs from both parents even when the panel sizes match. The returned solution is drawn from an archive scored under one fixed reference vector and not under the weights of its own generation. The concordance objective is beta-weighted. Duplicate chromosomes are removed before evaluation. The operator added is a periodic one-gene local search. Table S2C gives the movement each contributes. Its raised mutation rate row is a change to the base probability *p_m* from 0.15 to 0.25 and not the addition of the adaptive rule of step 3e, which did not activate in any run reported here. The candidate pool was fixed at 300 genes before the comparisons reported here were run, so the selection-stability figures are conditional on that choice. Table S5A,C controls for pool membership and Table S8C for network structure, but none controls for pool size.

The weights of the fitness function and the parameters of the adaptive mechanism were fixed before the reported runs and were not reselected inside the folds of the nested protocol. Residual optimism from that choice is not included in the optimism reported for the nested protocol and is bounded by the spread observed when the parameters are varied one at a time. Reselecting them within each outer fold would remove it, but at a computational cost proportional to the size of the grid. The interaction matrix is taken from the STRING v11.0 release files at the combined score, with no manually added pairs. The same matrix is used for every method, so the comparison between methods does not depend on it. However, the absolute level of network coherence does. Interaction databases record what has been studied, so any network objective is biased towards genes already associated with disease, and a panel selected under such an objective will tend to recover established biology. This limits what the network term can contribute to the discovery of previously unreported candidates, which is a different task from the one addressed here.

Two of the three ablation variants do not isolate a single component. Removing the concordance term leaves the correlation between objectives undefined, so the adaptive mechanism reverts to its neutral state and is disabled alongside the term under evaluation. Removing the network term withdraws the interaction matrix from the fitness function and from the mutation operator together. What that contrast measures is therefore the network component as a whole, not the fitness term on its own, and the residual coherence of 0.0115 is what an unguided search over the same candidate pool produces. Neither variant is a clean isolation, and the contributions attributed to those two components should be interpreted with that in mind.

Five directions for future investigation follow. Reselecting the fitness weights within each outer fold would fold the remaining parameter-selection optimism into the reported estimate. Repeating the network objective at a higher STRING confidence threshold would test whether it depends on weakly supported associations. Assembling a prognostic cohort with deposited stage and grade would allow the adjusted analysis to be repeated outside the discovery series. Beyond the retrospective series used here, the panel would need evaluation in a larger independent cohort, and then, before any clinical use, in a prospective study with a cut-point fixed in advance. Experimental validation of the individual genes is a separate requirement that transcriptomic data cannot meet. Finally, the observation that discrimination does not separate these methods was made on one cohort in one disease. Whether it holds more widely is what would establish whether reproducibility and network coherence deserve the weight given to them here.

## 5. Conclusions

The main finding of this study is that uniting diagnostic accuracy, prognostic concordance and network coherence within one search, rather than optimizing them separately, changes which panel is returned without changing how well that panel discriminates. On discrimination, the fifteen panels compared here are not distinguishable from one another, among which the BIMS-GA scores neither the best nor the worst. Where these methods differ is in whether the genes they select interact. Coherence differs between methods by two orders of magnitude and in every one of twenty runs exceeds the best comparator: it is the one axis that does separate them. Selection stability does not, at the number of runs used in this study, because the ten-seed figures could not be replicated on a second block.

The ablation does not locate the two mechanisms equally well. The network term governs coherence at a small and measurable cost in fit to the cohort it was selected on, which it returns in cohorts it was not. The Wilcoxon signed-rank test over the ten seeds separates that contrast from chance, and the permutation test over run labels separates the term’s effect on stability. Adaptive weighting differs. Holding the weights fixed leaves discrimination unchanged and lowers the mean between-run agreement, but the difference does not reach significance over ten runs. What those runs showed was a change in the shape of the distribution rather than in its center: two runs failed outright instead of each run degrading. This information is provided as a description, as this study is not powered to test it.

The resulting twenty-gene panel is a single adhesion and matrix-remodeling program rather than a set of independent markers. It transfers to four external diagnostic cohorts across three platforms and separates survival in four external prognostic cohorts, decisively where the model is fitted within each cohort and in two of the four where its coefficients are carried from the discovery data. Its probabilities are not rejected by a calibration test under the classifier used throughout, although its expected calibration error places it fifteenth of the eighteen panels. Whether these properties are worth the discrimination they do not provide is a question about the intended use of a biomarker panel; though the question remains unresolved, this study lays out evidence needed to answer it. Answering this question would require a larger independent cohort than any used here. Establishing network coherence as a general criterion, rather than one demonstrated on a single disease, would require the same comparison on other tumor types and other platforms.

## Figures and Tables

**Table 1:** Representative studies on computational biomarker discovery for PDAC and related multi-objective feature selection methods (2018–2025).

Study	Disease	Method	Objectives	Survival Validation	PPI Network	Dataset
Zhao et al. [19]	PDAC	NMF + Deep Learning	Classification	KM (no C-index)	No	GEO (*n* = 1268)
Wang et al. [20]	PDAC	SVM-RFE + LDM-RFE	Accuracy	KM (partial)	No	GSE15471 + GSE28735
Wu et al. [16]	PDAC	LASSO-Cox	Survival	KM + C-index	No	TCGA-PAAD + GEO
Zhang et al. [21]	PDAC	mRMR + SVM	Accuracy + AUC	No	No	GEO + TCGA
Wang & Liu [7]	GBM/ESCC/CRC ‡	RLasso-Cox	Survival	KM + C-index	KEGG + HuRI	TCGA + GEO
Huang et al. [17]	PDAC	WGCNA + Cox	Survival	KM + C-index	STRING †	TCGA + GSE62452
Cattelani & Fortino [22]	Breast	NSGA2-CHS	Balanced acc. + size	No	No	TCGA-BRCA + SCAN-B
Cattelani et al. [23]	Breast/Kidney/Lung/Ovarian	NSGA2-CHS	Balanced acc. + size	No	No	TCGA + GEO
Yablecovitch et al. [24]	PDAC	ELISA panel	AUC + Sensitivity	KM (no C-index)	No	Clinical (*n* = 235)
Solomon et al. [25]	PDAC	DL + urinary biomarkers	Accuracy + AUC	KM (post hoc)	No	Urinary (tabular)
Angelioudaki et al. [26]	PDAC/pNEN	RF-FS + classifiers	AUC + Balanced acc.	No	No	EV-based (*n* = 164)
Cattelani & Fortino [18]	Breast/Kidney	DOSA-MO (NSGA3-CHS)	Balanced acc. + C-index + size	C-index	No	TCGA + SCAN-B
This study	PDAC	BIMS-GA	AUC + C-index + PPI	KM + C-index (4 ext. cohorts)	STRING v11.0	GEO (×7) + TCGA-PAAD

Note: NMF: non-negative matrix factorization; EV: extracellular vesicle; SCAN-B: Sweden Cancerome Analysis Network—Breast; KM: Kaplan–Meier; DL: deep learning; WGCNA: weighted gene co-expression network analysis; ELISA: enzyme-linked immunosorbent assay; RF-FS: random forest feature selection; pNEN: pancreatic neuroendocrine neoplasm; GBM: glioblastoma; ESCC: esophageal squamous cell carcinoma; CRC: colorectal cancer; KEGG: Kyoto Encyclopedia of Genes and Genomes; HuRI: Human Reference Interactome. “PPI Network” = PPI coherence incorporated as a selection objective during feature search (not merely post hoc annotation). † STRING used for hub-gene visualization only; not a direct criterion for the final 7-gene Cox model [17]. ‡ Non-PDAC study (GBM, ESCC, colorectal) included as methodological precedent for network-aware survival feature selection.

**Table 2:** Characteristics of the transcriptomic cohorts.

Dataset	Platform	Tumor (*n*)	Non-Tumor (*n*)	Total	Survival (*n*)	Events	Purpose
GSE62452	GPL6244	69	61	130	65	49	Discovery (both objectives)
GSE15471	GPL570	36	36	72	—	—	External diagnostic
GSE16515	GPL570	36	16	52	—	—	External diagnostic
GSE62165	GPL13667	118	13	131	—	—	External diagnostic
GSE71729 (diagnostic)	GPL20769	145	46	191	—	—	External diagnostic
TCGA_PAAD	RNA-seq	178	0	178	178	93	External prognostic
GSE79668	GPL11154	51	0	51	51	45	External prognostic
GSE85916	GPL13667	79	0	79	79	57	External prognostic
GSE71729 (prognostic)	GPL20769	123	0	123	123	83	External prognostic

Note: Survival (*n*) is the denominator of the Events column. GSE62452 supplies both objectives, so its 49 events belong to the 65 tumors with deposited survival and not all 69 tumors. GSE71729 contributes two evaluation sets from one series, and they are not independent: the Total column sums to 1007 arrays while only 884 are distinct, the difference being the 123 GSE71729 arrays counted in both roles. TCGA-PAAD is deposited as 183 samples; the 4 adjacent normal samples are unused here, and one tumor was excluded due to a recorded survival time of zero, leaving 178. GSE15471 contains 78 arrays from 36 patients: 1 per patient and tissue.

**Table 3:** Component ablation: ten-seed means and paired differences from the full model.

Variant	AUC	Δ AUC	*p* (AUC)	Same Sign (AUC)	C-Index	Δ C-Index	*p* (C-Index)	Same Sign (C-Index)	PPI Coherence	Δ PPI Coherence	*p* (PPI Coherence)	Same Sign (PPI Coherence)
BIMS-GA (Full)	0.9300	—	—	—	0.7036	—	—	—	0.4558	—	—	—
BIMS-GA-NCI	0.9302	−0.0001	0.8457	4/10	0.7058	−0.0022	0.1113	8/10	0.4655	−0.0097	0.3223	6/10
BIMS-GA-NPPI	0.9417	−0.0117	0.0020	10/10	0.7561	−0.0524	0.0020	10/10	0.0115	+0.4443	0.0020	10/10
BIMS-GA-FW	0.9285	+0.0016	0.2969	6/10	0.7046	−0.0010	0.8125	4/10	0.4048	+0.0511	0.1562	5/10

Note: Ten seeds per variant, paired by seed. Each quantity is the mean over the ten runs, the mean paired difference from the full model, the Wilcoxon signed-rank *p*, and the number of seeds on which the difference runs in the direction of that mean. *p* = 0.002 is the smallest value the test can return on ten pairs. Table S2E carries the same quantities for the two external axes.

**Table 4:** BIMS-GA compared with fourteen alternative feature selection methods on the discovery cohort.

Method	AUC (95% CI)	C-Index (95% CI)	PPI Coherence	Interactions	Clustering	Isolated	DeLong *p* vs. BIMS-GA	Cohen’s *d*
SVM-RFE	0.9656 (0.9313–0.9922)	0.7053 (0.6137–0.7894)	0.0049	1	0.000	18	0.0604	3.240
Binary GWO	0.9644 (0.9330–0.9879)	0.7289 (0.6354–0.8146)	0.0221	5	0.117	14	0.0421	3.012
Binary PSO	0.9591 (0.9225–0.9873)	0.7283 (0.6288–0.8193)	0.0053	2	0.000	16	0.2289	2.939
NSGA-III	0.9551 (0.9154–0.9865)	0.7507 (0.6552–0.8368)	0.1628	37	0.386	9	0.3367	3.014
NSGA-II	0.9506 (0.9134–0.9822)	0.7664 (0.6871–0.8401)	0.1555	36	0.503	5	0.5346	2.741
LASSO	0.9458 (0.9019–0.9800)	0.6857 (0.5863–0.7803)	0.0084	2	0.000	16	0.6927	2.745
BIMS-GA (Full) †	0.9418 (0.8962–0.9760)	0.6964 (0.5977–0.7855)	0.4798	111	0.720	0	—	2.539
MOEA/D	0.9361 (0.8890–0.9723)	0.7003 (0.6040–0.7887)	0.1919	47	0.553	3	0.5677	2.476
MLP (perm. importance)	0.9332 (0.8869–0.9709)	0.6952 (0.6032–0.7845)	0.0146	4	0.000	14	0.5026	2.372
Attention-based FS	0.9301 (0.8811–0.9730)	0.6846 (0.5757–0.7790)	0.0144	3	0.150	17	0.3851	2.522
LassoNet	0.9285 (0.8831–0.9688)	0.6896 (0.5943–0.7811)	0.0132	3	0.000	15	0.2239	2.411
DeepPINK	0.9256 (0.8800–0.9662)	0.7277 (0.6358–0.8043)	0.0052	1	0.000	18	0.1982	2.320
ANOVA F-test	0.9173 (0.8625–0.9604)	0.6857 (0.5942–0.7689)	0.0510	10	0.250	15	0.0827	2.146
Random Forest	0.9168 (0.8623–0.9629)	0.6829 (0.5959–0.7667)	0.0538	11	0.250	13	0.0360	2.216
mRMR	0.9157 (0.8575–0.9628)	0.6504 (0.5486–0.7504)	0.0128	3	0.000	15	0.0546	2.266

Note: All methods select twenty genes from the same 300-gene candidate pool and are scored identically. The AUC comes from pooled out-of-fold logistic regression, the C-index from a ridge Cox model on the discovery survival data, and PPI coherence is the mean STRING v11.0 confidence over all pairs. Intervals are 2000-sample bootstrap percentiles. Two of the fourteen comparisons reach significance: Binary GWO (*p* = 0.042) and Random Forest (*p* = 0.036). † Proposed method.

**Table 5:** Diagnostic classification performance of the panel under five-fold cross-validation.

Model	AUC (95% CI)	Average Precision	Accuracy	Sensitivity	Specificity	F1	MCC
Logistic Regression	0.9418 (0.8962–0.9760)	0.9498	0.8846	0.8841	0.8852	0.8905	0.7687
Random Forest	0.9136 (0.8597–0.9614)	0.9274	0.8692	0.8696	0.8689	0.8759	0.7378
MLP	0.9107 (0.8593–0.9562)	0.9203	0.8538	0.8696	0.8361	0.8633	0.7064
Gradient Boosting	0.9033 (0.8409–0.9557)	0.9029	0.8538	0.8551	0.8525	0.8613	0.7069
SVM (RBF)	0.9009 (0.8411–0.9526)	0.9079	0.8538	0.8696	0.8361	0.8633	0.7064

**Table 6:** External validation of the panel in seven independent series contributing eight evaluation sets.

Task	Cohort	*n*	Events	Genes	AUC/C-Index	95% CI	HR (95% CI)	Log-Rank *p*
Diagnostic	GSE15471	72	—	20/20	0.9113	0.8355–0.9691	—	—
	GSE16515	52	—	20/20	0.9358	0.8604–0.9844	—	—
	GSE62165	131	—	20/20	0.9987	0.9934–1.0000	—	—
	GSE71729 (diagnostic)	191	—	20/20	0.8295	0.7649–0.8882	—	—
Prognostic	TCGA_PAAD	178	93	20/20	0.6983	0.6414–0.7526	3.06 (1.97–4.75)	1.94 × 10^−7^
	GSE79668	51	45	20/20	0.726	0.6375–0.8038	3.85 (2.00–7.41)	1.88 × 10^−5^
	GSE85916	79	57	20/20	0.7147	0.6362–0.7852	2.92 (1.67–5.12)	9.17 × 10^−5^
	GSE71729 (prognostic)	123	83	20/20	0.6971	0.6269–0.7629	4.13 (2.56–6.67)	6.96 × 10^−10^

Note: No cohort in this table contributed to selection. Diagnostic predictions come from a logistic regression fitted on the 130 discovery samples and applied without refitting. Prognostic figures come from a ridge-penalized Cox model fitted within each cohort.

**Table 7:** The twenty-gene panel, ordered by selection frequency across ten independent runs.

Gene	Selection Frequency	Runs	Known Function	PDAC Relevance
*LAMC2*	1.00	10/10	Laminin-332 gamma2 chain	Poor overall survival; gemcitabine resistance [51]
*ITGB4*	1.00	10/10	Integrin beta4	Hemidesmosomal anchor
*COL17A1*	1.00	10/10	Collagen XVII	Hemidesmosomal anchor
*LAMA3*	1.00	10/10	Laminin-332 alpha3 chain	Basement-membrane adhesion; poor prognosis [52]
*FN1*	1.00	10/10	Fibronectin	Matrix deposition; hub gene [53]
*ITGA2*	1.00	10/10	Integrin alpha2	Proposed therapeutic target; hub gene [53]
*ITGA11*	1.00	10/10	Integrin alpha11	—
*ITGB5*	1.00	10/10	Integrin beta5	—
*PLAU*	1.00	10/10	Urokinase plasminogen activator	Pericellular proteolysis
*ITGA3*	1.00	10/10	Integrin alpha3	alpha3beta1 receptor for laminin-332 [54]
*PLEC*	1.00	10/10	Plectin; cytoskeletal linker	Hemidesmosomal anchor; biomarker by phage display [55]
*MMP14*	1.00	10/10	Membrane-type 1 matrix metalloproteinase	Pericellular proteolysis
*LAMB3*	1.00	10/10	Laminin-332 beta3 chain	Basement-membrane adhesion; proposed target [53]
*ITGB1*	1.00	10/10	Integrin beta1	alpha3beta1 receptor for laminin-332 [54]
*SDC1*	0.90	9/10	Syndecan-1	—
*MET*	0.90	9/10	Hepatocyte growth factor receptor	—
*CTTN*	0.90	9/10	Cortactin; actin dynamics	—
*ACTN4*	0.80	8/10	Alpha-actinin-4	—
*VCAN*	0.60	6/10	Versican	Matrix deposition; hub gene [53]
*THBS2*	0.60	6/10	Thrombospondin-2	Early detection with CA19-9 [56,57]

Note: Selection frequency is the proportion of ten runs in which the gene entered the panel. Known Function is the molecular identity of the gene product. PDAC Relevance carries a reference where a PDAC-specific result for that gene is cited; a dash means it cites no entry. Neither column is computed. Enriched terms are in Table S11, tested against the candidate pool as background.

## Data Availability

The gene expression datasets analyzed in this study are publicly available from the NCBI Gene Expression Omnibus (GEO) under accession numbers GSE62452, GSE15471, GSE16515, GSE62165, GSE71729, GSE79668, and GSE85916 (GSE62452 accessed on 29 May 2026; the remaining datasets accessed on 24 August 2026), and from The Cancer Genome Atlas under project TCGA-PAAD (accessed on 29 May 2026). Protein–protein interaction data were retrieved from the STRING v11.0 release files, and functional annotation from the Enrichr gene set libraries, both accessed on 24 August 2026.

## Supplementary Materials

The following supporting information can be downloaded at https://www.mdpi.com/article/10.3390/life16101716/s1, Figure S1: Sensitivity of the adaptive weighting to the correlation window *W* and the threshold *rho_thresh*; Figure S2: BIMS-GA convergence and adaptive weight dynamics across ten independent runs; Figure S3: Out-of-fold discrimination of all eighteen twenty-gene panels under one classifier; Figure S4: Calibration and net benefit of the BIMS-GA twenty-gene panel under five classifiers; Table S1: Cohort screening and pre-processing checks; Table S2: Ablation contrasts, multivariable survival and the effect of each implementation choice; Table S3: Sensitivity to the network weight *w*_3_; Table S4: Sensitivity to the adaptive weighting, the genetic operators and the comparator settings; Table S5: Null distributions and the multiplicity-corrected comparisons; Table S6: Computational cost, and every method’s panel scored on calibration, transfer, stability and network topology; Table S7: The nested cross-validation protocol; Table S8: Selection stability under perturbation of the data and of the network; Table S9: Classifier comparison and operating characteristics; Table S10: Selection stability across ten and twenty runs, with the permutation tests; Table S11: Functional enrichment of the panel against the candidate pool.
