# Supplementary material for: BIMS-GA: Adaptive Multi-Objective Optimization for Network-Aware Pancreatic Ductal Adenocarcinoma Biomarker Discovery
Source: Life (Basel). 2026 Oct 14;16(10):1716. doi: 10.3390/life16101716 (PMC13705738; doi:10.3390/life16101716)
Supplement: Supplementary file 1 [file life-16-01716-s001.zip › life-4459707-supplementary.pdf]

Supplementary Materials for

BIMS-GA: Adaptive Multi-objective Optimization for Network-Aware Pancreatic Ductal Adenocarcinoma Biomarker Discovery

Teerapun Saeheaw

Department of Teacher Training in Mechanical Engineering, King Mongkut's University of Technology North Bangkok, Bangkok 10800, Thailand

Email: teerapun.s@fte.kmutnb.ac.th

Supplementary Figures S1-S4 show the sensitivity of the adaptive weighting, the convergence of the search, the out-of-fold discrimination of all eighteen panels and the calibration of the reported panel. Tables S1-S11 report cohort screening, algorithm sensitivity, computational cost, nested validation, calibration, external transfer and selection stability for all eighteen panels, and the network and functional characterization of the reported panel. These materials are available online with the article.

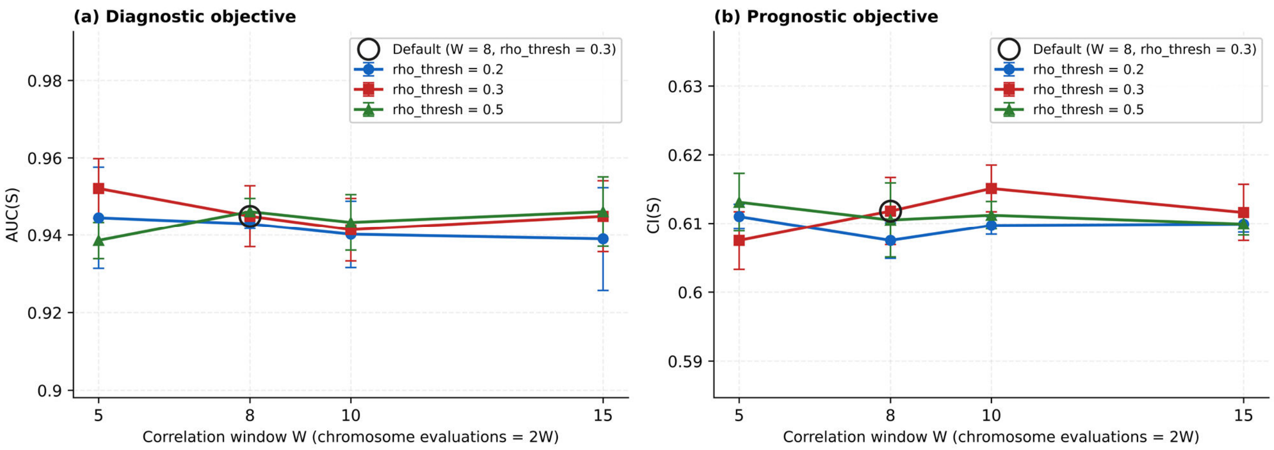

Figure S1. Sensitivity of the adaptive weighting to the correlation window *W* and the threshold *rho_thresh*. (a) Diagnostic objective, AUC(S), and (b) concordance objective, CI(S), at each of the twelve settings, three independent runs per setting, shown as the mean with an error bar of one standard deviation; the setting used throughout is circled.

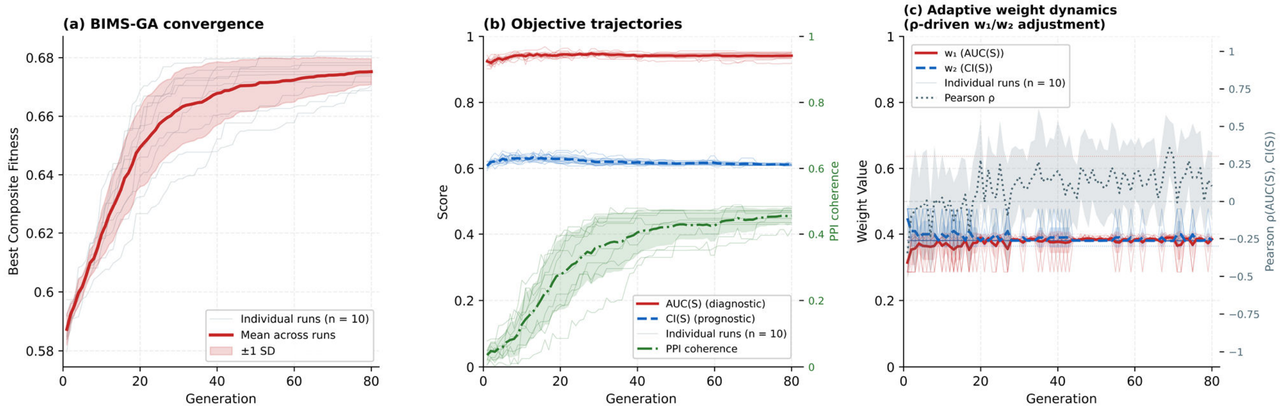

Figure S2. BIMS-GA convergence and adaptive weight dynamics across ten independent runs, random seeds 42 to 105. (a) Best composite fitness per generation for each run, with the mean and a band at one standard deviation. (b) The three objective values of the best chromosome and (c) the inter-objective correlation and the two normalized weights it controls, each the mean over the same ten runs with a band at one standard deviation.

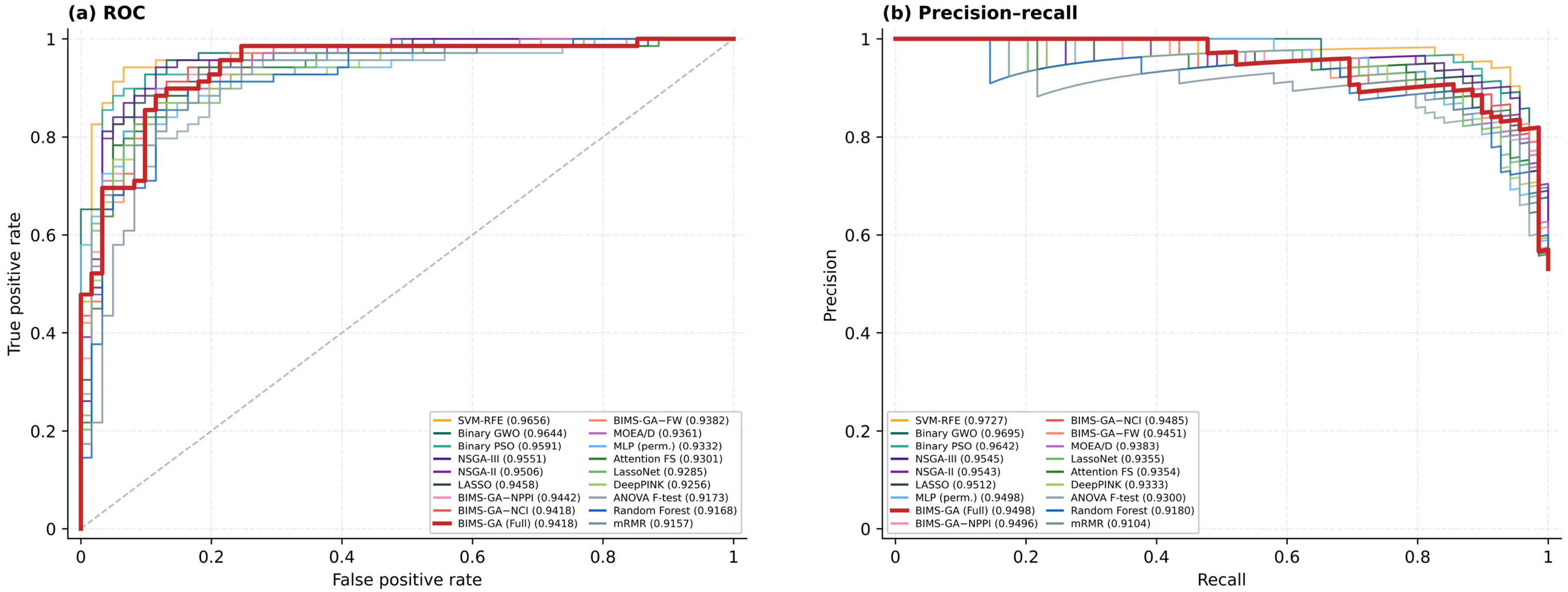

Figure S3. Out-of-fold discrimination of all eighteen twenty-gene panels under one classifier, logistic regression. (a) Receiver operating characteristic curves. (b) Precision–recall curves. The legend gives the average precision in (b) and, in (a), the area under the pooled probability vector each curve requires, which is not the quantity Figure 2 reports; the two differ for the three ablation variants and agree for every other panel.

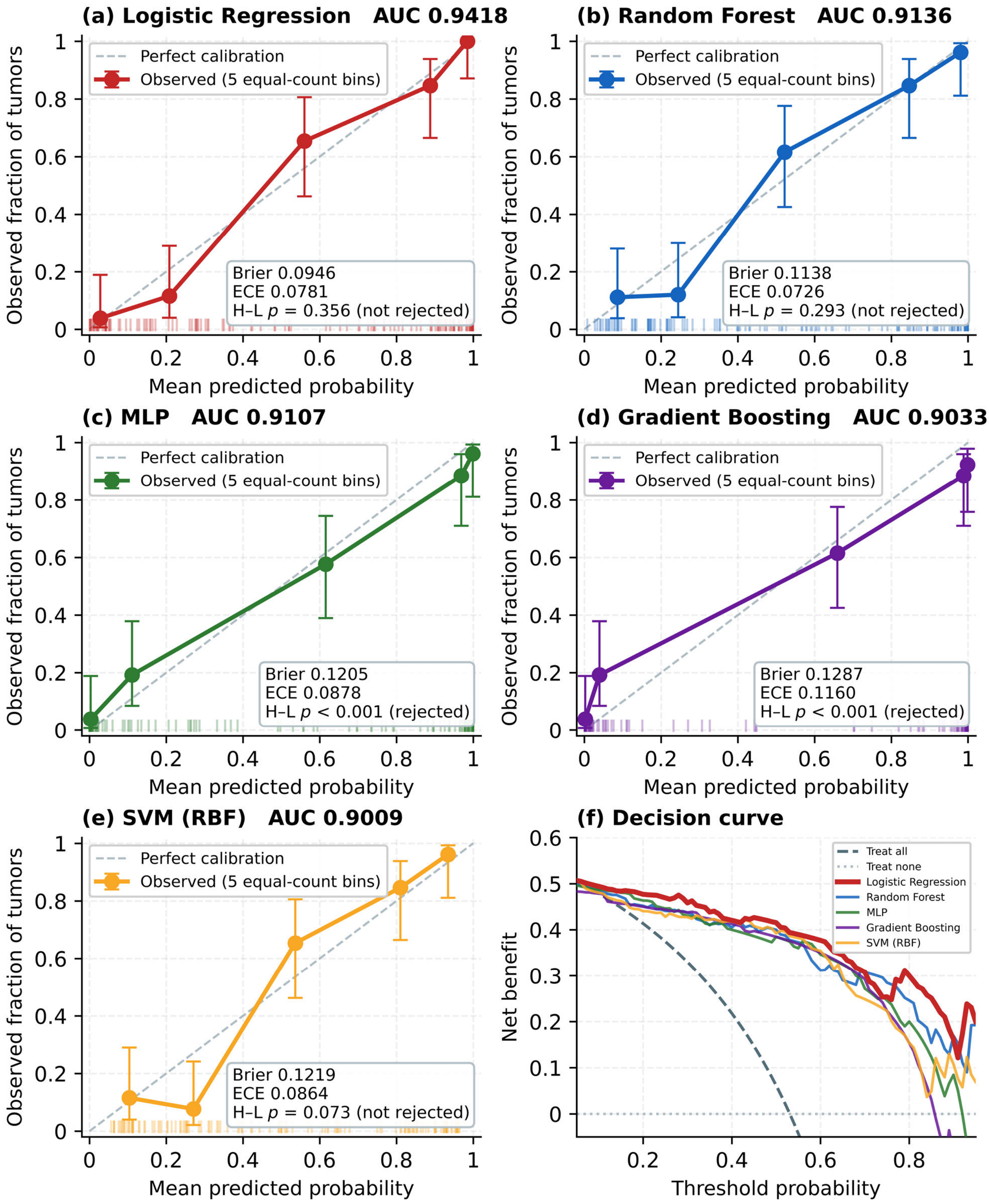

Figure S4. Calibration and net benefit of the BIMS-GA twenty-gene panel under five classifiers. (a-e) Reliability diagram of each classifier, five equal-count bins with Wilson intervals, annotated with the Brier score, the expected calibration error and the Hosmer-Lemeshow *p*. (f) Decision curve against the strategies of treating every patient and of treating none. The panel is held fixed and the classifier varied.

Table S1. Cohort screening and preprocessing checks.

Table S1A. Sample-level overlap screen between cohorts

Panel A · GSE28735 against GSE62452

Criterion	Result
Pairs tested	90
Constant GSM accession offset	815,201 (90/90 pairs)
One-to-one assignment	90/90
Tissue label agreement	90/90
Probes used	28,869 (all probes common to both series)
Matched-pair correlation, range	0.9845-0.9907
Matched-pair correlation, mean	0.9895
Minimum margin over the runner-up	0.0070
Maximum within-series correlation	0.9880
Matched pairs above the within-series maximum	84/90
Sensitivity: top-5,000 variance probes, matched-pair	0.9738-0.9831
Sensitivity: top-5,000 variance probes, within-series	0.9771
Sensitivity: margin over the runner-up	0.0222
Sensitivity: pairs above the within-series maximum	88/90

Panel B · Every pair of cohorts used

Cohort A	Cohort B	Genes in common	Max. cross correlation	Max. within A	Max. within B	Pairs above within-cohort ceiling	Verdict
GSE62452	GSE15471	14521	0.4474	0.8045	0.9767	0	no sample-level overlap
	GSE16515	14521	0.5526	0.8045	0.9862	0	no sample-level overlap
	GSE62165	13750	0.4993	0.8075	0.9839	0	no sample-level overlap
	GSE71729 (diagnostic)	13115	0.3823	0.8077	0.9858	0	no sample-level overlap
	TCGA-PAAD	14013	0.4204	0.8019	0.8451	0	no sample-level overlap
	GSE79668	13811	0.5385	0.8011	0.9630	0	no sample-level overlap
	GSE85916	13750	0.3515	0.8075	0.9587	0	no sample-level overlap
GSE62452	GSE71729 (prognostic)	13115	0.3823	0.8077	0.9851	0	no sample-level overlap
GSE15471	GSE16515	22875	0.8469	0.9818	0.9863	0	no sample-level overlap
	GSE62165	16290	0.7267	0.9785	0.9828	0	no sample-level overlap
	GSE71729 (diagnostic)	15973	0.6827	0.9785	0.9861	0	no sample-level overlap
	TCGA-PAAD	16695	0.2639	0.9787	0.8266	0	no sample-level overlap
	GSE79668	16877	0.6733	0.9803	0.9680	0	no sample-level overlap
	GSE85916	16290	0.5976	0.9785	0.9609	0	no sample-level overlap
	GSE71729 (prognostic)	15973	0.6773	0.9785	0.9861	0	no sample-level overlap
GSE16515	GSE62165	16290	0.8029	0.9870	0.9828	0	no sample-level overlap

Cohort A	Cohort B	Genes in common	Max. cross correlation	Max. within A	Max. within B	Pairs above within-cohort ceiling	Verdict
	GSE71729 (diagnostic)	15973	0.7496	0.9870	0.9861	0	no sample-level overlap
	TCGA-PAAD	16695	0.2321	0.9871	0.8266	0	no sample-level overlap
	GSE79668	16877	0.7710	0.9871	0.9680	0	no sample-level overlap
	GSE85916	16290	0.6418	0.9870	0.9609	0	no sample-level overlap
	GSE71729 (prognostic)	15973	0.7496	0.9870	0.9861	0	no sample-level overlap
GSE62165	GSE71729 (diagnostic)	16995	0.7077	0.9826	0.9863	0	no sample-level overlap
	TCGA-PAAD	18583	0.2408	0.9805	0.8161	0	no sample-level overlap
	GSE79668	17269	0.7020	0.9814	0.9690	0	no sample-level overlap
GSE62165	GSE85916	19041	0.6155	0.9794	0.9625	0	no sample-level overlap
	GSE71729 (prognostic)	16995	0.7077	0.9826	0.9863	0	no sample-level overlap
GSE71729 (diagnostic)	TCGA-PAAD	17570	0.2494	0.9861	0.8205	0	no sample-level overlap
	GSE79668	16241	0.6557	0.9867	0.9666	0	no sample-level overlap
	GSE85916	16995	0.5930	0.9863	0.9610	0	no sample-level overlap
	GSE71729 (prognostic)	19749	1.0000	0.9861	0.9861	123	known shared series, prognostic subset of the diagnostic set
TCGA-PAAD	GSE79668	18200	0.2851	0.8104	0.9706	0	no sample-level overlap
	GSE85916	18583	0.2831	0.8161	0.9628	0	no sample-level overlap
	GSE71729 (prognostic)	17570	0.2494	0.8205	0.9861	0	no sample-level overlap
GSE79668	GSE85916	17269	0.6939	0.9690	0.9622	0	no sample-level overlap
	GSE71729 (prognostic)	16241	0.6456	0.9666	0.9867	0	no sample-level overlap
GSE85916	GSE71729 (prognostic)	16995	0.5925	0.9610	0.9863	0	no sample-level overlap

Note. All 90 GSE28735 arrays are re-depositions of GSE62452 arrays. No other pair exceeded the threshold.

Table S1B. Series examined but not analyzed

Series	Platform	*n*	Reason
GSE28735	GPL6244	90	All 90 arrays are re-depositions of GSE62452 arrays (Table S1A)
GSE57495	GPL15048	63	Custom Affymetrix array; GEO provides no annotation table and the deposited matrix is indexed by probe identifiers, so the panel cannot be mapped. 0 of 20 genes match directly
GSE32676	GPL570	32	No non-tumor samples above the ten-per-class minimum

Note. The exclusion of GSE57495 reflects the platform annotation rather than the panel: all twenty genes are protein-coding and map at 20/20 on GPL570, GPL13667, GPL20769 (after symbol resolution), and both RNA-seq cohorts.

Table S1C. Verification of the ComBat adjustment carried in the discovery matrix

Check	After ComBat	Before ComBat	Note
Per-probe correlation	1.0000	—	after vs. before
DE probes (*n*)	1,106	945	Jaccard 0.8544
t-statistic correlation	1.0000	—	
Group-mean difference correlation	1.0000	—	scale ratio 1.0238
Candidate-pool overlap (genes)	300	300	100.0%
Panel out-of-fold AUC	0.9418	0.9418	difference 0.0000

Table S2. Ablation contrasts, multivariable survival and the effect of each implementation choice.

Table S2A. The two readings of the correlation *rho* in the adaptive weighting rule

Definition of rho	AUC	AUC SD	C-index	C-index SD	PPI	PPI SD	Jaccard	Jaccard SD	Kuncheva	Off-neutral generations, mean	Off-neutral generations, range	Generations, total	Alignment	Conflict	Neutral	Alignment, range	Conflict, range	Neutral, range
2*W* most recent chromosome evaluations (as implemented; Eq. 2)	0.9416	0.0106	0.6112	0.0031	0.4558	0.0234	0.8042	0.0653	0.8821	26.6000	23-34	800	198	68	534	13-27	4-10	46-57
W most recent generations (alternative reading)	0.9398	0.0120	0.6176	0.0136	0.4260	0.0939	0.5403	0.3690	0.5821	45.8000	37-60	800	180	278	342	3-36	5-47	20-43

Note. One reading accumulates over the evaluations within a generation, which is the reading the manuscript implements; the other accumulates over generations. Both are reported.

Table S2B. Multivariable Cox regression in the discovery cohort adjusting for stage and grade

Cohort	Model	Covariate	HR (95% CI)	*p*	*n*	Events
GSE62452	Model 0: risk group, full sample (outside the ladder)	risk_group	3.471 (1.748–6.894)	0.0004	65	49
	Model 1: risk group	risk_group	3.326 (1.676–6.600)	0.0006	64	49
	Model 2: + stage	risk_group	3.294 (1.652–6.569)	0.0007	64	49
	Model 2: + stage	stage	1.048 (0.688–1.595)	0.8286	64	49
	Model 3: + stage + grade	risk_group	2.837 (1.345–5.983)	0.0062	64	49
	Model 3: + stage + grade	stage	1.017 (0.663–1.559)	0.9391	64	49
	Model 3: + stage + grade	grade	1.309 (0.781–2.193)	0.3074	64	49

Table S2C. Effect of each implementation choice on the reported figures

Correction applied	AUC	C-index	PPI coherence	Unique evaluations
V1 as submitted	0.9327 ± 0.0128	0.6096 ± 0.0241	0.3179 ± 0.1167	13.7%
+ Uniform crossover	0.9382 ± 0.0125	0.6605 ± 0.0511	0.2099 ± 0.1496	27.2%
+ Reference archive	0.9392 ± 0.0108	0.6588 ± 0.0514	0.2170 ± 0.1492	27.2%
+ Cox-weighted concordance	0.9372 ± 0.0129	0.6262 ± 0.0187	0.3302 ± 0.1151	25.7%
+ Duplicate control	0.9391 ± 0.0061	0.6187 ± 0.0136	0.4032 ± 0.0762	55.8%
+ Raised mutation rate	0.9390 ± 0.0102	0.6146 ± 0.0092	0.4422 ± 0.0665	60.9%
+ Local search (v3)	0.9416 ± 0.0106	0.6112 ± 0.0031	0.4558 ± 0.0234	63.4%

Note. Applied cumulatively. The reference archive row retains 27.2% unique evaluations because it changes the returned panel, not the search space. Non-search corrections (annotation, labels, and aliases) cannot be applied cumulatively.

Table S2D. External prognostic performance under three protocols

Cohort	*n*	Events	Genes	*C* (transfer)	HR (transfer)	*p* (transfer)	*C* (CV)	HR (CV)	*p* (CV)	*C* (apparent)	HR (apparent)	*p* (apparent)
TCGA-PAAD	178	93	20/20	0.5172	1.0200	0.9220	0.6445	1.8300	0.0044	0.6983	3.0600	1.94×10^{-7}
GSE79668	51	45	20/20	0.6114	2.1700	0.0149	0.5545	1.2000	0.5420	0.7260	3.8500	1.88×10^{-5}
GSE85916	79	57	20/20	0.5943	1.3800	0.2270	0.5600	1.4000	0.2030	0.7147	2.9200	9.17×10^{-5}
GSE71729 (prognostic)	123	83	20/20	0.6127	1.7500	0.0118	0.5562	1.4700	0.0798	0.6971	4.1300	6.96×10^{-10}

Note. Transfer applies coefficients fitted once on the discovery data. The cross-validated estimate refits inside each training part of a five-fold split within the cohort. The apparent estimate fits and scores on the same samples and is retained so the effect of the change can be seen.

Table S2E. Every paired ablation contrast, including the two external quantities Table 3 does not carry

Contrast	Quantity	Mean, full	Mean, variant	Mean difference	Seeds full higher	Seeds agreeing	Wilcoxon *p*	Significant
BIMS-GA (Full) − BIMS-GA-NCI	Discovery AUC	0.9300	0.9302	-0.0001	6/10	4/10	0.8457	no
	Discovery C-index	0.7036	0.7058	-0.0022	2/10	8/10	0.1113	no
	External diagnostic AUC	0.9157	0.9170	-0.0013	5/10	5/10	0.6250	no
	External prognostic C-index	0.7106	0.7076	0.0030	8/10	8/10	0.1602	no
	PPI coherence	0.4558	0.4655	-0.0097	4/10	6/10	0.3223	no
BIMS-GA (Full) − BIMS-GA-NPPI	Discovery AUC	0.9300	0.9417	-0.0117	0/10	10/10	0.0020	yes
	Discovery C-index	0.7036	0.7561	-0.0524	0/10	10/10	0.0020	yes
	External diagnostic AUC	0.9157	0.9021	0.0136	7/10	7/10	0.1602	no
BIMS-GA (Full) − BIMS-GA-NPPI	External prognostic C-index	0.7106	0.6957	0.0149	10/10	10/10	0.0020	yes
	PPI coherence	0.4558	0.0115	0.4443	10/10	10/10	0.0020	yes
BIMS-GA (Full) − BIMS-GA-FW	Discovery AUC	0.9300	0.9285	0.0016	6/10	6/10	0.2969	no
	Discovery C-index	0.7036	0.7046	-0.0010	3/10	4/10	0.8125	no
	External diagnostic AUC	0.9157	0.9071	0.0086	2/10	2/10	0.9375	no
	External prognostic C-index	0.7106	0.7046	0.0060	6/10	6/10	0.0312	yes
	PPI coherence	0.4558	0.4048	0.0511	5/10	5/10	0.1562	No

Note. Fifteen contrasts: three variants against the full model on five quantities, each with the mean of both arms, the paired difference, the number of seeds on which the full model is higher, and the Wilcoxon p. The external prognostic contrast is the one that reverses sign against the discovery contrast for BIMS-GA-NPPI, which is the trade-off Section 4.1 rests on.

Table S3. Sensitivity to the network weight w3: the coherence, stability and discrimination exchange

Panel A · Ten runs at each value of w3

*w*3	Runs	AUC	C-index	PPI coherence	Jaccard	Kuncheva
0.0500	10	0.9621 ± 0.0097	0.6743 ± 0.0152	0.0495 ± 0.0457	0.1830	0.2550
0.1000	10	0.9562 ± 0.0082	0.6592 ± 0.0212	0.1777 ± 0.1077	0.2580	0.3430
0.1500	10	0.9484 ± 0.0073	0.6338 ± 0.0203	0.3316 ± 0.1383	0.3330	0.3890
0.2000	10	0.9416 ± 0.0106	0.6112 ± 0.0031	0.4558 ± 0.0234	0.8040	0.8820

Panel B · The three-seed sweep the criterion was applied to

*w*3	AUC	AUC SD	AUC (rep. CV)	C-index	C-index SD	PPI	PPI SD	Criterion score	Jaccard	Selected by criterion
0.0500	0.9699	0.0050	0.9458	0.6641	0.0225	0.0621	0.0762	0.5793	0.1550	Yes
0.1000	0.9588	0.0108	0.9373	0.6739	0.0078	0.1128	0.0870	0.5606	0.2260	
0.1500	0.9421	0.0018	0.9237	0.6209	0.0111	0.4382	0.0427	0.4095	0.6730	
0.2000	0.9449	0.0079	0.9267	0.6118	0.0049	0.4612	0.0285	0.4099	0.7920	

Note. Panel A uses 10 runs per value; Panel B uses 3. AUC (rep. CV) reflects 20 repeats of 5-fold cross-validation with logistic regression. The min–max normalized objective criterion selects w_3 = 0.05. Below w_3 = 0.20, selection stability and network coherence drop sharply. w_3 = 0.20 is used throughout.

Table S4. Sensitivity to the adaptive weighting, the genetic operators and the comparator settings.
Table S4A. Sensitivity to the correlation window *W* and the threshold *rho_thresh*, and to the six scalars of Eq. (3)

Panel A · The correlation window W and the threshold rho_thresh

W	*rho_ thresh*	AUC	AUC SD	C-index	C-index SD	PPI	PPI SD	Jaccard	Kuncheva	Genes in all runs	Runs	Default
5	0.2000	0.9445	0.0131	0.6110	0.0018	0.4609	0.0346	0.7413	0.8393	16	3	
5	0.3000	0.9521	0.0077	0.6075	0.0042	0.4533	0.0287	0.7413	0.8393	16	3	
5	0.5000	0.9386	0.0047	0.6131	0.0042	0.4687	0.0159	0.7918	0.8750	17	3	
8	0.2000	0.9429	0.0012	0.6075	0.0026	0.4716	0.0086	0.7918	0.8750	17	3	
8	0.3000	0.9449	0.0079	0.6118	0.0049	0.4612	0.0285	0.7918	0.8750	17	3	Yes
8	0.5000	0.9461	0.0034	0.6105	0.0054	0.4607	0.0159	0.7655	0.8571	16	3	
10	0.2000	0.9402	0.0086	0.6097	0.0013	0.4729	0.0096	0.8470	0.9107	18	3	
10	0.3000	0.9414	0.0081	0.6151	0.0034	0.4577	0.0044	0.7655	0.8571	16	3	
10	0.5000	0.9433	0.0072	0.6112	0.0020	0.4661	0.0201	0.8182	0.8929	17	3	
15	0.2000	0.9390	0.0133	0.6099	0.0012	0.4739	0.0110	0.8470	0.9107	18	3	
15	0.3000	0.9449	0.0092	0.6116	0.0041	0.4600	0.0032	0.8470	0.9107	18	3	
15	0.5000	0.9461	0.0090	0.6099	0.0016	0.4630	0.0197	0.8470	0.9107	18	3	

Panel B · The six Eq. (3) scalars over thirty configurations

Group	Note	*a1*	*a2*	*c1*	*c2*	*n1*	*n2*	AUC	AUC SD	C-index	C-index SD	PPI	PPI SD	Off-neutral generations	Unique evaluations	Runs
Alignment		0.0400	0.0200	0.3000	0.5000	0.4000	0.4000	0.9402	0.0086	0.6114	0.0042	0.4703	0.0135	29.3	2,018.0	3
		0.0400	0.0400	0.3000	0.5000	0.4000	0.4000	0.9402	0.0086	0.6114	0.0042	0.4703	0.0135	27.0	2,012.0	3
		0.0400	0.0800	0.3000	0.5000	0.4000	0.4000	0.9402	0.0086	0.6114	0.0042	0.4703	0.0135	32.3	2,024.7	3
		0.0800	0.0200	0.3000	0.5000	0.4000	0.4000	0.9402	0.0086	0.6114	0.0042	0.4703	0.0135	30.3	1,997.3	3
		0.0800	0.0800	0.3000	0.5000	0.4000	0.4000	0.9370	0.0066	0.6118	0.0024	0.4739	0.0083	29.0	2,002.7	3
		0.1600	0.0200	0.3000	0.5000	0.4000	0.4000	0.9465	0.0121	0.6123	0.0034	0.4553	0.0316	28.7	2,043.3	3
		0.1600	0.0400	0.3000	0.5000	0.4000	0.4000	0.9362	0.0077	0.6112	0.0020	0.4776	0.0060	25.3	2,040.7	3
		0.1600	0.0800	0.3000	0.5000	0.4000	0.4000	0.9402	0.0086	0.6114	0.0042	0.4703	0.0135	28.0	2,014.0	3
	Default	0.0800	0.0400	0.3000	0.5000	0.4000	0.4000	0.9449	0.0068	0.6118	0.0042	0.4612	0.0247	27.7	2,018.0	9
Conflict		0.0800	0.0400	0.2000	0.4000	0.4000	0.4000	0.9509	0.0088	0.6134	0.0017	0.4386	0.0345	26.3	2,046.0	3
		0.0800	0.0400	0.2000	0.5000	0.4000	0.4000	0.9398	0.0079	0.6129	0.0037	0.4666	0.0145	28.3	2,017.3	3
		0.0800	0.0400	0.2000	0.6000	0.4000	0.4000	0.9489	0.0093	0.6129	0.0006	0.4506	0.0263	30.0	2,067.7	3
		0.0800	0.0400	0.3000	0.4000	0.4000	0.4000	0.9453	0.0085	0.6092	0.0003	0.4630	0.0197	25.3	2,036.7	3
		0.0800	0.0400	0.3000	0.6000	0.4000	0.4000	0.9477	0.0043	0.6153	0.0134	0.4455	0.0425	27.0	2,000.7	3
		0.0800	0.0400	0.4000	0.5000	0.4000	0.4000	0.9453	0.0082	0.6149	0.0080	0.4329	0.0566	25.7	2,049.0	3
		0.0800	0.0400	0.4000	0.6000	0.4000	0.4000	0.9410	0.0055	0.6118	0.0064	0.4431	0.0407	30.0	2,054.0	3
	Ordering violated (*c1* = *c2*)	0.0800	0.0400	0.4000	0.4000	0.4000	0.4000	0.9481	0.0016	0.6093	0.0023	0.4612	0.0152	29.0	2,029.3	6
Neutral		0.0800	0.0400	0.3000	0.5000	0.3000	0.3000	0.9449	0.0048	0.6120	0.0009	0.4609	0.0148	31.0	2,070.7	3
		0.0800	0.0400	0.3000	0.5000	0.3000	0.4000	0.9342	0.0049	0.6228	0.0216	0.4064	0.1288	28.3	2,072.0	3
		0.0800	0.0400	0.3000	0.5000	0.3000	0.5000	0.9429	0.0071	0.6218	0.0223	0.4012	0.1320	26.0	2,071.0	3
		0.0800	0.0400	0.3000	0.5000	0.4000	0.3000	0.9505	0.0081	0.6162	0.0138	0.3980	0.1032	32.7	2,091.0	3
		0.0800	0.0400	0.3000	0.5000	0.4000	0.5000	0.9390	0.0067	0.6103	0.0076	0.4639	0.0297	25.7	2,087.0	3

Group	Note	*a1*	*a2*	*c1*	*c2*	*n1*	*n2*	AUC	AUC SD	C-index	C-index SD	PPI	PPI SD	Off-neutral generations	Unique evaluations	Runs
		0.0800	0.0400	0.3000	0.5000	0.5000	0.3000	0.9461	0.0035	0.6097	0.0046	0.4636	0.0171	23.3	2,030.7	3
Neutral		0.0800	0.0400	0.3000	0.5000	0.5000	0.4000	0.9481	0.0034	0.6084	0.0037	0.4636	0.0172	32.7	2,018.3	3
		0.0800	0.0400	0.3000	0.5000	0.5000	0.5000	0.9497	0.0036	0.6217	0.0144	0.3937	0.0911	32.3	2,085.3	3
Special	Reversed conflict pair	0.0800	0.0400	0.5000	0.3000	0.4000	0.4000	0.9457	0.0086	0.6146	0.0097	0.4247	0.0912	23.3	2,086.3	3
	No alignment tilt	0.0000	0.0000	0.3000	0.5000	0.4000	0.4000	0.9398	0.0048	0.6131	0.0031	0.4648	0.0076	28.0	2,039.0	3

Panel C · The between/within summary, both poolings

Objective	Configurations	SD between configurations	Within-configuration SD (RMS)	Within-configuration SD (mean)	Between/within ratio (RMS)	Between/within ratio (mean)	Between smaller than within
AUC	27	0.0046	0.0073	0.0069	0.6350	0.6700	Yes
C-index	27	0.0037	0.0086	0.0063	0.4330	0.5870	Yes
PPI coherence	27	0.0250	0.0527	0.0378	0.4750	0.6620	Yes

Panel D · Per-branch spread of coherence, with each branch's firing rate

Branch	Configurations	SD of PPI between configurations	Mean PPI	Generation-events in force	Generation-events (total)	Percent in force
Alignment	9	0.0067	0.4688	198	800	24.8
Conflict	8	0.0123	0.4502	68	800	8.5
Neutral	8	0.0340	0.4314	534	800	66.8
Special	2	0.0284	0.4447	—	—	—

Panel E · The configurations that cross a stated constraint

Group	Note	*a1*	*a2*	*c1*	*c2*	*n1*	*n2*	AUC	C-index	PPI	Runs
Alignment	Default	0.0800	0.0400	0.3000	0.5000	0.4000	0.4000	0.9449	0.6118	0.4612	9
Conflict	Ordering violated (*c1* = *c2*)	0.0800	0.0400	0.4000	0.4000	0.4000	0.4000	0.9481	0.6093	0.4612	6
Special	Reversed conflict pair	0.0800	0.0400	0.5000	0.3000	0.4000	0.4000	0.9457	0.6146	0.4247	3
Special	No alignment tilt	0.0000	0.0000	0.3000	0.5000	0.4000	0.4000	0.9398	0.6131	0.4648	3

Note. Thirty configurations at three seeds each, collapsing to twenty-seven distinct settings because the default belongs to all three grids and one conflict cell coincides with a named special cell. The between-configuration spread is smaller than the between-seed spread at a fixed setting on all three objectives: 0.0046 against 0.0073 for AUC, 0.0037 against 0.0086 for C-index and 0.0250 against 0.0527 for network coherence. The second figure of each pair is the root mean square of the per-configuration standard deviations; the mean instead gives 0.0069, 0.0063 and 0.0378, and Panel C carries both.

Table S4B. Sensitivity to the genetic operator settings

Panel A · Each operator setting varied around its default

Parameter	Value	AUC	AUC SD	C-index	C-index SD	PPI	Runs	Default
Crossover rate (p_c)	0.6000	0.9457	0.0045	0.6230	0.0223	0.3962	3	
	0.8000	0.9449	0.0079	0.6118	0.0049	0.4612	3	Yes
	0.9500	0.9536	0.0072	0.6224	0.0194	0.3882	3	
Mutation rate (p_m)	0.0500	0.9532	0.0036	0.6097	0.0018	0.4319	3	
	0.1500	0.9421	0.0067	0.6129	0.0017	0.4598	3	
	0.2500	0.9449	0.0079	0.6118	0.0049	0.4612	3	Yes
	0.3500	0.9469	0.0025	0.6134	0.0042	0.4540	3	
Panel size (k)	10	0.9200	0.0092	0.5899	0.0000	0.8700	3	
	15	0.9307	0.0108	0.6224	0.0218	0.5346	3	
	20	0.9449	0.0079	0.6118	0.0049	0.4612	3	Yes
	25	0.9564	0.0117	0.6258	0.0103	0.2962	3	
Population size (P)	20	0.9358	0.0020	0.6144	0.0114	0.4088	3	
	40	0.9449	0.0079	0.6118	0.0049	0.4612	3	Yes
	60	0.9413	0.0081	0.6108	0.0018	0.4679	3	

Panel B · Range of the mean within each parameter

Parameter	AUC range	C-index range
Crossover rate (p_c)	0.0087	0.0112
Mutation rate (p_m)	0.0111	0.0037
Panel size (k)	0.0364	0.0359
Population size (P)	0.0091	0.0036

Table S4C. Settings of the fourteen comparator methods as implemented, with the rule reducing each to exactly twenty genes

**Method**	**Family**	**Settings as implemented**	**Rule reducing to exactly twenty genes**
ANOVA F-test	Filter	One-way ANOVA F-statistic between tumour and non-tumour groups	Highest twenty by F; ranking by F is monotone in the p-value, so no multiplicity correction can change the selected set
mRMR	Filter	Greedy forward selection maximising mutual information with the class label minus the mean absolute Pearson correlation with the genes already chosen (difference form) [34]	Selection stops at twenty
LASSO	Filter	L1-penalised logistic regression, liblinear solver [35], at most 2,000 iterations	Inverse regularization strength searched by bisection over 0.0001 to 10 for at most sixty steps until exactly twenty coefficients remain non-zero; where no value in the interval yields twenty, the last model fitted is retained and its twenty largest absolute coefficients taken
SVM-RFE	Wrapper	Linear support vector machine; the gene with the smallest squared weight is removed and the model refitted [36]	One gene per step until twenty remain, which is 280 successive refits
Binary PSO	Metaheuristic	Twenty individuals, sixty iterations; inertia weight 0.7, cognitive and social coefficients 1.5, velocities not clamped [37]	The twenty genes with the largest absolute hyperbolic tangent of their coordinates
Binary GWO	Metaheuristic	Twenty individuals, sixty iterations; control parameter decreasing linearly from 2 to 0, each individual moving to the mean of its three displacements towards the current best, second and third solutions [38]	The twenty genes with the largest absolute hyperbolic tangent of their coordinates
NSGA-II	Pareto-based	Same three objectives as BIMS-GA, population forty over eighty generations; survivors by non-dominated sorting, then crowding distance within the front that must be split [10]	Knee point of the returned front: the non- dominated solution closest to the ideal point in normalized objective space
NSGA-III	Pareto-based	As NSGA-II, with the crowding step replaced by association to fifteen reference directions on the	Knee point of the returned front

Method	Family	Settings as implemented	Rule reducing to exactly twenty genes
		three-objective simplex, selecting from the splitting front by smallest perpendicular distance to a reference line [39]	
MOEA/D	Pareto-based	Tchebycheff scalarization over forty weight vectors, formed from the same fifteen simplex directions and completed by resampling; each subproblem updated against a neighborhood of its eight nearest weight vectors [40]	Knee point of the returned front
Random Forest	ML importance	Impurity importance from a forest of 500 trees	The twenty largest importance
MLP (perm. importance)	ML importance	Multilayer perceptron, hidden layers of 64 and 32 units, early stopping, at most 600 iterations; importance averaged over ten permutations of each gene, scored by AUC on the data the network was fitted to	The twenty largest importance
LassoNet	Deep learning	Single hidden layer of 64 units [41]	The sparsest model on the regularization path retaining at least twenty genes, reduced to twenty by the ANOVA F-test where it retains more
DeepPINK	Deep learning	Each gene paired with a knockoff obtained by permuting the sample order of the design matrix; pairwise filter layer into a 64-unit network, 400 full-batch epochs at learning rate 0.01 [42]	Genes ranked by the difference between the squared filter weights of a gene and its knockoff; the twenty largest
Attention-based FS	Deep learning	Two-layer network producing one weight per gene through a softmax across genes; the weighted features feed a 64-unit classifier, 400 full-batch epochs at learning rate 0.005	The twenty genes with the largest mean weight

Note. This table gives the settings; Section 2.3.1 gives what each rule is for and the direction in which each choice would bias a comparator.

Table S5. Null distributions and the multiplicity-corrected comparisons.

Table S5A. Null distribution of the external prognostic result under the apparent protocol, 1,000 random twenty-gene draws from each of two backgrounds

Null	Draws	Mean C-index	C-index 2.5th percentile	C-index 97.5th percentile	Observed C-index	Percentile of panel (C-index)	Mean HR	HR 97.5th percentile	Observed HR	Percentile of panel (HR)	Draws dropped (incomplete coverage)	Proportion significant in all four cohorts
Random 20 genes from the pool	1000	0.7019	0.6763	0.7276	0.7090	69.8	3.3200	4.2560	3.4870	67.5	0	0.9940
Random 20 genes from the transcriptome	851	0.6768	0.6439	0.7099	0.7090	97.2	2.8800	3.7170	3.4870	92.9	149	0.9570

Note. Each draw is carried through the protocol of Section 2.4.2 unchanged. The last column is the proportion of draws significant by log-rank in all four cohorts at once.

Table S5B. Null distribution of the external prognostic result under the cross-validated protocol

Null	Draws	Draws dropped (incomplete coverage)	Mean C-index	C-index 2.5th percentile	C-index 97.5th percentile	Observed C-index	Percentile of panel	Empirical *p*
Random 20 genes from the pool	200	0	0.5681	0.5208	0.6070	0.5788	66.0	0.3400
Random 20 genes from the transcriptome	165	35	0.5367	0.4887	0.5872	0.5788	92.7	0.0727

Table S5C. Null distribution of the external diagnostic transfer

Null	Draws	Draws dropped (incomplete coverage)	Mean AUC	AUC 2.5th percentile	AUC 97.5th percentile	Observed AUC	Percentile of panel	Empirical *p*
Random 20 genes from the pool	1000	0	0.9231	0.8750	0.9491	0.9188	32.9	0.6710
Random 20 genes from the transcriptome	1000	0	0.8360	0.6786	0.9128	0.9188	98.9	0.0110

Note. The classifier is fitted once on the discovery samples and applied without refitting, so this protocol does not reuse the outcome.

Table S5D. Discovery concordance with and without resubstitution

Panel A · Apparent and cross-validated concordance of each variant

Variant	Runs	Apparent	Cross-validated	Optimism
BIMS-GA-FW	10	0.7046 ± 0.0082	0.6027 ± 0.0310	0.1020
BIMS-GA-NCI	10	0.7058 ± 0.0051	0.6114 ± 0.0129	0.0945
BIMS-GA-NPPI	10	0.7561 ± 0.0142	0.6449 ± 0.0336	0.1112
BIMS-GA (Full)	10	0.7036 ± 0.0137	0.6112 ± 0.0270	0.0924

Panel B · Paired contrasts against the full model on both estimates

Contrast BIMS-GA (Full) −	Estimate	Mean paired difference	Seeds with full model higher	Wilcoxon p
BIMS-GA-FW	apparent	-0.0010	3/10	0.8125
BIMS-GA-FW	cross-validated	0.0085	4/10	0.3125
BIMS-GA-NCI	apparent	-0.0022	2/10	0.1113
BIMS-GA-NCI	cross-validated	-0.0002	3/10	0.2656
BIMS-GA-NPPI	apparent	-0.0524	0/10	0.0020
BIMS-GA-NPPI	cross-validated	-0.0337	2/10	0.0137

Note. Apparent estimates fit and score the ridge Cox model on all 65 patients (49 events). Cross-validated estimates refit the model inside each training part of a five-fold split. Both estimates repeat the paired ablation contrast.

Table S5E. The fourteen DeLong comparisons under three thresholds

Method	DeLong *p*	Significant (uncorrected)	Bonferroni threshold	Significant (Bonferroni)	BH-adjusted *p*	Significant (BH)
SVM-RFE	0.0604	No	0.00357	No	0.2114	No
Binary GWO	0.0421	Yes	0.00357	No	0.2114	No
Binary PSO	0.2289	No	0.00357	No	0.4005	No
NSGA-III	0.3367	No	0.00357	No	0.5237	No
NSGA-II	0.5346	No	0.00357	No	0.6114	No
LASSO	0.6927	No	0.00357	No	0.6927	No
MOEA/D	0.5677	No	0.00357	No	0.6114	No
MLP (perm. importance)	0.5026	No	0.00357	No	0.6114	No
Attention-based FS	0.3851	No	0.00357	No	0.5391	No
LassoNet	0.2239	No	0.00357	No	0.4005	No
DeepPINK	0.1982	No	0.00357	No	0.4005	No
ANOVA F-test	0.0827	No	0.00357	No	0.2316	No
Random Forest	0.0360	Yes	0.00357	No	0.2114	No
mRMR	0.0546	No	0.00357	No	0.2114	No

Note. No comparison meets the Bonferroni threshold of 0.00357, and the smallest Benjamini-Hochberg adjusted p is 0.2114.

Table S5F. Null distribution of the network coherence of the panel, 1,000 random twenty-gene draws from the candidate pool

Null	Draws	Mean coherence	SD of coherence	Coherence 2.5th percentile	Coherence 97.5th percentile	Max. coherence	Observed coherence	Percentile of panel	Empirical *p*
Random 20 genes from the pool	1000	0.0129	0.0097	0.0000	0.0374	0.0612	0.4798	100.0	< 0.001

Note. Same construction as the diagnostic null of Table S5C: a thousand twenty-gene draws from the pool, scored and compared against the reported panel. Random draws match the panel on discrimination and on prognosis but not on network structure. The panel's 0.4798 is 37.2 times the mean of the draws and 7.8 times the largest of them. No draw reached the panel, and with a thousand draws the smallest non-zero value the statistic can take is 0.001, so the result is reported as p < 0.001 rather than as p = 0.

Table S6. Computational cost, and every method's panel scored on calibration, transfer, stability and network topology.

Table S6A. Asymptotic complexity of each algorithm step

Step	Complexity	Note
Fitness evaluation of one chromosome	*O(k n log n + k^2)*	five-fold logistic regression on *k* genes, plus the *k* x *k* PPI submatrix
One generation	*O(P k n log n + P k^2)*	*P* chromosomes; memoisation makes the constant smaller than P
Full search	*O(G P k n log n + G P k^2)*	*G* generations
Non-dominated sorting (NSGA-II/III, MOEA/D)	*O(M P^2)* per generation	*M* objectives; not incurred by BIMS-GA, which scalarises

Table S6B. Measured runtime and peak memory of every method

Method	Type	Runtime (s)	Runtime SD (s)	Per generation (s)	Peak Python allocation (MB)	Process RSS (MB)	Unique evaluations	Runs
BIMS-GA (Full)	Proposed / ablation	201.1000	1.2000	2.5140	1.9900	217.9000	2,018	3
BIMS-GA-NCI	Proposed / ablation	162.8000	5.1000	2.0350	1.6400	217.9000	2,011	3
BIMS-GA-NPPI	Proposed / ablation	192.0000	3.1000	2.4000	1.8000	217.6000	2,016	3
BIMS-GA-FW	Proposed / ablation	200.5000	1.5000	2.5060	1.8000	218.8000	2,051	3
ANOVA F-test	Filter	0.0020	—	—	0.8700	219.4000	—	1
LASSO	Filter	0.0320	—	—	0.4400	219.5000	—	1
mRMR	Filter	12.6860	—	—	1.3400	221.1000	—	1
SVM-RFE	Wrapper	2.0090	—	—	1.3600	222.1000	—	1
Random Forest	ML importance	3.6530	—	—	3.6900	220.6000	—	1
MLP (perm. importance)	ML importance	17.4110	—	—	2.5000	225.0000	—	1
Binary PSO	Metaheuristic	96.2630	—	—	1.0400	225.2000	—	1
Binary GWO	Metaheuristic	96.2130	—	—	0.7700	225.3000	—	1
NSGA-II	Pareto MO	288.0270	—	—	1.0100	225.1000	—	1
NSGA-III	Pareto MO	281.4300	—	—	1.0000	225.1000	—	1
MOEA/D	Pareto MO	273.0500	—	—	0.9800	225.1000	—	1
LassoNet	Deep	90.9660	—	—	112.2600	1,219.5000	—	1
DeepPINK	Deep	0.9690	—	—	1.4900	1,222.8000	—	1
Attention-based FS	Deep	1.6500	—	—	0.4500	1,228.1000	—	1

Note. — not applicable: the method was run once, or it has no generations or fitness evaluations.

Table S6C. Empirical scaling in population size and generation count

Axis	Value	Seconds
Population size	10	41.0300
	20	55.4400
	40	86.2800
	80	139.7500
	120	197.0800
Generations	10	58.2300
	20	87.3500
	40	129.4200
	80	197.7700

Table S6D. Fitted scaling models for those two axes

Axis	Linear slope (s)	Linear R^2	Quadratic coefficient	Quadratic R^2
Generations	1.9474	0.9907	-0.0099	0.9993
Population size	1.4114	0.9996	-0.0003	0.9997

Table S6E. Calibration and clinical safety of every method's panel

Method	Family	AUC	Brier	ECE	Average precision	TP	FP	FN	TN	Sensitivity	Specificity	PPV	NPV	Youden's J
BIMS-GA-NPPI	Ablation	0.9534	0.0797	0.0362	0.9587	62	8	7	53	0.8986	0.8689	0.8857	0.8833	0.7674
NSGA-III	Pareto MO	0.9551	0.0757	0.0366	0.9545	62	7	7	54	0.8986	0.8852	0.8986	0.8852	0.7838
MLP (perm. importance)	ML importance	0.9332	0.1030	0.0472	0.9498	61	11	8	50	0.8841	0.8197	0.8472	0.8621	0.7037
LASSO	Filter	0.9458	0.0857	0.0497	0.9512	63	8	6	53	0.9130	0.8689	0.8873	0.8983	0.7819
Attention-based FS	Deep	0.9301	0.0956	0.0513	0.9354	59	8	10	53	0.8551	0.8689	0.8806	0.8413	0.7239
MOEA/D	Pareto MO	0.9361	0.0972	0.0553	0.9383	62	9	7	52	0.8986	0.8525	0.8732	0.8814	0.7510
Binary PSO	Metaheuristic	0.9591	0.0783	0.0580	0.9642	64	8	5	53	0.9275	0.8689	0.8889	0.9138	0.7964
LassoNet	Deep	0.9285	0.1015	0.0600	0.9355	59	8	10	53	0.8551	0.8689	0.8806	0.8413	0.7239
NSGA-II	Pareto MO	0.9506	0.0859	0.0608	0.9543	60	7	9	54	0.8696	0.8852	0.8955	0.8571	0.7548
Random Forest	ML importance	0.9168	0.1114	0.0631	0.9180	59	8	10	53	0.8551	0.8689	0.8806	0.8413	0.7239
BIMS-GA-NCI	Ablation	0.9432	0.0943	0.0633	0.9507	60	7	9	54	0.8696	0.8852	0.8955	0.8571	0.7548
BIMS-GA-FW	Ablation	0.9435	0.0943	0.0640	0.9512	60	7	9	54	0.8696	0.8852	0.8955	0.8571	0.7548
SVM-RFE	Wrapper	0.9656	0.0691	0.0641	0.9727	65	5	4	56	0.9420	0.9180	0.9286	0.9333	0.8601
Binary GWO	Metaheuristic	0.9644	0.0762	0.0744	0.9695	63	6	6	55	0.9130	0.9016	0.9130	0.9016	0.8147
BIMS-GA (Full)	Proposed	0.9418	0.0946	0.0781	0.9498	61	7	8	54	0.8841	0.8852	0.8971	0.8710	0.7693
mRMR	Filter	0.9157	0.1086	0.0794	0.9104	60	10	9	51	0.8696	0.8361	0.8571	0.8500	0.7056
ANOVA F-test	Filter	0.9173	0.1161	0.0821	0.9300	56	10	13	51	0.8116	0.8361	0.8485	0.7969	0.6477
DeepPINK	Deep	0.9256	0.1067	0.0839	0.9333	60	8	9	53	0.8696	0.8689	0.8824	0.8548	0.7384

Note. Each comparator and each ablation variant is scored on the single panel it returns under seed 42, and the full model on its consensus panel, so every row is one panel scored in the same way. Figure 2 builds the three ablation variants by a different rule and gives them different values: BIMS-GA-NPPI reads 0.9534 here and 0.9441 there. The fourteen comparators are built the same way in both and agree exactly, and the full model is one consensus panel under both rules and reads 0.9418 in both, so the difference is a property of the construction rule and not of the scoring.

Table S6F. Transfer of every method's panel to the eight external cohorts

Method	Family	Panels (n)	GSE15471 AUC	GSE16515 AUC	GSE62165 AUC	GSE71729 (diag.) AUC	TCGA-PAAD C-index	GSE79668 C-index	GSE85916 C-index	GSE71729 (prog.) C-index	Mean ext. AUC	Mean ext. C-index	Overall
ANOVA F-test	Filter	1	0.9552	0.9497	0.9987	0.8816	0.6913	0.7412	0.6962	0.7340	0.9463	0.7157	0.8310
NSGA-III	Pareto MO	1	0.9421	0.9427	0.9694	0.8127	0.7252	0.7372	0.6982	0.7085	0.9167	0.7173	0.8170
LassoNet	Deep	1	0.9514	0.9184	0.9993	0.8598	0.6818	0.7340	0.7059	0.6798	0.9322	0.7004	0.8163
MOEA/D	Pareto MO	1	0.9437	0.8958	0.9941	0.8732	0.7009	0.7300	0.6986	0.6902	0.9267	0.7049	0.8158
Random Forest	ML importance	1	0.9537	0.9323	0.9915	0.8789	0.6807	0.6979	0.6628	0.7268	0.9391	0.6920	0.8155
BIMS-GA-NCI	Ablation	4	0.9118	0.9570	0.9932	0.8234	0.6992	0.7256	0.7131	0.6992	0.9214	0.7093	0.8154
mRMR	Filter	1	0.9236	0.9219	0.9961	0.8760	0.6870	0.7412	0.6849	0.6902	0.9294	0.7008	0.8151
BIMS-GA-FW	Ablation	3	0.9226	0.9416	0.9948	0.8285	0.6991	0.7260	0.7112	0.6939	0.9219	0.7076	0.8148
BIMS-GA (Full)	Proposed	1	0.9113	0.9358	0.9987	0.8295	0.6983	0.7260	0.7147	0.6971	0.9188	0.7090	0.8139
Binary GWO	Metaheuristic	1	0.9506	0.9010	0.9987	0.8187	0.7037	0.7308	0.6845	0.7128	0.9172	0.7079	0.8125
Attention-based FS	Deep	1	0.9128	0.8785	1.0000	0.8594	0.6869	0.7700	0.7087	0.6835	0.9127	0.7123	0.8125
DeepPINK	Deep	1	0.9282	0.8854	0.9987	0.8405	0.6901	0.7524	0.6749	0.6788	0.9132	0.6990	0.8061
SVM-RFE	Wrapper	1	0.9090	0.8733	0.9974	0.8486	0.6910	0.7644	0.7333	0.6297	0.9071	0.7046	0.8058
MLP (perm. importance)	ML importance	1	0.8580	0.8681	0.9928	0.8621	0.6796	0.7941	0.7151	0.6709	0.8952	0.7149	0.8051
LASSO	Filter	1	0.9236	0.8681	0.9993	0.8483	0.6761	0.7292	0.6769	0.7051	0.9098	0.6968	0.8033
Binary PSO	Metaheuristic	1	0.9198	0.8420	0.9993	0.8289	0.7034	0.7356	0.7127	0.6790	0.8975	0.7077	0.8026
BIMS-GA-NPPI	Ablation	36	0.9057	0.8300	0.9967	0.8457	0.6991	0.6901	0.6699	0.6953	0.8945	0.6886	0.7915
NSGA-II	Pareto MO	1	0.9144	0.8021	0.9863	0.7745	0.7045	0.7139	0.6946	0.6954	0.8693	0.7021	0.7857

Table S6G. Cross-platform coverage of every method's panel

Method	Family	Panels (n)	Panel size	GSE15471	GSE16515	GSE62165	GSE71729 (diag.)	TCGA-PAAD	GSE79668	GSE85916	GSE71729 (prog.)	Mean coverage (fraction)	Min. genes covered
BIMS-GA (Full)	Proposed	1	20	20	20	20	20	20	20	20	20	1.0000	20
BIMS-GA-NCI	Ablation	4	20	20	20	20	20	20	20	20	20	1.0000	20
BIMS-GA-FW	Ablation	3	20	20	20	20	20	20	20	20	20	1.0000	20
MLP (perm. importance)	ML importance	1	20	20	20	20	20	20	19	20	20	0.9938	19
Binary PSO	Metaheuristic	1	20	19	19	20	20	20	20	20	20	0.9875	19
ANOVA F-test	Filter	1	20	20	20	20	20	20	18	20	20	0.9875	18
NSGA-II	Pareto MO	1	20	20	20	19	20	20	20	19	20	0.9875	19
Random Forest	ML importance	1	20	20	20	20	19	20	19	20	19	0.9812	19
Binary GWO	Metaheuristic	1	20	20	20	19	19	19	19	19	19	0.9625	19
MOEA/D	Pareto MO	1	20	19	19	19	19	19	19	19	19	0.9500	19
mRMR	Filter	1	20	19	19	19	19	19	18	19	19	0.9437	18
NSGA-III	Pareto MO	1	20	18	18	18	18	19	19	18	18	0.9125	18
Attention-based FS	Deep	1	20	19	19	18	18	18	17	18	18	0.9062	17
SVM-RFE	Wrapper	1	20	18	18	18	18	18	18	18	18	0.9000	18
LassoNet	Deep	1	20	18	18	18	18	19	17	18	18	0.9000	17
BIMS-GA-NPPI	Ablation	36	20	17.8	17.8	17.8	17.8	18.8	17.8	17.8	17.8	0.8951	17.8
DeepPINK	Deep	1	20	17	17	16	17	18	16	16	17	0.8375	16
LASSO	Filter	1	20	16	16	17	16	18	15	17	16	0.8187	15

Note. Each cohort column gives the number of the twenty panel genes measurable in that cohort; mean coverage is the fraction over all eight cohorts.

Table S6H. Selection stability of every method under both protocols, with tests against BIMS-GA

Method	Family	Protocol	Runs	Jaccard	Jaccard SD	Jaccard median	Jaccard min.	Jaccard max.	Kuncheva	Kuncheva SD	Distinct genes	Genes in all runs
BIMS-GA (Full)	Proposed	seeds	10	0.8042	0.0653	0.8182	0.6667	0.9048	0.8821	0.0432	29	14
BIMS-GA-NCI	Ablation	seeds	10	0.7707	0.0664	0.7391	0.6667	0.9048	0.8595	0.0456	28	14
Random Forest	ML importance	seeds	10	0.5657	0.0698	0.6000	0.3793	0.7391	0.7000	0.0623	40	7
BIMS-GA-FW	Ablation	seeds	10	0.4668	0.3207	0.5385	0.0256	0.9048	0.5310	0.3672	48	0
NSGA-III	Pareto MO	seeds	10	0.2707	0.0855	0.2500	0.1111	0.4286	0.3774	0.1137	70	0
NSGA-II	Pareto MO	seeds	10	0.2661	0.0606	0.2903	0.1429	0.3793	0.3750	0.0814	70	1
DeepPINK	Deep	seeds	10	0.2289	0.0663	0.2500	0.1111	0.3333	0.3226	0.0946	82	1
LASSO	Filter	bootstrap	10	0.2167	0.0760	0.2121	0.0811	0.4815	0.3036	0.1059	78	1
BIMS-GA-NPPI	Ablation	seeds	10	0.2130	0.0628	0.2121	0.0811	0.3333	0.3000	0.0927	77	0
LassoNet	Deep	bootstrap	10	0.1840	0.0583	0.1765	0.0526	0.3333	0.2571	0.0901	89	0
MOEA/D	Pareto MO	seeds	10	0.1699	0.0627	0.1765	0.0811	0.3333	0.2345	0.0976	89	1
ANOVA F-test	Filter	bootstrap	10	0.1578	0.0865	0.1429	0.0256	0.3793	0.2107	0.1338	95	0
SVM-RFE	Wrapper	bootstrap	10	0.1332	0.0528	0.1429	0.0256	0.2121	0.1762	0.0898	100	0
Attention-based FS	Deep	seeds	10	0.1175	0.0418	0.1111	0.0526	0.2121	0.1512	0.0705	115	0
mRMR	Filter	bootstrap	10	0.1053	0.0437	0.1111	0.0256	0.2121	0.1298	0.0750	106	0
Binary GWO	Metaheuristic	seeds	10	0.0774	0.0402	0.0811	0.0000	0.1429	0.0798	0.0743	123	0
Binary PSO	Metaheuristic	seeds	10	0.0741	0.0387	0.0811	0.0000	0.1429	0.0738	0.0729	126	0
MLP (perm. importance)	ML importance	seeds	10	0.0477	0.0346	0.0526	0.0000	0.1111	0.0238	0.0677	138	0

Table S6I. STRING interaction count and network topology of the panel selected by each method

Method	Interactions	PPI coherence	Mean degree	Clustering coefficient	Modularity	Isolated genes	Maximum betweenness
SVM-RFE	1	0.0049	0.1000	0.0000	0.0000	18	0.0000
Binary GWO	5	0.0221	0.5000	0.1167	0.1929	14	0.0117
Binary PSO	2	0.0053	0.2000	0.0000	0.4871	16	0.0000
NSGA-III	37	0.1628	3.7000	0.3862	0.0504	9	0.0546
NSGA-II	36	0.1555	3.6000	0.5027	0.2088	5	0.0327
LASSO	2	0.0084	0.2000	0.0000	0.4758	16	0.0000
BIMS-GA-NPPI	4.5	0.0115	0.4500	0.0042	0.4517	13.1	0.0175
BIMS-GA (Full)	111	0.4798	11.1000	0.7203	0.1228	0	0.0887
BIMS-GA-NCI	112.0	0.4854	11.2000	0.7115	0.1245	0.0	0.0795
BIMS-GA-FW	111.7	0.4775	11.1700	0.7222	0.1279	0.0	0.0859
MOEA/D	47	0.1919	4.7000	0.5532	0.2822	3	0.2009
MLP (perm. importance)	4	0.0146	0.4000	0.0000	0.4553	14	0.0117
Attention-based FS	3	0.0144	0.3000	0.1500	0.0000	17	0.0000
LassoNet	3	0.0132	0.3000	0.0000	0.4760	15	0.0058
DeepPINK	1	0.0052	0.1000	0.0000	0.0000	18	0.0000
ANOVA F-test	10	0.0510	1.0000	0.2500	0.0000	15	0.0000
Random Forest	11	0.0538	1.1000	0.2500	0.0999	13	0.0000
mRMR	3	0.0128	0.3000	0.0000	0.3398	15	0.0058

Note. The three ablation variants are averaged over several panels (Table S6F, Panels (n)), so their counts are shown to one decimal place.

Table S6J. Every method scored over ten runs rather than one run

Method	Protocol	Runs	AUC	C-index	PPI
Binary GWO	seeds	10	0.9697 ± 0.0050	0.5962 ± 0.0139	0.0127 ± 0.0090
Binary PSO	seeds	10	0.9663 ± 0.0064	0.5900 ± 0.0131	0.0116 ± 0.0083
NSGA-III	seeds	10	0.9584 ± 0.0069	0.6478 ± 0.0060	0.1213 ± 0.0328
NSGA-II	seeds	10	0.9511 ± 0.0041	0.6430 ± 0.0053	0.1500 ± 0.0321
BIMS-GA-NPPI	seeds	10	0.9497 ± 0.0088	0.6824 ± 0.0115	0.0115 ± 0.0069
MOEA/D	seeds	10	0.9473 ± 0.0111	0.6287 ± 0.0105	0.1490 ± 0.0446
LASSO	bootstrap	10	0.9470 ± 0.0108	0.5790 ± 0.0085	0.0080 ± 0.0072
BIMS-GA-NCI	seeds	10	0.9458 ± 0.0059	0.6048 ± 0.0048	0.4655 ± 0.0116
BIMS-GA (Full)	seeds	10	0.9456 ± 0.0051	0.6112 ± 0.0031	0.4558 ± 0.0234
DeepPINK	seeds	10	0.9423 ± 0.0104	0.5917 ± 0.0142	0.0094 ± 0.0097
LassoNet	bootstrap	10	0.9417 ± 0.0138	0.5845 ± 0.0108	0.0073 ± 0.0037
SVM-RFE	bootstrap	10	0.9411 ± 0.0130	0.5887 ± 0.0123	0.0064 ± 0.0062
BIMS-GA-FW	seeds	10	0.9409 ± 0.0092	0.6210 ± 0.0152	0.4048 ± 0.0921
Attention-based FS	seeds	10	0.9311 ± 0.0120	0.5774 ± 0.0120	0.0089 ± 0.0052
mRMR	bootstrap	10	0.9245 ± 0.0142	0.5875 ± 0.0151	0.0085 ± 0.0049
Random Forest	seeds	10	0.9218 ± 0.0084	0.5635 ± 0.0032	0.0321 ± 0.0142
MLP (perm. importance)	seeds	10	0.9208 ± 0.0126	0.5798 ± 0.0128	0.0103 ± 0.0084

Method	Protocol	Runs	AUC	C-index	PPI
ANOVA F-test	bootstrap	10	0.9185 ± 0.0079	0.5663 ± 0.0092	0.0426 ± 0.0206

Note. For a stochastic method the ten runs are ten seeds; for a deterministic one they are ten bootstrap resamples, since a fixed seed returns one panel. Every panel is scored on the full cohort, which is why the BIMS-GA mean here, 0.9456, is above the 0.9300 of Table S2E, where the same ten panels are scored out-of-fold. Rows are ordered by mean AUC.

Table S7. The nested cross-validation protocol.

Table S7A. Nested cross-validation: the entire selection chain repeated inside each outer training partition

Method	Folds	Mean held-out AUC	SD	Pooled out-of-fold AUC
Attention-based FS	5	0.9037	0.0241	0.9000
LassoNet	5	0.9048	0.0358	0.8983
SVM-RFE	5	0.9037	0.0336	0.8974
LASSO	5	0.9013	0.0110	0.8952
Binary GWO	5	0.8977	0.0520	0.8931
ANOVA F-test	5	0.8990	0.0282	0.8921
mRMR	5	0.8966	0.0245	0.8921
Binary PSO	5	0.8810	0.0418	0.8905
Random Forest	5	0.8978	0.0304	0.8893
MLP (perm. importance)	5	0.8941	0.0392	0.8888
MOEA/D	5	0.8870	0.0358	0.8871
NSGA-III	5	0.8882	0.0400	0.8857
BIMS-GA	5	0.8941	0.0530	0.8855
NSGA-II	5	0.8882	0.0553	0.8850
DeepPINK	5	0.8810	0.0293	0.8765

Note. Feature selection is re-run inside every outer training fold, using only the samples in that fold.

Table S7B. Fold-by-fold detail of the nested protocol for BIMS-GA

Fold	DE genes (training only)	Held-out AUC	Seconds
1	1736	0.8155	83.5000
2	2039	0.8631	79.8000
3	1080	0.9345	84.3000
4	898	0.9226	83.7000
5	1043	0.9349	83.3000

Table S7C. The nested protocol applied to two different estimators

Estimator	Folds	Mean held-out AUC	SD	Pooled out-of-fold AUC	Full-data AUC	Optimism
Single search per outer fold, as for every comparator	5	0.8941	0.0530	0.8855	0.9418	0.0563
Consensus of ten searches per outer fold, the rule the reported panel uses	5	0.8620	0.0972	0.8646	0.9418	0.0772

Table S8. Selection stability under perturbation of the data and of the network.

Table S8A. Stability of the selection under perturbation of the data

Panel A · Each outer fold's panel against the consensus panel

Fold	Held-out AUC	Panel size	Genes shared with consensus	Jaccard vs. consensus
1	0.8155	20	1	0.0256
2	0.8631	20	3	0.0811
3	0.9345	20	18	0.8182
4	0.9226	20	13	0.4815
5	0.9349	20	2	0.0526

Panel B · Pairwise overlap between the five fold panels

Fold a	Fold b	Size a	Size b	Jaccard	Kuncheva
1	2	20	20	0.0000	-0.0356
1	3	20	20	0.0256	0.0162
1	4	20	20	0.0256	0.0162
1	5	20	20	0.0256	0.0162
2	3	20	20	0.0811	0.1198
2	4	20	20	0.0526	0.0680
2	5	20	20	0.0526	0.0680
3	4	20	20	0.4286	0.5858
3	5	20	20	0.0526	0.0680
4	5	20	20	0.0000	-0.0356

Panel C · Summary

Quantity	Value	SD	Minimum	Maximum
Mean Jaccard of each fold panel against the consensus panel	0.2918	0.3484	0.0256	0.8182
Mean pairwise Jaccard between the five fold panels	0.0744	0.1270	0.0000	0.4286
Mean pairwise Kuncheva index between the five fold panels, corrected against the union of the five fold pools	0.0887	0.1814	-0.0356	0.5858
Mean pairwise Jaccard across ten seeds on the full cohort	0.8042	0.0653	—	—

Note. Each outer fold selects from 80% of the samples; the last row of Panel C repeats the ten-seed index on the full cohort for comparison.

Table S8B. Selection stability of the proposed method under the bootstrap protocol, beside the deterministic methods measured the same way

Method	Protocol	Runs	Jaccard	Jaccard SD	Jaccard median	Jaccard min.	Jaccard max.	Kuncheva	Kuncheva universe	Genes in all runs	Distinct genes
LASSO	bootstrap	10	0.2167	0.0760	0.2121	0.0811	0.4815	0.3036	—	1	78
BIMS-GA (Full)	bootstrap	10	0.2092	0.2147	0.1111	0.0000	0.7391	0.2512	300	0	85
LassoNet	bootstrap	10	0.1840	0.0583	0.1765	0.0526	0.3333	0.2571	—	0	89
ANOVA F-test	bootstrap	10	0.1578	0.0865	0.1429	0.0256	0.3793	0.2107	—	0	95
SVM-RFE	bootstrap	10	0.1332	0.0528	0.1429	0.0256	0.2121	0.1762	—	0	100
mRMR	bootstrap	10	0.1053	0.0437	0.1111	0.0256	0.2121	0.1298	—	0	106
BIMS-GA (Full), pool rebuilt in each resample	bootstrap	10	0.0362	0.0537	0.0256	0.0000	0.3333	0.0484	1,110	0	161

Note. The whole selection chain is repeated in each resample, as for the deterministic methods in Table S6H.

Table S8C. Selection stability under the released STRING network and under two rewirings of it

Network	Rewirings (n)	Jaccard SD across rewirings	AUC	C-index	Coherence (search network)	Coherence (released STRING)	Jaccard	Jaccard SD	Kuncheva
STRING as released	1	—	0.9416	0.6112	0.4558	0.4558	0.8042	0.0653	0.8821
Degree-preserving rewiring	5	0.1152	0.9517	0.6434	0.1792	0.1606	0.3208	0.0730	0.4440
Weight-shuffled	5	0.0556	0.9598	0.6794	0.0479	0.0058	0.2179	0.0640	0.3071

Note. Each rewiring is searched with ten seeds under otherwise identical settings. The degree-preserving rewiring keeps every gene's total interaction weight and destroys the structure; the weight-shuffled variant keeps only the distribution of weights. The last coherence column is measured on the released matrix, so panels found under a rewired network are scored against the real one.

Table S8D. Subgroup structure of the fixed-weight variant across ten seeds, with the threshold profile and the full pairwise Jaccard matrix

Panel A · Subgroups of the ten runs

Subgroup	Runs (n)	Seeds	Mean coherence	Min. coherence	Max. coherence	Mean within-subgroup Jaccard
1	7	42;49;56;63;70;91;105	0.4576	0.4015	0.4832	0.7563
2	1	84	0.3495	0.3495	0.3495	—
3	2	77;98	0.2477	0.2361	0.2593	0.5385

Note. Panel A partitions the ten runs into three subgroups. Two are the connected components of sharing at least 0.30 of the union, a split of eight and two that holds at every threshold from 0.20 to 0.40 (Panel B). Seed 84 is then separated from the eight because its coherence falls more than two standard deviations below the rest of that component.

Panel B · Subgroup count at five thresholds, showing the partition is not an artefact of the cut

Threshold	Subgroups (n)	Sizes
0.2000	2	8;2
0.2500	2	8;2
0.3000	2	8;2
0.3500	2	8;2
0.4000	2	8;2

Panel C · The full ten-by-ten pairwise Jaccard matrix the partition is derived from

Seed	42	49	56	63	70	77	84	91	98	105
42	—	0.9048	0.8182	0.6000	0.7391	0.0526	0.5385	0.7391	0.0256	0.8182
49	0.9048	—	0.8182	0.5385	0.7391	0.0526	0.5385	0.7391	0.0256	0.7391
56	0.8182	0.8182	—	0.6000	0.8182	0.0526	0.5385	0.8182	0.0256	0.8182
63	0.6000	0.5385	0.6000	—	0.6667	0.0811	0.4286	0.6000	0.0811	0.7391
70	0.7391	0.7391	0.8182	0.6667	—	0.0811	0.4815	0.9048	0.0256	0.9048
77	0.0526	0.0526	0.0526	0.0811	0.0811	—	0.1765	0.0526	0.5385	0.0811
84	0.5385	0.5385	0.5385	0.4286	0.4815	0.1765	—	0.4815	0.1765	0.5385
91	0.7391	0.7391	0.8182	0.6000	0.9048	0.0526	0.4815	—	0.0256	0.8182
98	0.0256	0.0256	0.0256	0.0811	0.0256	0.5385	0.1765	0.0256	—	0.0256
105	0.8182	0.7391	0.8182	0.7391	0.9048	0.0811	0.5385	0.8182	0.0256	—

Table S9. Classifier comparison and operating characteristics.

Table S9A. Pairwise DeLong comparisons between the five classifiers

Model A	Model B	AUC A	AUC B	Difference	p
Logistic Regression	SVM (RBF)	0.9418	0.9009	0.0409	0.0027
	Random Forest	0.9418	0.9136	0.0282	0.0218
	Gradient Boosting	0.9418	0.9033	0.0385	0.0213
	MLP	0.9418	0.9107	0.0311	0.0618
SVM (RBF)	Random Forest	0.9009	0.9136	-0.0127	0.2861
	Gradient Boosting	0.9009	0.9033	-0.0024	0.9013

Model A	Model B	AUC A	AUC B	Difference	*p*
	MLP	0.9009	0.9107	-0.0097	0.6769
Random Forest	Gradient Boosting	0.9136	0.9033	0.0103	0.4499
	MLP	0.9136	0.9107	0.0030	0.8896
Gradient Boosting	MLP	0.9033	0.9107	-0.0074	0.7240

Table S9B. Operating characteristics of each classifier at a threshold of 0.5, with 95% bootstrap intervals

Model	Accuracy	Sensitivity	Specificity	F1	MCC	TP	FP	FN	TN	PPV	NPV
Logistic Regression	0.8846 (0.8231–0.9385)	0.8841 (0.8000–0.9545)	0.8852 (0.8033–0.9643)	0.8905 (0.8281–0.9412)	0.7687 (0.6481–0.8769)	61	7	8	54	0.8971	0.8710
SVM (RBF)	0.8538 (0.7923–0.9154)	0.8696 (0.7808–0.9429)	0.8361 (0.7385–0.9231)	0.8633 (0.7967–0.9206)	0.7064 (0.5775–0.8263)	60	10	9	51	0.8571	0.8500
Random Forest	0.8692 (0.8077–0.9231)	0.8696 (0.7857–0.9429)	0.8689 (0.7778–0.9483)	0.8759 (0.8120–0.9286)	0.7378 (0.6155–0.8457)	60	8	9	53	0.8824	0.8548
Gradient Boosting	0.8538 (0.7923–0.9154)	0.8551 (0.7705–0.9325)	0.8525 (0.7586–0.9375)	0.8613 (0.7969–0.9161)	0.7069 (0.5833–0.8254)	59	9	10	52	0.8676	0.8387
MLP	0.8538 (0.7923–0.9077)	0.8696 (0.7879–0.9437)	0.8361 (0.7368–0.9231)	0.8633 (0.7967–0.9189)	0.7064 (0.5782–0.8171)	60	10	9	51	0.8571	0.8500

Note. Accuracy, sensitivity, specificity, F1 and the Matthews correlation coefficient are given with 2,000-sample bootstrap percentile intervals. Figure S4 annotates each classifier's Brier score, expected calibration error and Hosmer-Lemeshow p on the panel it plots: logistic regression 0.0946, 0.0781 and 0.356; random forest 0.1138, 0.0726 and 0.293; multilayer perceptron 0.1205, 0.0878 and below 0.001; gradient boosting 0.1287, 0.1160 and below 0.001; support vector machine 0.1219, 0.0864 and 0.073. These hold the twenty-gene panel fixed and vary the classifier, where Table S6E holds one classifier and varies the panel.

Table S10. Selection stability across ten and twenty runs, with the permutation tests.

Table S10A. Selection stability of BIMS-GA across ten runs

Panel A · Summary across the ten runs

Quantity	Value
Mean pairwise Jaccard index	0.804 ± 0.065
Mean pairwise Kuncheva index	0.882 ± 0.043
Distinct genes selected at least once	29
Genes selected in all ten runs	14
Genes selected in at least six of ten runs	20

Panel B · Every gene selected at least once

Gene	Selection frequency	Runs	In consensus
LAMC2	1.00	10	Yes
ITGB4	1.00	10	Yes
COL17A1	1.00	10	Yes
LAMA3	1.00	10	Yes
FN1	1.00	10	Yes
ITGA2	1.00	10	Yes
ITGA11	1.00	10	Yes
ITGB5	1.00	10	Yes
PLAU	1.00	10	Yes
ITGA3	1.00	10	Yes
PLEC	1.00	10	Yes
MMP14	1.00	10	Yes
LAMB3	1.00	10	Yes
ITGB1	1.00	10	Yes
SDC1	0.90	9	Yes
MET	0.90	9	Yes
CTTN	0.90	9	Yes
ACTN4	0.80	8	Yes
VCAN	0.60	6	Yes
THBS2	0.60	6	Yes
GAPDH	0.30	3	No
FLNB	0.20	2	No
CENPF	0.20	2	No
SPOCK1	0.10	1	No
RAB39B	0.10	1	No
YWHAZ	0.10	1	No
SLC9A2	0.10	1	No
ANOS1	0.10	1	No
KIF23	0.10	1	No

Note. Ten independent seeds. Kuncheva corrects for the overlap expected by chance when drawing twenty of 300.

Table S10B. Permutation tests of stability against the ablation variants

Comparison BIMS-GA (Full) vs	Mean Jaccard (full model)	Mean Jaccard (comparator)	Difference	Permutation *p*	Permutations ≥ observed	Permutations
BIMS-GA-FW	0.8042	0.4668	0.3373	0.0525	10500	200000
BIMS-GA-NCI	0.8042	0.7707	0.0335	0.2856	57110	200000
BIMS-GA-NPPI	0.8042	0.2130	0.5912	1.5×10^{-5}	2	200000

Note. Computed on the indices of Table S6H, under the protocol stated there: seeds for the stochastic methods, bootstrap resamples for the deterministic ones.

Table S10C. Permutation test over run labels, against the nine comparators run under the seed protocol

Comparison BIMS-GA (Full) vs	Protocol	Mean Jaccard (full model)	Mean Jaccard (comparator)	Ratio	Difference	Cohen's d	Permutation p	Permutations ≥ observed	Permutations
Random Forest	seeds	0.8042	0.5657	1.4200	0.2385	3.5300	1.0×10^{-5}	1	200000
NSGA-III	seeds	0.8042	0.2707	2.9700	0.5335	7.0100	2.0×10^{-5}	3	200000
NSGA-II	seeds	0.8042	0.2661	3.0200	0.5381	8.5400	1.5×10^{-5}	2	200000
DeepPINK	seeds	0.8042	0.2289	3.5100	0.5753	8.7400	5.0×10^{-6}	0	200000
MOEA/D	seeds	0.8042	0.1699	4.7300	0.6343	9.9100	5.0×10^{-6}	0	200000
Attention-based FS	seeds	0.8042	0.1175	6.8500	0.6867	12.5200	3.0×10^{-5}	5	200000
Binary GWO	seeds	0.8042	0.0774	10.3900	0.7268	13.4000	1.0×10^{-5}	1	200000
Binary PSO	seeds	0.8042	0.0741	10.8500	0.7301	13.5900	1.5×10^{-5}	2	200000
MLP (perm. importance)	seeds	0.8042	0.0477	16.8700	0.7565	14.4700	3.0×10^{-5}	5	200000

Note. BIMS-GA is contrasted only with the nine comparators whose ten runs are reseeded searches. The five deterministic methods are excluded because their ten runs are bootstrap resamples, and contrasting a seed panel against a bootstrap panel would pool the two protocols that Table S6H reports separately.

Table S10D. Selection frequency counts of BIMS-GA and nine comparators under the seed protocol.

Method	Runs	Selections	Distinct	10/10	9/10	8/10	7/10	6/10	5/10	4/10	3/10	2/10	1/10
BIMS-GA (Full)	10	200	29	14	3	1	0	2	0	0	1	2	6
Attention-based FS	10	200	115	0	0	1	2	3	1	3	6	26	73
Binary GWO	10	200	123	0	0	0	0	2	1	5	9	30	76
Binary PSO	10	200	126	0	0	0	1	0	2	3	12	27	81
DeepPINK	10	200	82	1	0	3	1	6	2	7	6	11	45
MLP (perm. importance)	10	200	138	0	0	0	0	0	0	2	12	32	92
MOEA/D	10	200	89	1	0	1	1	0	6	9	11	16	44
NSGA-II	10	200	70	1	3	0	2	3	7	5	8	11	30
NSGA-III	10	200	70	0	1	5	4	1	2	9	5	13	30
Random Forest	10	200	40	7	4	2	4	0	2	2	4	5	10

Note. Counts sum to distinct genes; frequency × count equals 200 (ten runs of twenty genes). Five deterministic methods scored over ten bootstraps select ≥ 6 times: LASSO (5), LassoNet (8), ANOVA (4), SVM-RFE (5), mRMR (2).

Table S10E. Twenty-seed replication of the stability protocol: both indices and network coherence within each block, across blocks and pooled

Arm	Quantity	Scope	Runs	Value	SD
BIMS-GA (Full)	Mean pairwise Jaccard	block 1 (seeds 42-105)	10	0.8042	—
		block 2 (seeds 112-175)	10	0.5213	—
		across blocks	20	0.6365	—
		pooled	20	0.6490	—
	Kuncheva index	block 1 (seeds 42-105)	10	0.8821	—
		block 2 (seeds 112-175)	10	0.6143	—
		across blocks	20	0.7257	—
		pooled	20	0.7364	—
	PPI coherence (per run)	block 1 (seeds 42-105)	10	0.4558	0.0234
		block 2 (seeds 112-175)	10	0.4114	0.0868
		pooled	20	0.4336	0.0659
BIMS-GA-FW	Mean pairwise Jaccard	block 1 (seeds 42-105)	10	0.4668	—
		block 2 (seeds 112-175)	10	0.6990	—
		across blocks	20	0.5824	—
		pooled	20	0.5827	—
	Kuncheva index	block 1 (seeds 42-105)	10	0.5310	—
		block 2 (seeds 112-175)	10	0.7964	—
		across blocks	20	0.6609	—
		pooled	20	0.6622	—
	PPI coherence (per run)	block 1 (seeds 42-105)	10	0.4048	0.0921
		block 2 (seeds 112-175)	10	0.4395	0.0397
		pooled	20	0.4221	0.0713
Full vs FW	Difference in mean pairwise Jaccard	pooled	20	0.0663	—
	Permutation *p* over run labels	pooled	20	0.2824	—
		ten per arm	10	0.0525	—
BIMS-GA (Full)	Permutation *p*, block difference in coherence	block 1 vs block 2	20	0.1503	—
	Between-block drop, mean pairwise Jaccard	block 1 minus block 2	20	0.2828	—
	Between-block drop, Kuncheva index	block 1 minus block 2	20	0.2679	—
	Between-block difference in mean coherence	block 1 minus block 2	20	0.0444	—
	Lowest per-run coherence	pooled	20	0.2310	—

Table S11. Functional enrichment of the panel against the candidate pool

Library	Term	Overlap	Term size in background	Odds ratio	Adjusted p	Genes
GO Biological Process 2023	Cell-Matrix Adhesion (GO:0007160)	9	13	10.3800	< 0.0001	*COL17A1;CTTN;FN1;ITGA11;ITGA2;ITGA3;ITGB1;ITGB4;ITGB5*
	Integrin-Mediated Signaling Pathway (GO:0007229)	7	10	10.5000	< 0.0001	*FN1;ITGA11;ITGA2;ITGA3;ITGB1;ITGB4;ITGB5*
	Cell-Substrate Junction Assembly (GO:0007044)	5	6	12.5000	< 0.0001	*COL17A1;CTTN;FN1;ITGB4;PLEC*
	Endoderm Formation (GO:0001706)	5	6	12.5000	< 0.0001	*FN1;ITGB5;LAMA3;LAMB3;MMP14*
	Mesodermal Cell Differentiation (GO:0048333)	4	4	15.0000	0.0001	*ITGA2;ITGA3;ITGB1;ITGB4*
	Mesoderm Formation (GO:0001707)	4	4	15.0000	0.0001	*ITGA2;ITGA3;ITGB1;ITGB4*
	Endodermal Cell Differentiation (GO:0035987)	5	7	10.7100	0.0001	*FN1;ITGB5;LAMA3;LAMB3;MMP14*
	Epidermis Development (GO:0008544)	4	5	12.0000	0.0003	*COL17A1;LAMA3;LAMB3;LAMC2*
KEGG 2021 Human	ECM-receptor interaction	12	12	15.0000	< 0.0001	*FN1;ITGA11;ITGA2;ITGA3;ITGB1;ITGB4;ITGB5;LAMA3;LAMB3;LAMC2;SDC1;THBS2*
	Focal adhesion	13	15	13.0000	< 0.0001	*ACTN4;FN1;ITGA11;ITGA2;ITGA3;ITGB1;ITGB4;ITGB5;LAMA3;LAMB3;LAMC2;MET;THBS2*
	PI3K-Akt signaling pathway	12	16	11.2500	< 0.0001	*FN1;ITGA11;ITGA2;ITGA3;ITGB1;ITGB4;ITGB5;LAMA3;LAMB3;LAMC2;MET;THBS2*
KEGG 2021 Human	Human papillomavirus infection	11	16	10.3100	< 0.0001	*FN1;ITGA11;ITGA2;ITGA3;ITGB1;ITGB4;ITGB5;LAMA3;LAMB3;LAMC2;THBS2*
	Small cell lung cancer	7	7	15.0000	< 0.0001	*FN1;ITGA2;ITGA3;ITGB1;LAMA3;LAMB3;LAMC2*
	Regulation of actin cytoskeleton	8	12	10.0000	< 0.0001	*ACTN4;FN1;ITGA11;ITGA2;ITGA3;ITGB1;ITGB4;ITGB5*
	Proteoglycans in cancer	8	14	8.5700	< 0.0001	*CTTN;FN1;ITGA2;ITGB1;ITGB5;MET;PLAU;SDC1*
	Amoebiasis	5	5	15.0000	< 0.0001	*ACTN4;FN1;LAMA3;LAMB3;LAMC2*
Reactome 2022	Extracellular Matrix Organization R-HSA-1474244	14	23	9.1300	< 0.0001	*COL17A1;FN1;ITGA11;ITGA2;ITGA3;ITGB1;ITGB4;ITGB5;LAMB3;LAMC2;MMP14;PLEC;SDC1;VCAN*

Library	Term	Overlap	Term size in background	Odds ratio	Adjusted *p*	Genes
	Non-integrin membrane-ECM Interactions R-HSA-3000171	7	7	15.0000	< 0.0001	*ITGA2;ITGB1;ITGB4;ITGB5;LAMB3;LAMC2;SDC1*
	Laminin Interactions R-HSA-3000157	6	6	15.0000	< 0.0001	*ITGA2;ITGA3;ITGB1;ITGB4;LAMB3;LAMC2*
	Integrin Cell Surface Interactions R-HSA-216083	6	6	15.0000	< 0.0001	*FN1;ITGA11;ITGA2;ITGA3;ITGB1;ITGB5*
	Syndecan Interactions R-HSA-3000170	5	5	15.0000	< 0.0001	*ITGA2;ITGB1;ITGB4;ITGB5;SDC1*
	MET Activates PTK2 Signaling R-HSA-8874081	5	5	15.0000	< 0.0001	*ITGA2;ITGA3;ITGB1;LAMB3;LAMC2*
	Type I Hemidesmosome Assembly R-HSA-446107	5	5	15.0000	< 0.0001	*COL17A1;ITGB4;LAMB3;LAMC2;PLEC*
	Cell-Cell Communication R-HSA-1500931	7	13	8.0800	< 0.0001	*ACTN4;COL17A1;ITGB1;ITGB4;LAMB3;LAMC2;PLEC*

Note. The background is the 300-gene candidate pool, not the whole transcriptome, so the terms reported are those the panel is enriched for relative to the genes it was selected from. Adjusted p is printed to four decimal places and reads < 0.0001 for every term above the dashed rule in Figure 5; the figure plots the negative base-ten logarithm of the unrounded value, which is 7.06 for cell-matrix adhesion, 5.39 for integrin-mediated signalling, 4.34 for cell-substrate junction assembly and for endoderm formation, and 11.45, 7.29, 6.26 and 6.26 for the four Reactome pathways.
